# Melatonin Activation by Cytochrome P450 Isozymes: How Does CYP1A2 Compare to CYP1A1?

**DOI:** 10.3390/ijms24043651

**Published:** 2023-02-11

**Authors:** Thirakorn Mokkawes, Sam P. de Visser

**Affiliations:** 1 Manchester Institute of Biotechnology, The University of Manchester, 131 Princess Street, Manchester M1 7DN, UK; 2 Department of Chemical Engineering, The University of Manchester, Oxford Road, Manchester M13 9PL, UK

**Keywords:** enzyme catalysis, molecular dynamics, quantum mechanics, inorganic reaction mechanisms, hydroxylation, regioselectivity

## Abstract

Cytochrome P450 enzymes are versatile enzymes found in most biosystems that catalyze mono-oxygenation reactions as a means of biosynthesis and biodegradation steps. In the liver, they metabolize xenobiotics, but there are a range of isozymes with differences in three-dimensional structure and protein chain. Consequently, the various P450 isozymes react with substrates differently and give varying product distributions. To understand how melatonin is activated by the P450s in the liver, we did a thorough molecular dynamics and quantum mechanics study on cytochrome P450 1A2 activation of melatonin forming 6-hydroxymelatonin and *N*-acetylserotonin products through aromatic hydroxylation and *O*-demethylation pathways, respectively. We started from crystal structure coordinates and docked substrate into the model, and obtained ten strong binding conformations with the substrate in the active site. Subsequently, for each of the ten substrate orientations, long (up to 1 μs) molecular dynamics simulations were run. We then analyzed the orientations of the substrate with respect to the heme for all snapshots. Interestingly, the shortest distance does not correspond to the group that is expected to be activated. However, the substrate positioning gives insight into the protein residues it interacts with. Thereafter, quantum chemical cluster models were created and the substrate hydroxylation pathways calculated with density functional theory. These relative barrier heights confirm the experimental product distributions and highlight why certain products are obtained. We make a detailed comparison with previous results on CYP1A1 and identify their reactivity differences with melatonin.

## 1. Introduction

The human body contains active machineries to deal with toxic xenobiotic compounds which harm cells or disrupt chemical processes [1]. In particular, the cytochrome P450 enzymes are a broad class of heme mono-oxygenases in the body that have evolved to metabolize a large variety of endogenous and exogenous substrates in the body [2,3,4,5,6,7,8,9,10,11]. The members of the P450 superfamily currently count over 21,000 structures and are found in most biosystems [12]. They have been classified into 18 families and 57 subfamilies that share 40% and 60% homology in their amino acid sequence [13,14]. Structurally, all P450s have a central iron bound inside a heme structure that, in their catalytic cycle, uses one molecule of dioxygen, two electrons from a redox partner and two protons from the solvent. In general, one oxygen atom of O_2_ is transferred to the substrate [2,3,4,5,6,7,8,9,10,11,15], while the other oxygen atom is reduced to a water molecule, although there is also evidence of desaturation reactions whereby both oxygen atoms of O_2_ reduce to water molecules [16,17].

In the resting state, the iron atom is in the ferric state with a six-coordinate binding orientation through four linkages with the four heme nitrogen atoms, a covalent bond with the thiolate sulfur atom from a cysteinate ligand in the axial position and a bound water molecule on the distal site [18,19,20,21]. The cysteinate ligand is the conserved residue in the P450 structure that links the heme to the protein. It facilitates the generation of the active oxidant in the catalytic cycle, namely the iron (IV)-oxo heme cation radical species that is called Compound I (Cpd I) [2,3,4,5,22,23,24]. The overall oxygen atom insertion into the substrate leads to aromatic and aliphatic hydroxylation reactions, while the other oxygen atom originating from O_2_ is reduced to a water molecule [22,23,24,25,26,27]. In general, the oxidizing capability of the P450 enzyme is highly broad and covers a wide range of chemical compounds. As such, the P450s have been shown to catalyze diverse oxidation reactions, including aromatic and aliphatic hydroxylation, *N*- and *O*-dealkylation, epoxidation, sulfoxidation and desaturation [23,25,26,27,28,29,30,31,32,33,34,35,36,37].

The CYP1 family, which is the focus of this work, comprises three members, namely CYP1A1, CYP1A2 and CYP1B1. These enzymes have a broad substrate range and target various xenobiotics in the body. CYP1A1 has about 70% structural identity with CYP1A2, while the amino acid sequence identity of CYP1B1 with CYP1A1 and CYP1A2 are 38% and 37%, respectively [38,39]. CYP1A1 is mostly found in the extrahepatic tissues, including the skin, the lungs and the brain. CYP1A1 and CYP1A2 have been evolved to act as metabolic enzymes and generally react through the hydroxylation and oxidation of polycyclic aromatic hydrocarbons. CYP1A1 and CYP1B1, specifically, are involved in the metabolism of pro-carcinogens into carcinogens via an epoxidation reaction that is known as cancer initiation [40]. Some aromatic substrates that are metabolized by all CYP1 enzymes include melatonin, caffeine, ethoxyresorufin and estrogen [41,42,43,44,45,46]. Among the three enzymes, CYP1A2 is the most important enzyme implicated in the metabolism of drug molecules, and reports have identified it in the reactions of phenacetin, clozapine, mexiletine and propranolol [47].

**Figure 1:**
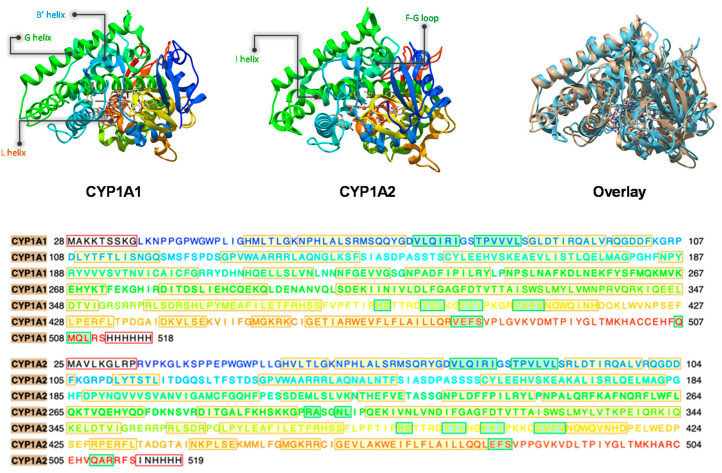
Structures of CYP1A1 (left), CYP1A2 (middle) and their overlay (right) as taken from the 6DWN and 2HI4 pdb files. The bottom shows the amino acid sequences for CYP1A1 and CYP1A2 for comparison.

The three-dimensional structure of CYP1A1 and CYP1A2 are displayed in Figure 1 as taken from protein databank (PDB) files [18,48,49]. Generally, the cytochrome P450 CYP1 isozymes are built up from twelve helices, labelled A–L, and four β-sheets which form the bulk of the protein. The heme active site is housed over the L-helix and close to the I-helix, which is placed perpendicularly to the F-G segment. The substrate entrance into the active site is governed by residues on the B-, C-, F- and G-helices. The overlay of the CYP1A1 and CYP1A2 structures highlight their similarity and differences in three-dimensional structures (Figure 1). The residues and helices surrounding the heme cofactor contain many conserved regions between the various P450 isozymes that are involved in proton relay into the active site and important catalytic cycle events. Particularly, the I- and L-helices contain highly conserved areas due to the fact that these residues are implicated with Cpd I formation [50]. Thus, in CYP1A1, the I-helix is located above the heme and includes the conserved region starting from Gly_316_ to Thr_321_. This part of the I-helix is expected to interact with the substrate and positions it with hydrogen bonding interactions at the early stages of the catalytic cycle. The I-helix has also been implicated with the dioxygen entrance channel and is expected to guide the dioxygen molecule toward the heme [51,52], which is required for Cpd I formation. The dioxygen molecule has been proposed to be held in position through a hydrogen bond from Thr_321_. After the dioxygen binding, this helix is slightly opened, which may result in additional water molecules entering the active site [53]. One of these water molecules can provide a hydrogen bond to the distal oxygen atom of the ferric-peroxo complex [54], and initiates the heterolytic cleavage of the dioxygen O−O bond in the ferric-hydroperoxo species to generate Cpd I. As for the L-helix, it includes the cysteine axial ligand bound to the heme. The Fe−S bridge from the thiolate sulfur of Cys_457_ and central iron atom promotes the sharing of delocalized oxidizing equivalents with the heme to form the Cpd I [55,56]. Further, the electrostatic environment of the Fe−S bridge is maintained by a number of N−H hydrogen bond donations to the thiolate of the cysteinate axial ligand, and these interactions may determine a suitable electron donation (push effect) and thereby optimize Cpd I performance [56,57,58,59].

Due to differences in protein chains, the active site volumes, i.e., substrate-binding pocket, of the various CYP1 isozymes differ dramatically. Thus, the active site volumes of CYP1A1, CYP1A2 and CYP1B1 are 524, 469 and 441 Å^3,^ respectively [38]. Although CYP1A2 has a smaller active site volume as compared to CYP1A1, its flexibility of the active site is better because of a break in the F-helix similarly to CYP1A1. It is impacted by the side chains pointing into the active site which are smaller in CYP1A1. The area above the heme is determined by Val_382_ in CYP1A1 and Val_395_ in CYP1B1, while this residue is absent in CYP1A2 and replaced by Leu_382,_ leading to a smaller active site cavity.

**Figure 2:**
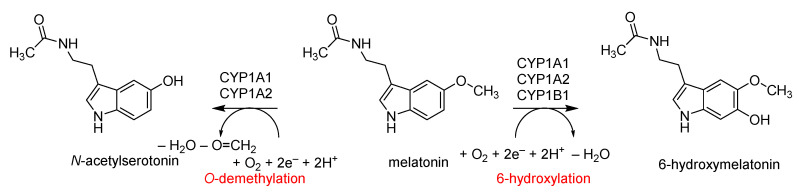
Metabolism pathways of melatonin in the body and reaction products obtained.

Melatonin is metabolized in the human organism in the liver, skin, lung and brain through the CYP1A1, CYP1A2 and CYP1B1 isozymes. Endogenous and exogenous melatonin is degraded to 6-hydroxymelatonin and *N*-acetylserotonin by aromatic hydroxylation and *O*-demethylation, respectively [60,61,62,63,64], as shown in Figure 2. The mechanism of *O*-demethylation is initiated by a hydrogen atom abstraction from the methoxy group of melatonin to the oxygen atom of Cpd I, which generates a substrate radical side chain and Cpd II [65,66,67,68,69]. The latter, through OH rebound to the radical, gives hydroxylated methoxy group products. The hydroxylated product is converted into *N*-acetylserotonin through the release of formaldehyde, which is expected to occur outside of the enzyme and assisted by water molecules or a base [66]. The alternative reaction pathway is aromatic hydroxylation, where an electrophilic attack of the oxygen atom belonging to Cpd I on the C^6^ carbon atom of melatonin generates the σ-intermediate with covalent C−O linkage between the heme and substrate [70,71,72,73,74,75,76]. The *ipso*-proton of that intermediate is transferred to the porphyrin complex and forms the protonated porphyrin intermediate, which then re-shuttles that proton to the oxygen atom to form the phenol group and finally releases 6-hydroxymelatonin.

The various P450 isozymes react differently with melatonin, namely CYP1A1, CYP1A2 and CYP1B1 give dominant 6-hydroxylation, while CYP2C19 produces *O*-demethylation products selectively. Nevertheless, with CYP1A2 some *O*-demethylation products are also observed. From Michael–Menton kinetics the K_M_ and v_max_ values of melatonin activation by CYP1A1, CYP1A2 and CYP1B1 were determined and values of K_M_ = 19.2, 25.9 and 30.9 μM and v_max_ = 6.46, 10.6 and 5.3 pmol min^−1^ pmol^−1^ P450, respectively, were obtained [77]. To understand the differences of kinetics and product distributions between the various P450 isozymes, we decided to perform a detailed computational study and compare melatonin binding and reactivity in CYP1A1 versus CYP1A2 enzymes. Our combined molecular mechanics, molecular dynamics and density functional theory study shows the differences in substrate binding, positioning and mobility in the active site and highlights the differences in product distributions.

## 2. Results

### 2.1. MD Simulations on Susbtrate Binding and Positioning in CYP1A2

**Figure 3:**
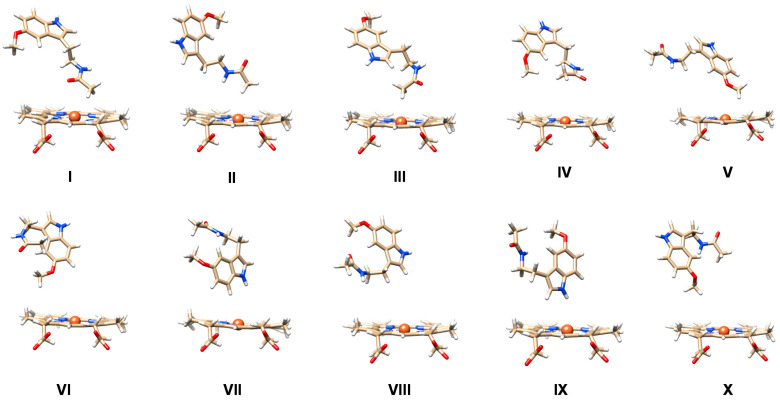
The ten lowest-energy docking poses (**I**–**X**) for melatonin in the substrate-binding pocket of CYP1A2. Substrate orientation is show with respect to the heme.

We started the work from the crystal structure coordinates of CYP1A2 and melatonin as deposited under the 2HI4 and ML1 pdb files in the protein databank [18,49]. The substrate was removed from the CYP1A2 pdb file and the resting state heme converted into a Cpd I form by binding an oxygen atom to iron at a distance of 1.686 Å. Hydrogen atoms were added as specified in the Methodology. Subsequently, the Autodock Vina software package [78] was used to dock melatonin into the substrate-binding pocket, whereby a grid box containing the area bound by Cpd I and the I- and G-helix was applied. As melatonin only has two weak N−H bonds for hydrogen-bonding donation interactions with protein and a carbonyl and an ether group for accepting hydrogen bond interactions, it may be relatively weakly bound in the P450 enzyme structure. Ten low-energy binding conformations of melatonin in the active site were found designated with Roman numerals **I**–**X** as shown in Figure 3. Thus, in configuration **I**, **II** and **III,** the substrate *N*-acetyl group of melatonin points into the substrate-binding pocket and is closest to the heme, while the indole group points away from the heme. In these orientations, the indole group is held by π-stacking interactions with Phe residues in the ceiling of the substrate-binding pocket and the I-helix. In particular, π-stacking interactions in configuration **I** are with Phe_260_ and Phe_319_, while in configuration **II** they are with Phe_226_ and Phe_260_ and in configuration **III** with Phe_226_. In the latter conformation, there is also a hydrogen bond between the indole N−H group and a nearby Thr residue (Thr_124_). In structures **I**, **II** and **III**, therefore, the C^6^ and methoxy groups are pointing away from the heme and are at a relatively large distance from Cpd I, and hence these orientations may not be suitable for melatonin activation. In orientation **IV,** the methoxy group of the substrate points slightly down into the active site but the indole group is still staggered with the I-helix and π-stacked with Phe_226_. Hence, structure **IV** may also not be catalytically active. The docked structures **V** and **VI** have the indole and methoxy groups of melatonin pointing into the substrate-binding pocket in close approach to the heme. The substrate N−H group of the *N*-acetyl substituent hydrogen bonds with the carboxylate group of Asp_313,_ and therefore is held tightly in position in structure **V**. In addition, the indole group is held in position by the side chains of Phe_125_, Phe_226_ and Leu_497_. By contrast, in orientation **VI,** the carbonyl group of the substrate is in hydrogen-bonding interaction with Ser_122_, while the indole group is held by the I-helix and residues Phe_125_, Phe_226_ and Leu_497_.

In structure **VII,** the indole group is forming a hydrogen-bonding interaction with the oxo group of Cpd I via a water molecule, but the methoxy group points up. In structures **VIII** and **IX,** the methoxy and C^6^ positions point away from the heme and the indole N−H group interacts with Thr_124_ (in structure **VIII**), while the *N*-acetyl N−H group in structure **IX** forms a hydrogen bond with the peptide bond of residue 316. The latter interaction is also seen in configuration **X,** but the indole group is flipped and pointing more downwards.

**Figure 4:**
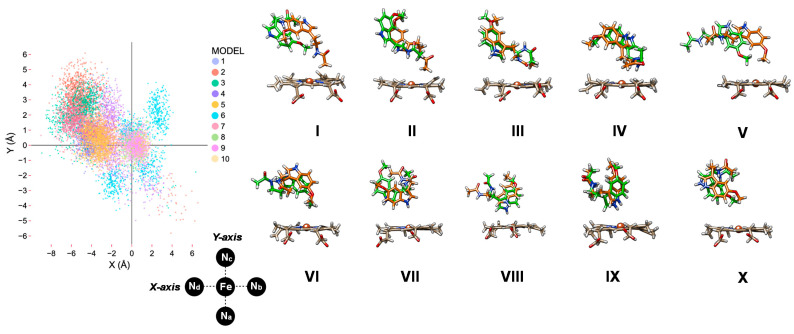
**Left**: Scatter plot of the position of the substrate position with respect to the heme. The iron is in the origin and the X- and Y-axis are along the N_d_-Fe-N_b_ and N_a_-Fe-N_c_ bonds. **Right**: Melatonin position in the start (after 1 ns in orange) and end (after 100 ns in green) configurations of the MD simulation for MD simulation **I** to **X**.

Subsequently, we took the ten melatonin-bound CYP1A2 structures and ran a 100 ns molecular dynamics (MD) simulation for each of them. For all structures, the root-mean-square-deviation (RMSD) of the MD stabilizes within 20 ns of time. In Figure 4, we show the position of melatonin with respect to the heme for these ten MD simulations, where we compare the starting and final MD frames. As can be seen, each of these ten MD simulations keeps the substrate in virtually the same position the work started off from. That means in all cases the substrate interacts strongly with protein residues and is kept tightly in its position. We then measured the position of the substrate with respect to Cpd I for all snapshots (for each 1 ns timeframe) from the ten MD simulations and extracted the nearest position with respect to the plane through the four nitrogen atoms by measuring the orthogonal distance from the plane to the substrate. The substrate orientation as projected on the plane of the heme is shown on the left-hand side of Figure 4. Most data points are located in the quadrant bound by the N_c_-Fe-N_d_ atoms. This is the area close to the I-helix that has been implicated with substrate binding and positioning [52]. Indeed, interactions between the substrate and the residues Asn_312_, Asp_313_ and Asp_320_ are seen. In general, the distribution bands for each MD run give a narrow bandwidth, where the substrate is rigid during most of the simulation. Interestingly, when we run an MD simulation for the product-bound complexes, the pattern looks different and no distribution directly above the heme iron or plane above the N_a_-Fe-N_b_ quadrant is seen. On the other hand, more binding is seen close to the positive Y-axis. These studies implicate that product binds differently from reactant systems when only one extra alcohol group is present in the product that is missing in melatonin substrate. It will also enable product release possible.

### 2.2. QM Cluster Calculations on CYP1A2 Mechanism of Melatonin Activation

**Figure 5:**
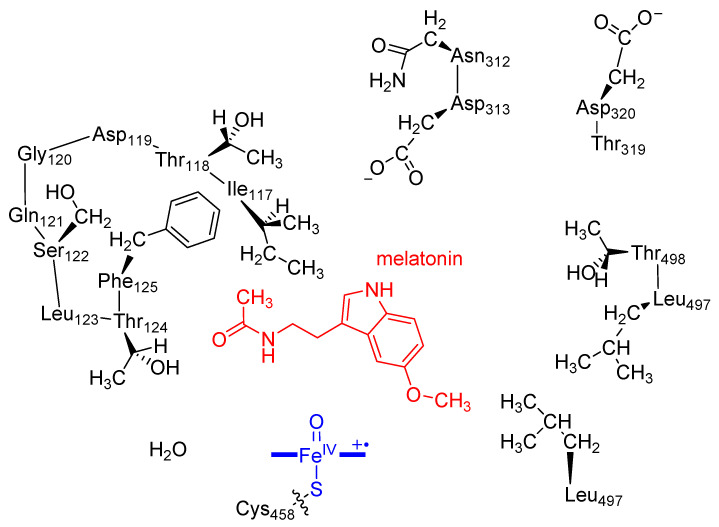
QM cluster model utilized in this work with melatonin (in red) and Cpd I (in blue). Wiggly lines represent bonds that were broken and truncated with a hydrogen atom. No atoms were fixed during the geometry optimizations.

As second coordination sphere effects [79,80] in proteins are important and often influence regioselectivity patterns, we created a quantum mechanical (QM) cluster model of the CYP1A2 active site with melatonin bound and selected for the 80.4 ns snapshot of MD run **VI**. This is the lowest-energy substrate-bound structure and has the substrate positioned close to the heme with the methoxy group pointing into the substrate-binding pocket with a distance of 3.4 Å of the oxo group to the nearest hydrogen atom. The C^6^-position of the aromatic ring is somewhat further away at 6.4 Å in this orientation, but still should be accessible for aromatic hydroxylation after rotation of the substrate. No low-energy conformations during the MD simulation had the substrate closer or in alternative conformations; hence, only one cluster model was created and studied here. A cluster model was selected based on the heme and substrate orientation in the 80.4 ns snapshot of MD run **VI**. These cluster models have been used before extensively [81,82], and include the oxidant, substrate, and the hydrogen-bonding and steric residues of the substrate-binding pocket that determine the positioning of substrate and oxidant. We selected Cpd I, the substrate and large part of the second-coordination sphere that determines substrate positioning and orientation. Figure 5 shows the cluster model used in this work. The heme was truncated and all side chains replaced by hydrogen atoms, while the axial cysteinate group was represented by thiolate. Several protein chains were included in the model, namely the chain Ile_117_-Thr_118_-Asp_119_-Gly_120_-Gln_121_-Ser_122_-Leu_123_-Thr_124_-Phe_125_, Asn_312_-Asp_313_, Thr_319_-Asp_320_ and Leu_497_-Thr_498_, whereby the terminal C−C^α^ bond was replaced by C−H. The amino acid residues Asp_119_, Gln_121_ and Leu_123_ were truncated to a Gly residue. This system has 283 atoms, has overall charge of −2 and was calculated in the doublet and quartet spin states. Previous computational studies [25,59] showed that Cpd I has close-lying doublet and quartet spin configurations that each react with substrate to form products. As sometimes the barriers are different on the individual spin state surfaces, this leads to two-state-reactivity, where patterns on the doublet and quartet spin state are searched.

**Figure 6:**
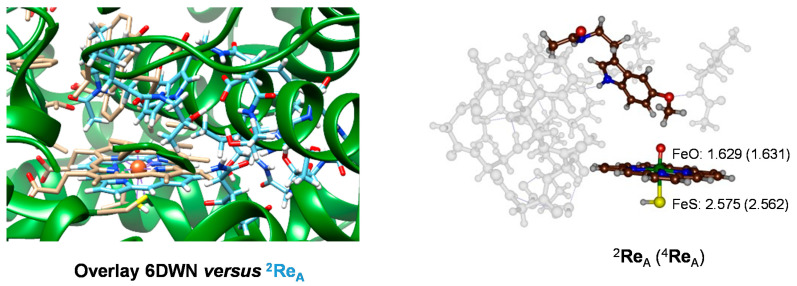
DFT optimized geometries of the reactant cluster models of CYP1A2 with melatonin bound (^2,4^**Re**_A_). Optimized geometries give bond lengths in angstroms. The left-hand side gives an overlay of the DFT-optimized geometry with the crystal structure coordinates from 6DWN.

Subsequently, we ran full geometry optimizations of the Cpd I with substrate cluster models of CYP1A2 designated reactants **Re**_A_. Figure 6 shows the optimized geometries of the melatonin-bound reactant complexes ^2,4^**Re**_A_ as obtained using density functional theory (DFT) methods in the doublet and quartet spin states as identified with a superscript after the label. The Fe−O distance is short, namely 1.629 Å for ^2^**Re**_A_ and 1.631 Å for ^4^**Re**_A_. These distances are in good agreement with those reported experimentally [22] as well as with those calculated before for alternative P450 complexes [24,25,34,83,84,85,86,87,88,89,90,91,92,93,94,95,96]. In addition, the calculated Fe−S distance of 2.575 Å (doublet) and 2.562 Å (quartet) is well within the range of calculated structures reported previously. As such, the optimized geometries match previous calculations on alternative P450 isozymes well. To find out how well the structure compares to the crystal structure coordinates of the 6DWN pdb file [18,48], we show the overlay of the optimized geometry with the pdb on the left-hand side of Figure 6. As can be seen, most residues are in virtually the same position. In particular, the I-helix residues follow the chain in the pdb well and most residues are located in a similar orientation. Therefore, our cluster model is a good mimic of the CYP1A2 active site structure, and is a good representation of the enzymatic structure.

Next, we calculated the aromatic hydroxylation pathway of the C^6^-position of melatonin by CYP1A2 Cpd I. The aromatic hydroxylation mechanism is well established [70,71,72,73,74,75,76], and starts with the electrophilic addition of the oxo group of Cpd I to the C^6^-carbon atom of the substrate via transition state **TS1**_C6_ to form the σ-intermediate **IM1**_C6_. The latter can have a radical on the substrate through a one-electron transfer process from substrate to Cpd I or a cationic substrate ring through a two-electron transfer step. Thereafter, the substrate releases a proton and restores the aromaticity in the indole group via transition states **TS2**_PT_ and forms the proton-transfer intermediate **IM2**. In **IM2,** the porphyrin ring is protonated and this proton is shuttled back to substrate via transition state **TS3**_PT_ onto the phenolate oxygen to form C^6^-hydroxymelatonin products **P**_C6_.

**Figure 7:**
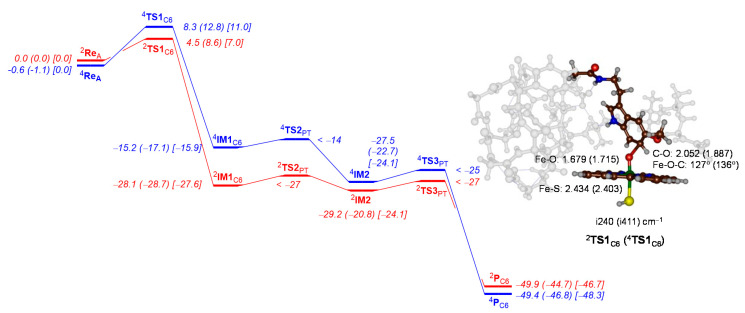
DFT-calculated C^6^-aromatic hydroxylation pathway for the reaction of CYP1A2 Cpd I with melatonin. The potential energy landscape gives enthalpies (out of parenthesis) and free energies (in parenthesis) in kcal mol^−1^. In square brackets free energies with quasi-harmonic corrections are given. Enthalpies are UB3LYP/BS2 energies corrected with zero-point energies, while free energies also contain thermal, solvent and entropic corrections at 298 K. The landscape gives quartet spin data in blue and doublet spin data in red. Optimized geometries of the transition states give bond lengths in angstroms, angles in degrees and the imaginary frequency in cm^−1^.

Figure 7 shows the calculated potential energy profile for C^6^-hydroxylation of melatonin by CYP1A2 Cpd I. The initial electrophilic addition step is rate determining and both proton transfer barriers (**TS2** and **TS3**) are small. As a matter of fact, we were unable to characterize these transition states fully, but constraint geometry scans establish low-energy pathways. In the doublet spin state, the barrier ^2^**TS1**_C6_ is ∆E + ZPE = 4.5 kcal mol^−1^ in energy, while it is 8.3 kcal mol^−1^ on the quartet spin state surface. These are low barriers and considerably lower in energy than those obtained for analogous substrates such as toluene and ethylbenzene [97,98,99]. Clearly, the substrate positioning is ideal for aromatic hydroxylation of melatonin. Interestingly, the barrier is also notably lower in energy than that found for our CYP1A1 model, where barriers of 10.0 (doublet) and 13.7 (quartet) kcal mol^−1^ were found [65]. The first and the last steps in the mechanism release a considerable amount of energy and lead to **IM1** and products with high exothermocity. As can be seen from Figure 7, the trends are reproduced well when free energies are used rather than ∆E + ZPE values and the ordering of the barriers stays the same. Furthermore, minor changes are observed when quasi-harmonic corrections are applied to the free energies.

The optimized geometry of the transition state structures ^2^**TS1**_C6_ and ^4^**TS1**_C6_ are shown on the right-hand side of Figure 7. Both structures have an imaginary frequency for the C−O stretch vibration and implicate oxygen atom transfer. The values of i240 and i411 cm^−1^ are typical for aromatic hydroxylation barriers [65,70,71,72,73,74,75,76]. The C−O bond lengths are relatively long, at 2.052 Å in the doublet spin state and 1.887 Å in the quartet spin state. At the same time, the Fe−O interaction has elongated somewhat to 1.679 Å in ^2^**TS1**_C6_ and 1.715 Å in ^4^**TS1**_C6_. The Fe−O−C angle is relatively bent as often is the case for aromatic hydroxylation barriers with angles of 127° (doublet) and 136° (quartet).

Subsequently, we investigated *O*-demethylation of melatonin by CYP1A2. As shown previously [65,66,67,68,69], the *O*-demethylation starts with the aliphatic hydroxylation of the methoxy group of melatonin and is followed by deformylation through a solvent (and/or proton) assisted step. The latter is expected to happen rapidly in solution and previous calculations reported small barriers for this reaction step [65,66,67,68,69]. Only the aliphatic hydroxylation takes place in the protein; hence, we focus on this step solely. The hydrogen atom abstraction transition states from the methoxy group are ∆E + ZPE = 16.5 kcal mol^−1^ in the doublet spin state and 18.8 kcal mol^−1^ in the quartet spin state. These small energy differences are commonly observed in aliphatic hydrogen atom abstraction barriers [92,93,94,95,96,97,98,99] as the same electron transfer takes place and both reactants and radical intermediates have the same orbital occupation.

Although the hydrogen atom abstraction is endothermic, there is only a small barrier for OH rebound, which we were unable to characterize. However, the system quickly relaxes to the alcohol product complexes that are energetically well below starting reactants by 47.6 (quartet) and 52.0 (doublet) kcal mol^−1^. Previous work for melatonin activation by CYP1A1 model also found small barriers for OH rebound steps [65], in addition, to a small endothermicity leading to radical intermediates. The latter is expected, as the energy between the reactants is complex, and **IM1** should be equal to the energy of the C−H bond of the substrate that is broken minus the strength of the O−H bond that is formed [97,98]. Clearly, environmental perturbations make little differences between CYP1A1 and CYP1A2 for the aliphatic hydrogen atom abstraction step of the methoxy group.

**Figure 8:**
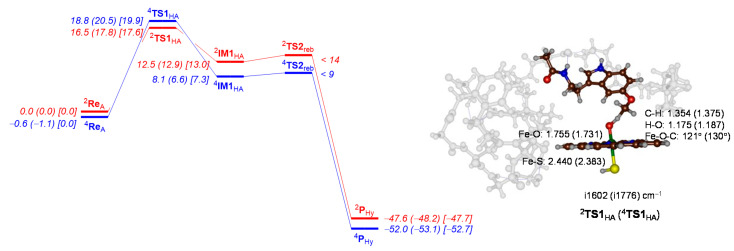
DFT-calculated methoxy group hydroxylation pathway for the reaction of CYP1A2 Cpd I with melatonin. The potential energy landscape gives enthalpies (out of parenthesis) and free energies (in parenthesis) in kcal mol^−1^. Enthalpies are UB3LYP/BS2 energies corrected with zero-point energies, while free energies (in parenthesis) contain thermal, solvent and entropic corrections at 298 K. The landscape gives quartet spin data in blue and doublet spin data in red. In square brackets free energies with quasi-harmonic corrections are given. Optimized geometries of the transition states give bond lengths in angstroms, angles in degrees and the imaginary frequency in cm^−1^.

The hydrogen atom abstraction transition states are shown on the right-hand side of Figure 8, and as expected have a large imaginary frequency for the O−H−C stretch vibration. The structures are product-like, with long C−H distances of 1.354 Å (doublet) and 1.375 Å (quartet), while the O−H distances are short: 1.175 Å for the doublet and 1.187 Å for the quartet spin state structure.

## 3. Discussion

**Figure 9:**
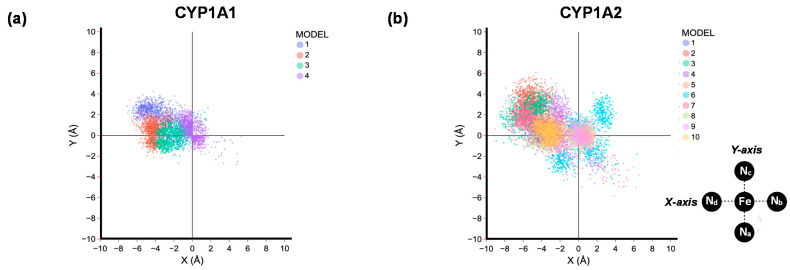
Scatter plot of the position of the substrate position with respect to the heme for CYP1A1 (**a**) and CYP1A2 (**b**) as obtained from 100 ns MD simulations with the substrate in different starting orientations. The iron is in the origin and the X- and Y-axis are along the N_d_-Fe-N_b_ and N_a_-Fe-N_c_ bonds.

In this work, we report a detailed molecular dynamics and quantum mechanics study into melatonin activation by CYP1A2 isozymes, as previously we studied the reaction in a CYP1A1 model [65]. These two enzymes have considerably different active site volumes (524 Å^3^ for CYP1A1 versus 469 Å^3^ for CYP1A2 [38]), and hence a larger substrate-binding pocket may give more flexibility and lead to different product distributions. Figure 9 shows the substrate positioning during the MD simulations for CYP1A1 and CYP1A2, where we calculated the position of substrate with respect to the heme. Each datapoint in Figure 9 represents a frame from a set of 100 ns MD simulations, i.e., a 100 ps snapshot. In general, however, the root-mean-square deviation (RMSD) of the MD simulations on CYP1A1 gives higher values than those for CYP1A2, which implicates more flexibility of the substrate and protein moieties in the CYP1A1 structure. Therefore, the substrate-bonding pocket in CYP1A2 is tighter than is the case in CYP1A1. Particularly large flexibility in the F-G loop and F- and G-helix is seen in CYP1A1 as compared to CYP1A2. Consequently, major differences in the dynamics of CYP1A1 and CYP1A2 is observed, which will result in differences in substrate activation and enzymatic turnover.

Figure 9 compares the substrate-binding positions in the active site as taken from the MD simulations for CYP1A1 and CYP1A2. As can be seen from Figure 9, in most substrate-binding positions, the substrate is located in the second quadrant (negative X, positive Y), which is the area bordered by the I-helix. Note that the scatter plot for CYP1A1 put all substrate positions maximally 4 Å away from the center of the heme along the positive Y-axis, while it is almost 6 Å for some of the substrate orientations in CYP1A2. Very few substrate orientations are located for CYP1A1 outside the second quadrant.

**Figure 10:**
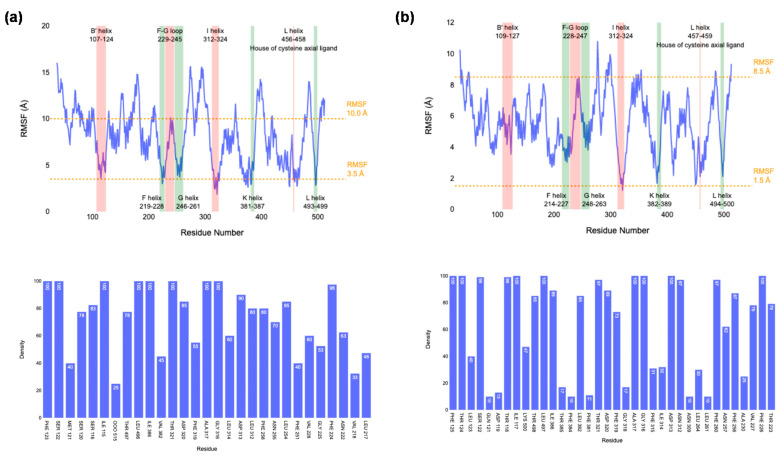
Top: RMSF values for residues of the protein of CYP1A1 (**a**) and CYP1A2 (**b**) during a 1 μs MD simulation. Specific residues and chains lining the substrate-binging pocket are highlighted. Bottom: Frequency of encountering amino acid residues with 5 Å from the substrate in the MD snapshots.

To gain further insight into substrate interactions with the protein, we ran expanded MD simulations of model **II** of CYP1A1 from Ref [65] and model **VI** from Figure 4 to 1 μs. Generally, very few differences were seen, and the active site and substrate-binding pocket remained closed for both 1 μs MD simulations, with no substrate escape observed. To further analyze the substrate distribution patterns and its interactions with protein residues, we show in Figure 10 the root-mean-square fluctuations (RMSF) of a 1 μs MD simulation for CYP1A1 and CYP1A2, while at the bottom is given a histogram with the appearance of residues within a 5 Å radius around the substrate. In general, a larger RMSF value for CYP1A1 than CYP1A2 is seen, which is in line with the larger substrate-binding pocket and the enhanced mobility in the protein structure. Most residues in the B’-helix, F-helix, G-helix and I-helix bordering the substrate-binding pocket have relatively low RMSF values, while enhanced values are seen for the F-G loop. This is the area where the substrate is expected to enter the heme-binding pocket. These RMSF values, therefore, show that the general shape of the substrate-binding pocket and positioning of the substrate will be analogous for the two isozymes.

The bottom of Figure 10 displays a histogram with probabilities of locating amino acid residues within 5 Å of the substrate as determined from the MD snapshots for the 1 μs MD simulations. Similar residues in CYP1A1 and CYP1A2 border the substrate and the I-helix residues of CYP1A2, namely Asn_312_, Asp_313_, Gly_316_, Ala_317_ and Thr_321_, which are located near substrate in more than 97% of snapshots. By contrast, these residues in CYP1A1 are in less than 80% of snapshots within a 5 Å radius. These numbers imply that substrate is tighter bound in CYP1A2 and corresponds with a smaller substrate-binding pocket that keeps the substrate in position more effectively. As such, in CYP1A1 there is more flexibility in substrate binding, and hence substrate catalysis will be determined by the weakest bond of the substrate. On the other hand, in CYP1A2, the substrate-binding pocket is tighter and positions it in a specific orientation for catalysis.

Subsequently, a series of DFT calculations were performed on models of the active sites of CYP1A1 [65] and CYP1A2 [this work] with melatonin bound. Reaction mechanisms for *O*-demethylation and C^6^-hydroxylation were calculated and the key results of the rate-determining barriers for both sets of data are shown in Table 1. In all cases, the initial step is rate determining, namely hydrogen atom abstraction for *O*-demethylation and C−O bond formation for aromatic hydroxylation. As such, the same mechanism is found for all models and methods, but differences in kinetics are seen due to differences in second-coordination sphere and substrate positioning. All calculations have an initial reaction step as rate-determining either for hydrogen atom abstraction from the methoxy group (via **TS1**_HA_) or through electrophilic addition of the oxo group to the C^6^-carbon atom (via **TS1**_C6_). For CYP1A1 two models were created based on substrate-binding orientations. Model I gives dominant C^6^-hydroxylation with the **TS1**_C6_ barriers well below the **TS1**_HA_ barriers. By contrast for Model II, the lowest barriers were obtained for hydrogen atom abstraction from the methoxy group with ∆E + ZPE = 3.3 kcal mol^−1^ in the doublet spin state, while the quartet spin barrier was ∆E + ZPE = 6.7 kcal mol^−1^. As such, a mixture of products is predicted depending on the substrate-binding orientation in the active site. For CYP1A2, a low barrier for C^6^-activation of ∆E + ZPE = 4.5 (doublet) or 8.3 (quartet) kcal mol^−1^ is obtained, which implicates dominant C^6^-hydroxylation products. This is in agreement with experimental studies of Ma et al. [77] that determined almost 100% aromatic hydroxylation products for CYP1A2, while for CYP1A1 only 77% aromatic hydroxylation products were obtained. Clearly, the tight substrate-binding pocket in CYP1A2 guides the substrate C^6^-position towards the heme, and triggers a chemoselective C^6^-hydroxylation reaction. By contrast, the substrate-binding pocket in CYP1A1 is more open, and hence multiple substrate orientations are possible, thereby triggering side reactions which lead to methoxy group hydroxylation and subsequent *O*-demethylation.

## 4. Materials and Methods

Full details of the raw data and set-up and analysis procedures are given in the Supporting Information Tables S1–S15 and Figures S1–S25. We will focus here on the main trends and results, however.

The molecular dynamics (MD) set-up was started from the published CYP1A2 and melatonin structures as taken from the Protein Databank [18] under PDB entries 2HI4 [49] and ML1. The substrate and crystal water molecules were removed from the 2HI4 pdb file in Chimera UCSF [100] and chain A was selected. The heme was manually modified into a Cpd I structure with an Fe−O bond length set to 1.686 Å. Hydrogen atoms were added to the structure in Ambertools using pH 7 conditions [101]. Protonation states of key residues were manually corrected through visual inspection of their local environment. The melatonin structure was geometry optimized in Gaussian-09 [102] using a density functional theory method at the B3LYP/6-311 + G* level of theory [103,104] and converted into PDB format. The PDBs of our CYP1A2 and melatonin structures were assigned as receptor and ligand for substrate docking by AutoDock Vina [78] with a simulation box with a size of 20.0626 × 23.4102 × 21.855 Å^3^. The ten lowest-energy structures after docking were saved separately into PDB format as the orientation of the substrate with respect to the heme was different in each of them. The maximum energy difference between the best and worst binding mode was set to 2 kcal mol^−1^. The maximum number of binding modes was set to ten.

MD parameters for the heme complexes were calculated from QM methods by taking the heme complex with its first-coordination sphere ligands, namely the four ligands of the heme, one ligand of cysteine on the L-helix and the distal oxo ligand. The MCPB.py routine implemented in AmberTools 2018 [105] was used to generate the additional parameters for the MD simulations. The enzyme model was solvated in a rectangular box with a 10 Å distance between the box edges and the enzyme and filled with TIP3P defined water molecules [106], while the standard amino acids were described by the ff14SB12 forcefield [107]. The system was neutralized by adding Na^+^ and Cl^−^ ions to the surface of the model. After that, the prepared structure was minimized, heating to 310 K and finally a production run was performed. The minimization was performed in a single step without any constraints with steepest descent of 2000 cycles. Next, the enzyme was heated up from 0 to 310 K in 10 ns. Lastly, the production was run for 100 ns under the following conditions: constant temperate and pressure at 310 K and 1 bar, respectively. The 100 ns MD simulation was run sequentially for 20 times of 5 ns at a time. For model **VI** of CYP1A2 reactants and model **III** of CYP1A1 [65], we expanded the MD simulation to 1 μs.

The results from the MD simulations were collected into a database and analyzed in detail. The stability of each model was evaluated by checking the Root Mean Square Deviation (RMSD) of the atom positions of the various groups in the model, including the water shell. Most MD runs stabilized their RMSD within 60 ns. Therefore, the results of each model after 60 ns were further analyzed. In particular, the ten structures with lowest total energy were taken and their RMSD compared to the starting structure. This process ensured the similarity of those ten structures. Next, all residues within a radius of 5 Å from melatonin substrate were listed. Due to the mobility of the substrate in the binding pocket, its interactions play an important role in its possible reactivity. As such, all ten models are different and show different protein–substrate interactions. The occurrence of the interaction between the substrate and the residues nearby were collected and compared. The result from this process highlights all residues that potentially can interact with the substrate.

For a number of MD snapshots, QM cluster models were created of up to 350 atoms in size that contain the heme, substrate and heme–substrate protein interactions. To this end, a PDB file from the MD simulation was taken and trimmed to the appropriate size and shape. To this end, amino acid side chains pointing out of the active site were truncated to Gly residues by replacement of the side chain with a hydrogen atom. These large QM cluster models are known to reproduce experimental rates and selectivities well [108,109,110]; however, the larger the model, the more calculation time is required. Initial geometry optimizations were run at the UB3LYP level of theory [103,104] in Gaussian-09 [102] and utilized a basis set designated BS1 with LANL2DZ with core potential on iron [111] and 6–31G* on C, H, O, N and S. Single-point calculations with basis set BS2 were performed to correct the energies, whereby a 6–311 + G* basis set was used on C, H, O, N and S. The effect of solvent was tested through single-point calculations at the same level of theory but with a dielectric constant mimicking chlorobenzene included. To test the effect of the basis set on iron, a subsequent set of single point calculations at basis set BS3 improved the iron basis set to cc-pVTZ [112], and solvent through the conductor-type polarized continuum model with dielectric constant mimicking chlorobenzene. The UB3LYP/BS1 approach was used for geometry optimizations, analytical frequency calculation and constrained geometry scans, while BS3 was used in order to obtain more accurate energy results. Each model was calculated in the doublet and quartet spin states with overall charge of −2. Free energies were calculated at a temperature of 298.15 K and 1 bar pressure, whereby vibrational frequencies and entropies are corrected using the quasi-harmonic approximation [113]. In general, quasi-harmonic corrections give the same trends as without the corrections, and do not change the order of the transition states. The quantum chemical calculations were validated with a range of methods and little effect on the structures and energetics was found when the computational method or basis set were changed [114,115,116]. Transition states were verified by analyzing the imaginary mode, and all had a single imaginary frequency for the correct transition.

## 5. Conclusions

In this work, a large-scale computational study on melatonin activation by CYP1A2 enzymes is presented. We initially took a CYP1A2 crystal structure and docked melatonin into the substrate-binding pocket, which gave ten low-energy conformations. All these ten conformations were subsequently subjected to a 100 ns MD simulation. These MD simulations show that the protein is highly rigid and rarely escapes the substrate-binding pocket. Moreover, very little movement of the substrate and protein is seen during the full set of MD simulations. We then analyzed the substrate orientation and position with respect to the plane of the heme, and find most orientations close to the I-helix that provides hydrogen-bonding interactions to the substrate. Subsequently, we created a DFT cluster model of 283 atoms that includes the heme, substrate and their direct environments that provide hydrogen bonding and electrostatic interactions. Using DFT approaches, the full reaction mechanism of melatonin hydroxylation at the C^6^ position and *O*-demethylation were calculated. The aromatic hydroxylation has a rate determining C-O bond formation barrier that forms a σ-complex. Thereafter, a proton shuttle from the substrate *ipso*-position to the heme and back to the oxo group gives phenol products via small barriers in a highly exothermic process. The alternative pathway is *O*-demethylation that starts from a rate-determining hydrogen atom abstraction, followed by a small barrier for OH rebound to form the corresponding alcohol. The final deformylation step happens in solution via assistance by a proton. A detailed comparison is made between CYP1A1 and CYP1A2 activation of melatonin and we propose that the differences in substrate-activation originate from differences in the second-coordination sphere that determine substrate binding and positioning.

## Figures and Tables

**Table 1:** Calculated barrier heights for methoxy hydrogen atom abstraction (**TS1**_HA_) and C^6^-electrophilic addition (**TS1**_C6_) for melatonin activation by Compound I models of CYP1A1 and CYP1A2 ^1^.

Structure	CYP1A1 ^2^	CYP1A1 ^2^	CYP1A2 ^3^
	Model II	Model I	
**TS1**_HA_	3.3 (6.7)	17.8 (22.3)	16.5 (18.8)
**TS1**_C6_	6.5 (12.0)	10.0 (13.7)	4.5 (8.3)

^1^ ∆E + ZPE data in kcal mol^−1^ with respect to ^2^ **Re**; quartet spin data in parenthesis. ^2^ Data from Ref. [65]. ^3^ This work.

## Data Availability

All data is available as a Supplementary Materials File and can be requested from the authors.

## Supplementary Materials

The following supporting information can be downloaded at: https://www.mdpi.com/article/10.3390/ijms24043651/s1. References [18,49,65,78,100,101,102,105,106,107,113,117,118,119,120] are cited in supplementary material file.
