# Supplementary material for: Melatonin Activation by Cytochrome P450 Isozymes: How Does CYP1A2 Compare to CYP1A1?
Source: Int J Mol Sci. 2023 Feb 11;24(4):3651. doi: 10.3390/ijms24043651 (PMC9959256; doi:10.3390/ijms24043651)
Supplement: Supplementary file 1 [file ijms-24-03651-s001.zip › ijms-2190131-supplementary.pdf]

Supporting information

Melatonin activation by Cytochrome P450 isozymes. How does CYP1A2 compare to CYP1A1?

Thirakorn Mokkawes [1,2], and Sam P. de Visser [1,2,*]

[1] Manchester Institute of Biotechnology, The University of Manchester, 131 Princess Street, Manchester M1 7DN, United Kingdom
[2] Department of Chemical Engineering, The University of Manchester, Oxford Road, Manchester M13 9PL, United Kingdom; sam.devisser@manchester.ac.uk

Table of Contents

1 Methodology 1
1.1 Primary model preparation 1
1.2 Substrate docking 1
1.3 Molecular dynamics simulation 2
1.4 Data extraction from molecular dynamics simulation 3
1.5 Data selection of molecular dynamics simulation 3
1.6 Modification of small model for quantum mechanics simulation 3
1.7 Quantum mechanics simulation 3
1.8 Chemical reaction determination of quantum mechanics simulation 4
2 Reference 5
3 Binding poses of CYP1A2 systems from molecular dynamics simulation 7
4 RMSD of CYP1A1 systems 13
5 RMSD matching for different CYP1A2 systems 15
6 Substrate location in the active sites 17
7 Amino acid distributions 19
8 Linear interaction energy of 10 reactant models of CYP1A2 20
9 Active site cluster of quantum mechanics simulation 20
10 CYP1A1 molecular dynamics Simulation in 1 μs 21
11 Model 6 Reactant of CYP1A2 molecular dynamics Simulation in 1 μs 22
12 Comparison of CYP1A1 and CYP1A2 from Molecular Dynamics Simulation 23
13 Scanning from quantum mechanics calculation 30
14 Energy data from quantum mechanics calculation 31
15 Energy data from quantum mechanics calculation with RRHO fixed 33
16 Group charges and spin densities from quantum mechanics simulation 35
17 Imaginary frequency data from quantum mechanics simulation 37
18 Geometry data from quantum mechanics simulation 37

1 Methodology

1.1 Primary model preparation

Here is the outline of the steps involved in preparing a structure for a molecular dynamics simulation as starting from a protein databank (PDB) file:

1. Select the PDB file: The first step was to select the enzyme for this study by fetching the data from rcsb.org[1]. The enzyme in this study was CYP1A2. We selected the chain A of 2HI4[2] PDB structure which was the latest released structure and with good resolution.
2. Clean the PDB file: The next step was to remove unnecessary residues (solvent, glycerol, ions) and/or ligands and ensuring that all residues were properly named and had their correct residue types (ATOM or HETATM). This was done using a text editor software and Chimera UCSF.
3. Add missing atoms: In this step, the heme-iron complex was modified to a Compound I (Cpd I) structure. The additional oxygen atom was added over the central iron atom with bond length 1.686 Å, which was a distance taken from a previous study using the quantum mechanics method[3]. This was customized using Chimera UCSF[4].
4. Add hydrogen atoms: Once all of the missing atoms were corrected and presented in the PDB file, the hydrogen atoms were added using AmberTools 2018[5].
5. Prepare the substrate: Melatonin was the substrate of this study. It was fetched from the ML1[6] PDB and reoptimized using a quantum mechanics method in Gaussian version 16[7]. The optimized structure was converted to a PDB file by the Antechamber[8] software package provided by AmberTools 2018.

1.2 Substrate docking

Here is the outline of the steps involved in docking a substrate into the active site of the PDB file:

1. Prepare the receptor: The first step was to prepare the receptor, which was the well-prepared structure from section 1.1.
2. Prepare the ligand: The next step was to prepare the ligand, which was the small molecule that will be docked into the receptor, namely melatonin.
3. Select the docking software: In this study, we used AutoDock Vina[9] which uses a generic algorithm to search for the lowest energy configuration of a small molecule in the active site of a protein.
4. Set up the docking simulation: Once the receptor and ligand have been prepared, the next step was to set up the docking simulation. The size of the simulation box was 20.0626, 23.4102, 21.855 with the center coordinate at 5.74371, 17.7915, 17.8511. The box was placed at the center of the enzyme active site containing CpdI, the I helix, the G helix and the B' helix. The maximum energy difference between each search was set to 2 kcal/mol. The maximum number of binding modes was set to 10. This resulted in 10 binding poses which were further and used for the molecular dynamics simulations.
5. Save different docked substrate poses: The ten low-energy binding poses of melatonin were separately saved as PDB files.

1.3 Molecular dynamics simulation

Here is the outline of the molecular dynamics simulations of the heme-iron complexes:

1. Prepare the mol2 files for non-standard molecules: Amber needs the information about non-standard chemical compounds which was in the mol2 file format. There are atom names, atom types, bond types, bond orders, etc. This was done using Antechamber.
2. Prepare the frcmod files for non-standard molecules: The forcefields for non-standard molecules were not available in Amber. This information can be prepared by converting data from mol2 file using parmchk2 software package provided in AmberTools 2018.
3. Generate forcefield for heme-iron complex: The atoms within 2.5 Å around the central iron atom in the porphyrin are optimized by quantum mechanics and the output was transferred to forcefield parameter. This was generated by MCPB.py[10] that was the python-based software package provided on Amber.
4. Solvate the protein: Once the force field for non-standard residues were generated, the whole system was solvated in water using the TIP3P[11] forcefield while the standard amino acids were performed by ff14SB[12] forcefield. The system was neutralized by Na^+ and Cl^-. The rectangular solvate-box was set to a size of 10 Å, which was the distance between the box edges and the protein. This can be done using Amber's tleap software package.
5. Minimize the system: Once the system was solvated in water and neutralized, it was minimized to remove any bad contacts between the molecule and solvent. The steepest descent was set to 2000 with a cutoff set to 10 Å.
6. Heat the system: In this step, the system is heated up to bring the system into a more stable state. The system is heated up to a temperature of 310 K during a 10 ns simulation.
7. Run the MD production: Finally, the system was run for 100 ns for the general study and 1000 ns for the comparison of CYP1A1 and CYP1A2. It was run under the constant temperature and pressure condition at 310 K and 1 bar by running through multiple loops of 5 ns.
8. The calculations were run on different intermediates along the mechanisms for *O*-demethylation and aromatic hydroxylation. The labels of each data were presented in the following figure:

$$A_C^B$$

   Where:
   - A is the intermediate created, including reactant (Re), intermediate 1 (IM1), intermediate 2 (IM2), product (P).
   - B is the reaction pathway, including *O*-demethylation (ODE) or aromatic hydroxylation (HYD).
   - C is the model number as taken from the MD simulations that ranges from 1 to 10.

1.4 Data extraction from molecular dynamics simulation

Once the molecular dynamics simulation was finished, all results from multiple loops were combined using the cpptraj software package provided on Amber and pytraj[13] which was python-based library. The data was in csv format and illustrated by the python libraries named plotly and seaborn. For the geometry visualization, it was presented by Chimera UCSF.

1.5 Data selection of molecular dynamics simulation

Here is the outline of the data selection from the molecular dynamics simulations:

1. Compare the structure at the start and end of the molecular dynamics simulation: Focus on the protein changes and substrate-binding orientations of each system in the snapshots between 1 ns and 100 ns. The results are shown in Figure S1 to S6.
2. Determine the RMSD: The RMSD of each structure was extracted to see the stability of protein, substrate and heme in the molecular dynamics simulations. The results are shown in Figure S7 and S8.
3. Compare the RMSD from different intermediates: Each structure from 90 ns to 100 ns was used as the reference structure to calculate the RMSD of the next systems. For example, the snapshot at 90 ns of reactant was used to calculate the RMSD of 10 models from 1 ns to 100 ns. The RMSD of the comparison from 90 ns to 100 ns were presented in median values. The structure that mostly gave low RMSD was selected as the reference structure for quantum mechanics calculations. That structure was the model 6 of reactant. The results are shown in Figure S9 and S10.
4. Map the substrate in the active site: The substrate orientations were mapped into the quadrants based on the heme plane by measuring the C6 position of the substrate using the pyramid method. From the previous step, the snapshot of the selected reactant at the median that had highest density on x and y axis was selected. The selected snapshot of model 6 reactant was 80.4 ns. The results are shown in Figure S11 and S12.

1.6 Modification of small model for quantum mechanics simulation

The amino acids within 5 Å of the melatonin are reported in Figure S13 with the distributions of amino acids. Those residues are included in the active site cluster model for which quantum mechanics calculation were performed. However, some residues or side chains had to be truncated because of the size limitation of the quantum mechanics calculation. The truncated model had 283 atoms. The result is shown in Figure S14.

1.7 Quantum mechanics simulation

The quantum mechanics calculation was performed with Gaussian version 16 by the following steps:

1. Optimize cluster models and local minima: The modified small model was optimized with basis set 1 (BS1) and the UB3LYP density functional approach. The iron (Fe) atom was described with the LANL2DZ+ECP basis set and the rest of the atoms employed 6-31G*. Calculations were performed at 298.15 K (25°C) in the doublet and quartet spin multiplicities and overall charge -2.
2. Geometry scans for describing specific chemical reaction pathways: The atoms involved in the *O*-demethylation and aromatic hydroxylation were moved closer to the oxo group by constraint geometry scans were the O-H/C-O distances

were shortened in a stepwise manner. Each step had fixed O-H/C-O distance and applied a full geometry optimization. During this process the electronic energy changed and followed past a maximum and leading to another local minimum. The scan was run until the next local minimum was reached using the computational methods as described in (1).

3. Optimize transition state: The structure at the maximum energy point was reoptimized as a transition state and with basis set BS1 with the same conditions as in (1).
4. Perform better basis set calculations: The optimized structures were subjected to the single point calculation with basis set 2 (BS2) and the UB3LYP density functional. The iron (Fe) atom was described with cc-pVTZ and the rest of the atoms with a 6-311++G* basis set. Energies were calculated at 298.15 K (25°C) in the doublet and quartet spin multiplicities and with overall charge -2.
5. Generate energy data: The optimized structures were confirmed as local minima or first-order saddle points with a frequency calculation with BS1 under the same condition in (1) resulting in energy data including zero-point energy, electronic energy, free energy, etc.
6. The vibrational frequencies obtained from Gaussian were corrected with the Goodvibes[15] software package. The calculated energy was added by the approximation for the quasi-harmonic enthalpy correction. It replaced harmonic energy contributions with a quasi-RRHO vibrational energy term via the Head-Gordon expression at the 298.15 K and 1 atm.

1.8 Chemical reaction determination of quantum mechanics simulation

Here is the outline of the chemical reactions determined by quantum mechanics calculations:

1. Extract energy data: The thermochemistry data was extracted and includes zero-point energy, electronic energy, electronic energy with zero-point energy and free energy. The chemical reaction energies were determined from relative energies which were derived from absolute free energies.
2. Illustrate energy profile: To give the insight of chemical reaction, the energy profile was created from the relative energies from BS2 single point calculation using Chemdraw[14] software.
3. Extract charges and spin densities: The electron configurations and orbitals were determined by charges and spin densities.
4. Extract geometry data: Geometry information presented the positions of the atoms relative to doublet and quartet multiplicities. The geometry data including bond lengths, angles and dihedrals were extracted by a python-based library.
5. Extract imaginary frequencies: The imaginary frequency relates to the activation energy that is required to overcome the energy barrier and initiate a chemical reaction. It was extracted by data mining using python.

2 Reference

1. Berman, H. M.; Westbrook, J.; Feng, Z.; Gilliland, G.; Bhat, T. N.; Weissig, H.; Shindyalov, I. N.; Bourne, P. E. The Protein Data Bank. *Nucleic Acids Research* **2000,** *28* (1), 235-242.
2. Sansen, S.; Yano, J. K.; Reynald, R. L.; Schoch, G. A.; Griffin, K. J.; Stout, C. D.; Johnson, E. F. Adaptations for the oxidation of polycyclic aromatic hydrocarbons exhibited by the structure of human P450 1A2. *J Biol Chem* **2007,** *282* (19), 14348-55.
3. Mokkawes, T.; Lim, Z. Q.; de Visser, S. P. Mechanism of Melatonin Metabolism by CYP1A1: What Determines the Bifurcation Pathways of Hydroxylation versus Deformylation? *The Journal of Physical Chemistry B* **2022,** *126* (46), 9591-9606.
4. Pettersen, E. F.; Goddard, T. D.; Huang, C. C.; Couch, G. S.; Greenblatt, D. M.; Meng, E. C.; Ferrin, T. E. UCSF Chimera--a visualization system for exploratory research and analysis. *J Comput Chem* **2004,** *25* (13), 1605-1612.
5. Case, D. A.; Cheatham, T. E., 3rd; Darden, T.; Gohlke, H.; Luo, R.; Merz, K. M., Jr.; Onufriev, A.; Simmerling, C.; Wang, B.; Woods, R. J. The Amber biomolecular simulation programs. *J Comput Chem* **2005,** *26* (16), 1668-88.
6. Sliwiak, J.; Sikorski, M.; Jaskolski, M. PR-10 proteins as potential mediators of melatonin-cytokinin cross-talk in plants: crystallographic studies of LlPR-10.2B isoform from yellow lupine. *The FEBS Journal* [https://doi.org/10.1111/febs.14455] **2018,** *285* (10), 1907-1922.
7. Frisch, M. J.; Trucks, G. W.; Schlegel, H. B.; Scuseria, G. E.; Robb, M. A.; Cheeseman, J. R.; Scalmani, G.; Barone, V.; Petersson, G. A.; Nakatsuji, H.; Li, X.; Caricato, M.; Marenich, A. V.; Bloino, J.; Janesko, B. G.; Gomperts, R.; Mennucci, B.; Hratchian, H. P.; Ortiz, J. V.; Izmaylov, A. F.; Sonnenberg, J. L.; Williams; Ding, F.; Lipparini, F.; Egidi, F.; Goings, J.; Peng, B.; Petrone, A.; Henderson, T.; Ranasinghe, D.; Zakrzewski, V. G.; Gao, J.; Rega, N.; Zheng, G.; Liang, W.; Hada, M.; Ehara, M.; Toyota, K.; Fukuda, R.; Hasegawa, J.; Ishida, M.; Nakajima, T.; Honda, Y.; Kitao, O.; Nakai, H.; Vreven, T.; Throssell, K.; Montgomery Jr., J. A.; Peralta, J. E.; Ogliaro, F.; Bearpark, M. J.; Heyd, J. J.; Brothers, E. N.; Kudin, K. N.; Staroverov, V. N.; Keith, T. A.; Kobayashi, R.; Normand, J.; Raghavachari, K.; Rendell, A. P.; Burant, J. C.; Iyengar, S. S.; Tomasi, J.; Cossi, M.; Millam, J. M.; Klene, M.; Adamo, C.; Cammi, R.; Ochterski, J. W.; Martin, R. L.; Morokuma, K.; Farkas, O.; Foresman, J. B.; Fox, D. J. *Gaussian 16 Rev. C.01*, Wallingford, CT, 2016.
8. Wang, J.; Wang, W.; Kollman, P.; Case, D. ANTECHAMBER: an accessory software package for molecular mechanical calculations. *Journal of Chemical Information and Computer Sciences - JCISD* **2000,** *222*.
9. Trott, O.; Olson, A. J. AutoDock Vina: improving the speed and accuracy of docking with a new scoring function, efficient optimization, and multithreading. *J Comput Chem* **2010,** *31* (2), 455-61.
10. Li, P.; Merz, K. M., Jr. MCPB.py: A Python Based Metal Center Parameter Builder. *Journal of Chemical Information and Modeling* **2016,** *56* (4), 599-604.
11. Price, D. J.; Brooks, C. L. A modified TIP3P water potential for simulation with Ewald summation. *The Journal of Chemical Physics* **2004,** *121* (20), 10096-10103.
12. Maier, J. A.; Martinez, C.; Kasavajhala, K.; Wickstrom, L.; Hauser, K. E.; Simmerling, C. ff14SB: Improving the Accuracy of Protein Side Chain and Backbone Parameters from ff99SB.

Journal of Chemical Theory and Computation **2015,** *11* (8), 3696-3713.
13. Roe, D. R.; Cheatham, T. E., III. PTRAJ and CPPTRAJ: Software for Processing and Analysis of Molecular Dynamics Trajectory Data. *Journal of Chemical Theory and Computation* **2013,** *9* (7), 3084-3095.
14. ChemDraw professional.
15. Luchini, G.; Alegre-Requena, J. V.; Funes-Ardoiz, I.; Paton, R. S. F1000Research, 2020, 9, 291.
GoodVibes version 2.0.3 DOI: 10.5281/zenodo.1435820.

3 Binding poses of CYP1A2 systems from molecular dynamics simulation

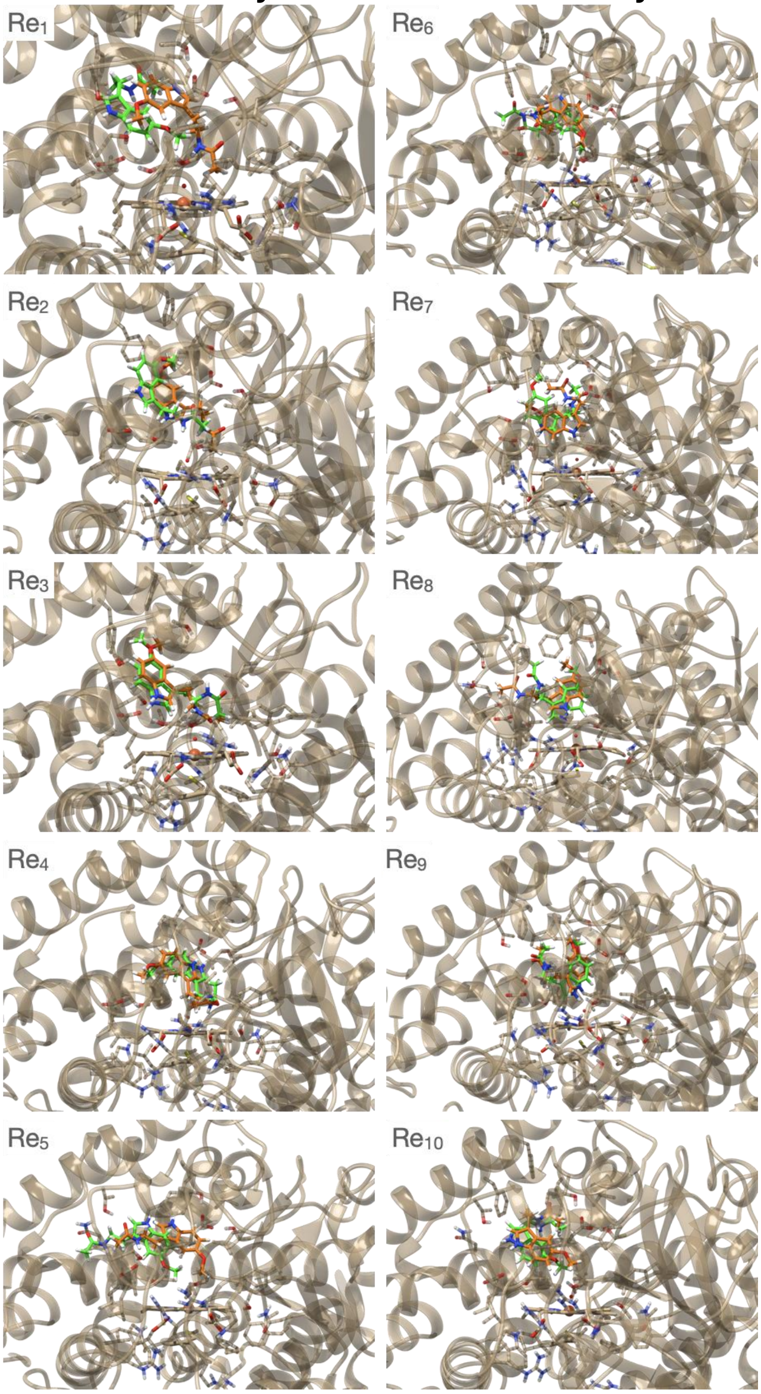

Figure S1 Binding poses of the reactant complex as obtained from 10 MD simulations of 10 different starting orientations of substrate. The substrate orientation structures at 1 and 100 ns are presented in orange and green.

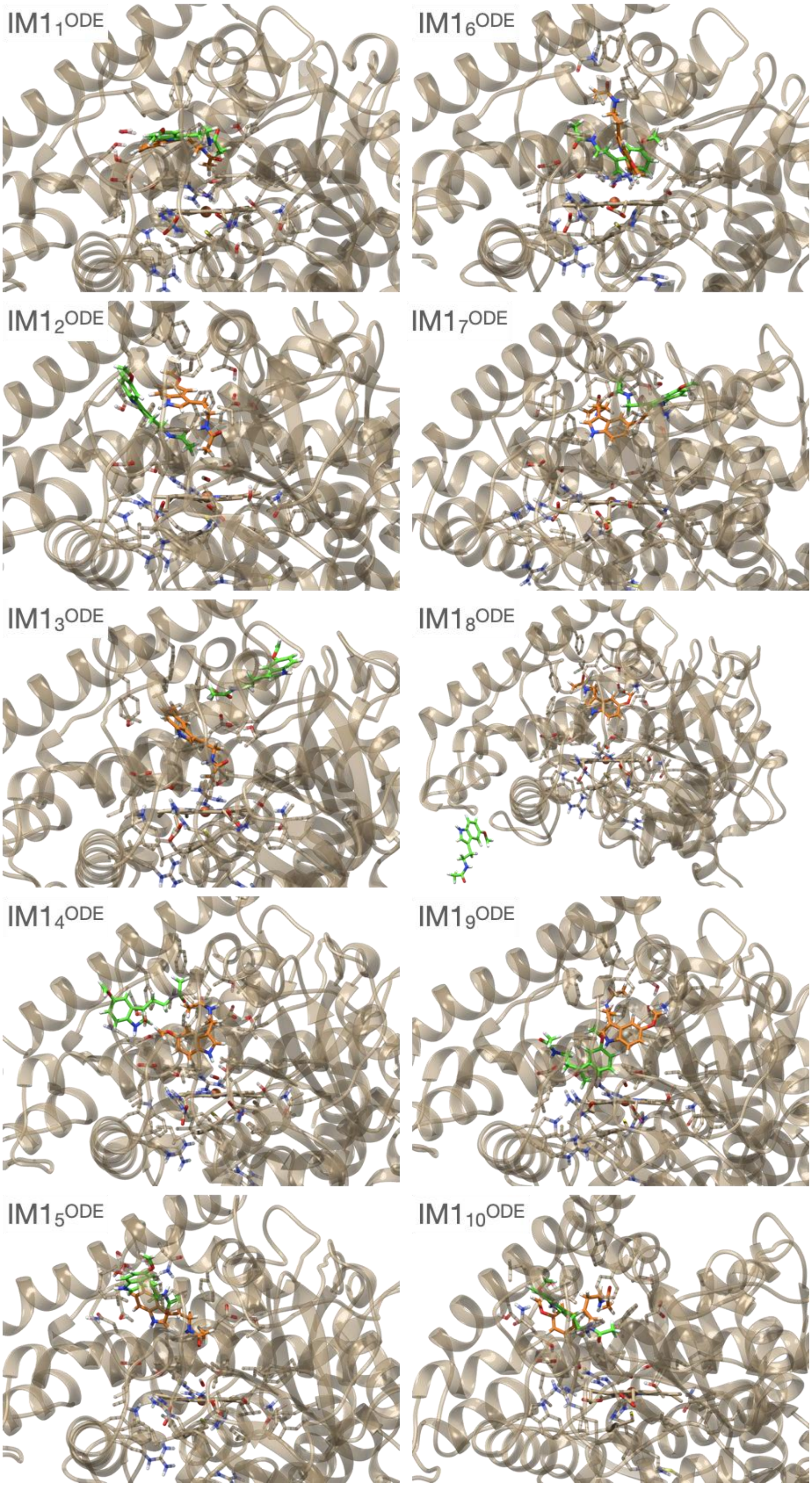

Figure S2 Binding poses of the substrate in intermediate-1 along the *O*-demethylation pathway in 10 models as obtained from MD simulations. Structures at 1 and 100 ns are presented in orange and green.

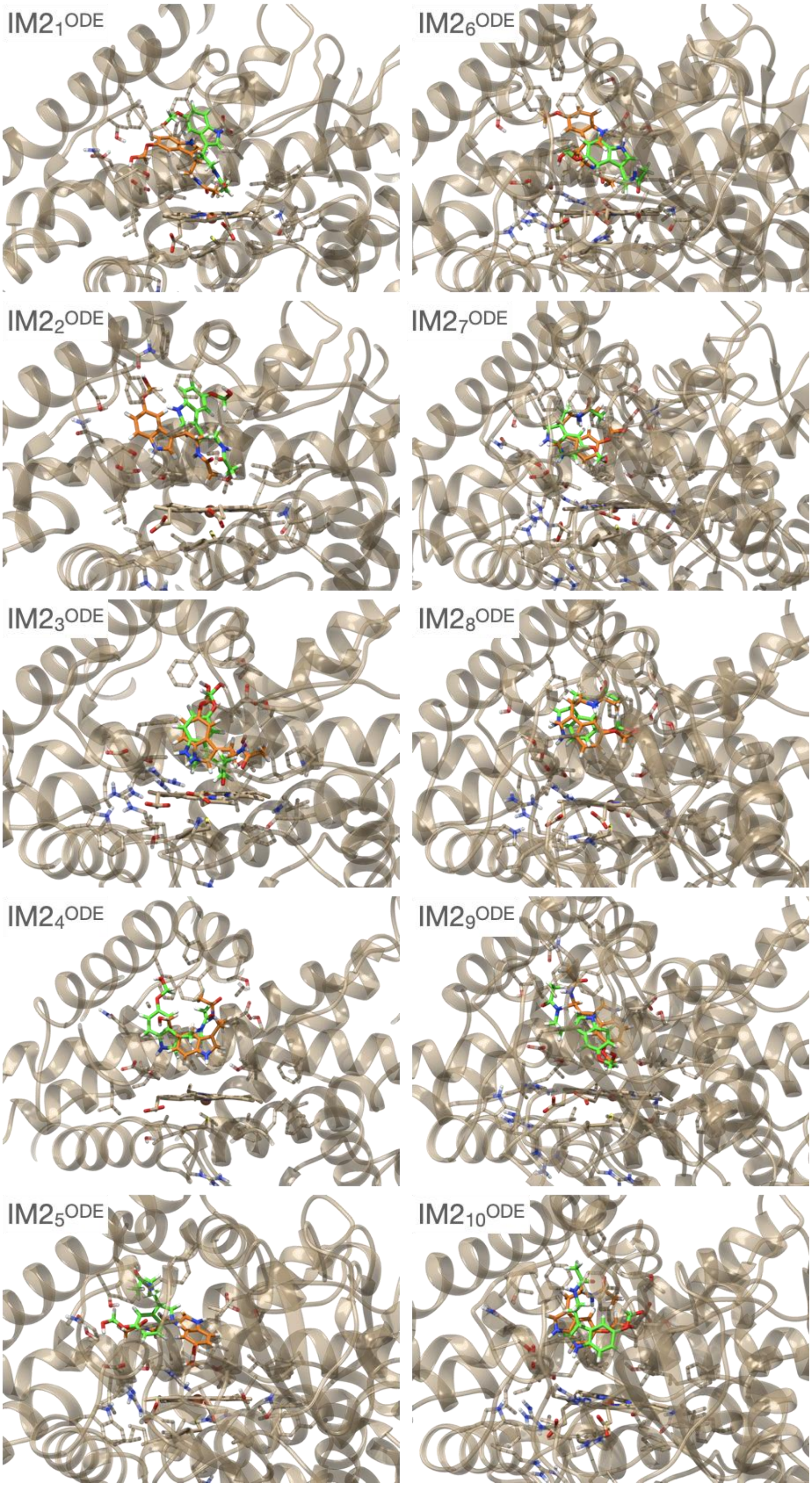

Figure S3 Binding poses of the substrate in intermediate-2 along the *O*-demethylation pathway in 10 models as obtained from MD simulations. Structures at 1 and 100 ns are presented in orange and green.

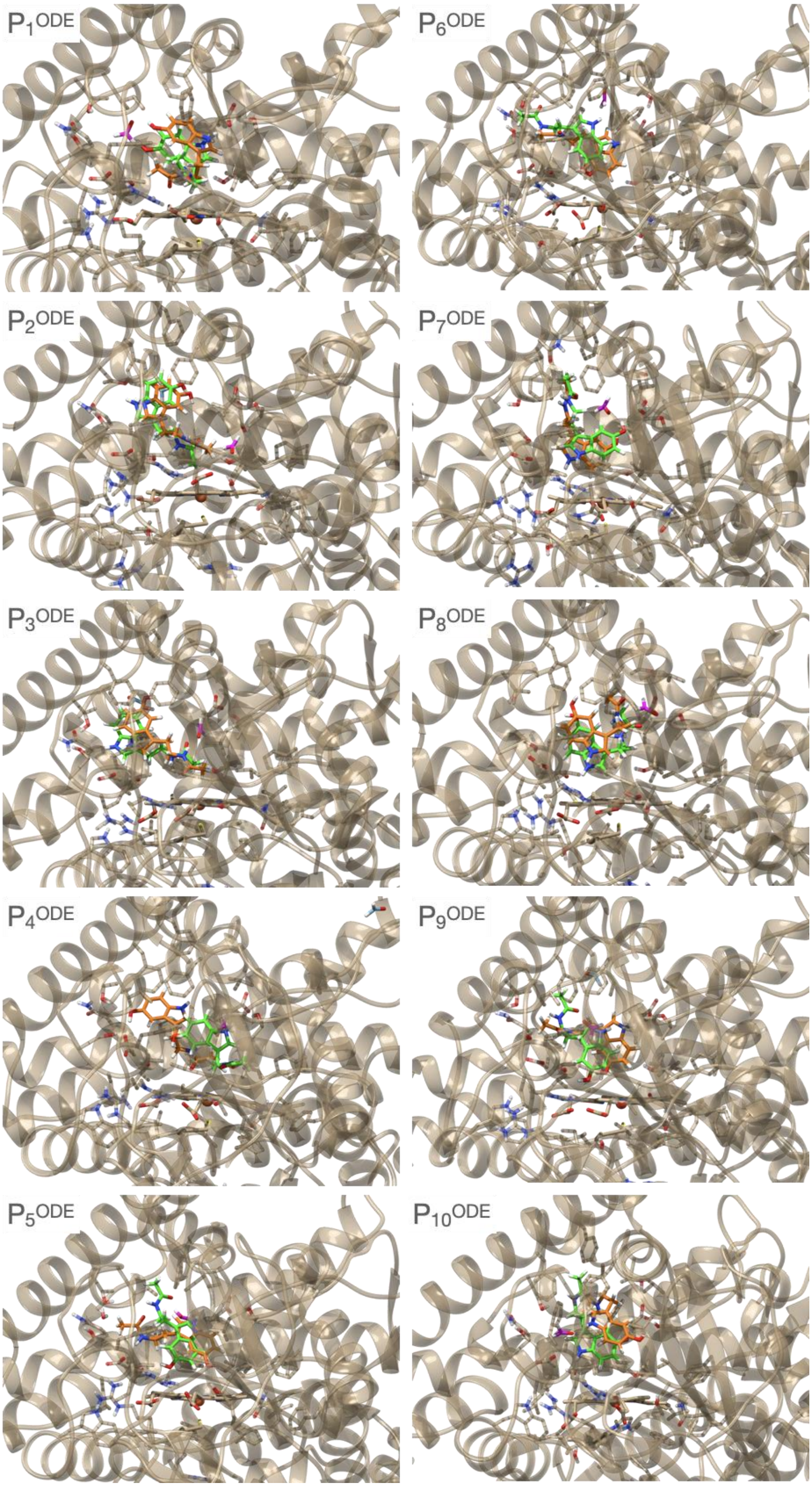

Figure S4 Binding poses of the substrate in product along the *O*-demethylation pathway in 10 models as obtained from MD simulations. Structures at 1 and 100 ns are presented in orange and green.

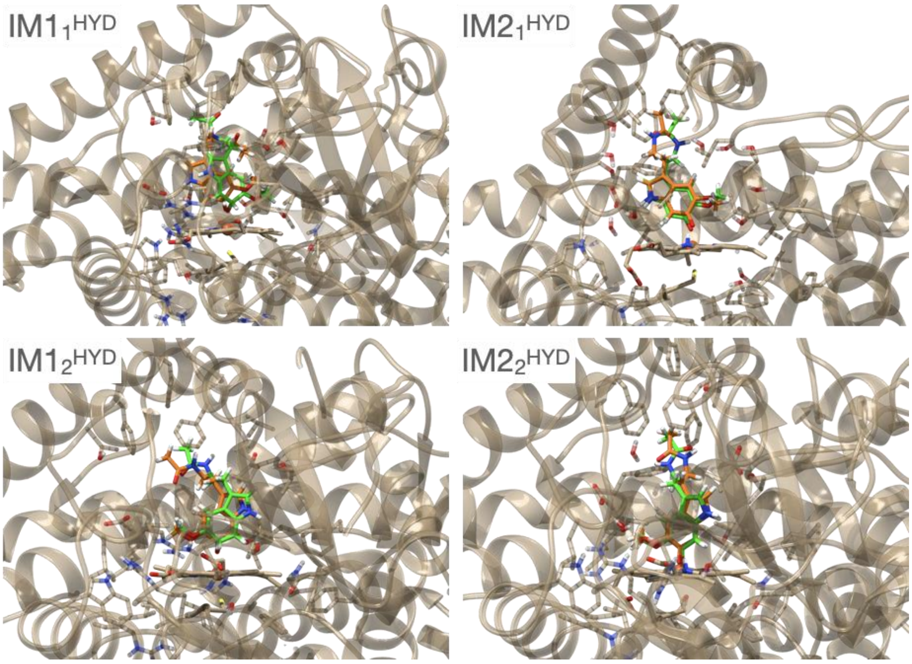

Figure S5 Binding poses of the substrate in intermediate-1 and intermediate-2 along the aromatic hydroxylation pathway in 2 models as obtained from MD simulations. Structures at 1 and 100 ns are presented in orange and green.

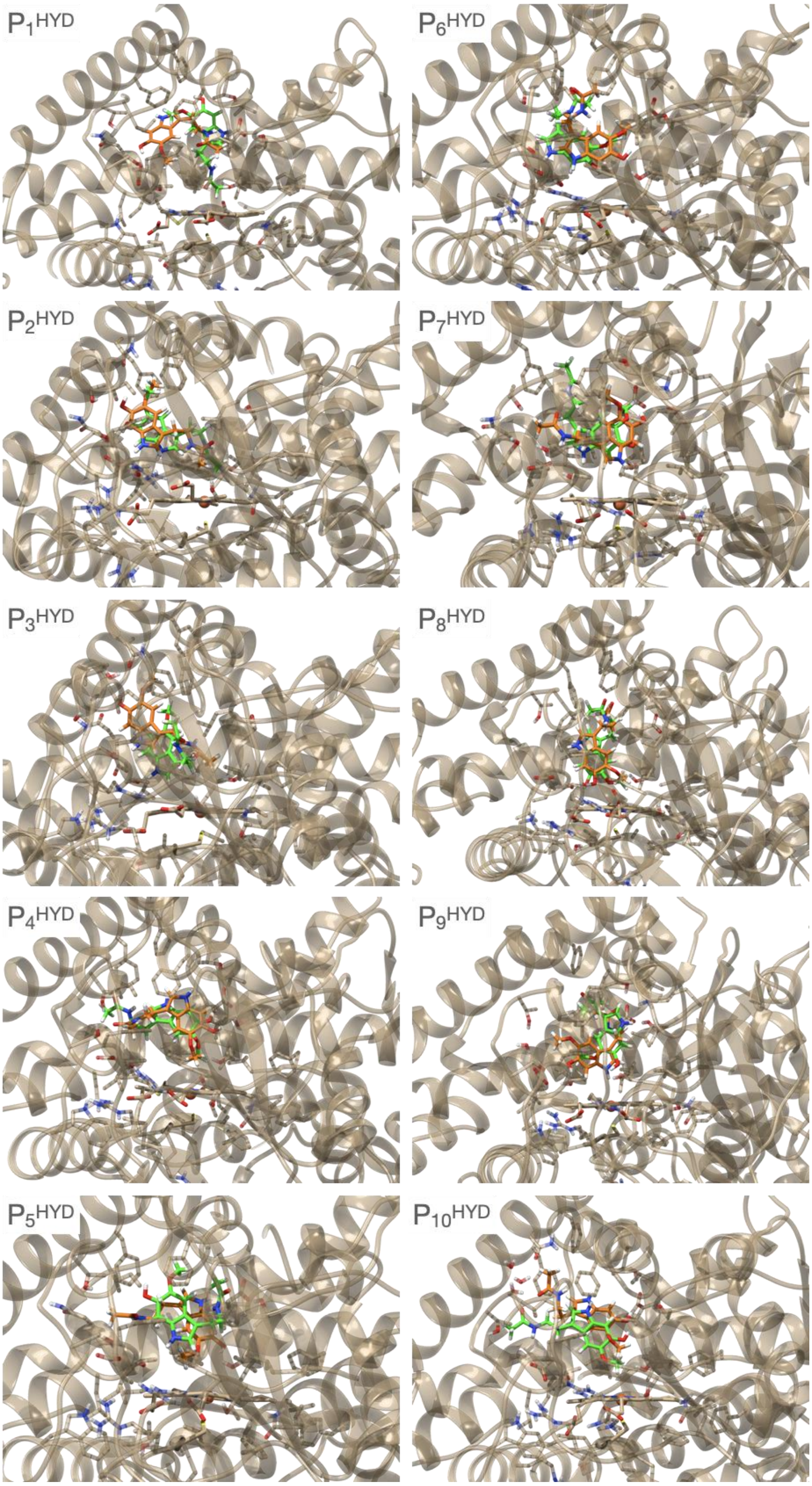

Figure S6 Binding poses of the substrate in product along the aromatic hydroxylation pathway in 10 models as obtained from MD simulations. Structures at 1 and 100 ns are presented in orange and green.

4 RMSD of CYP1A1 systems

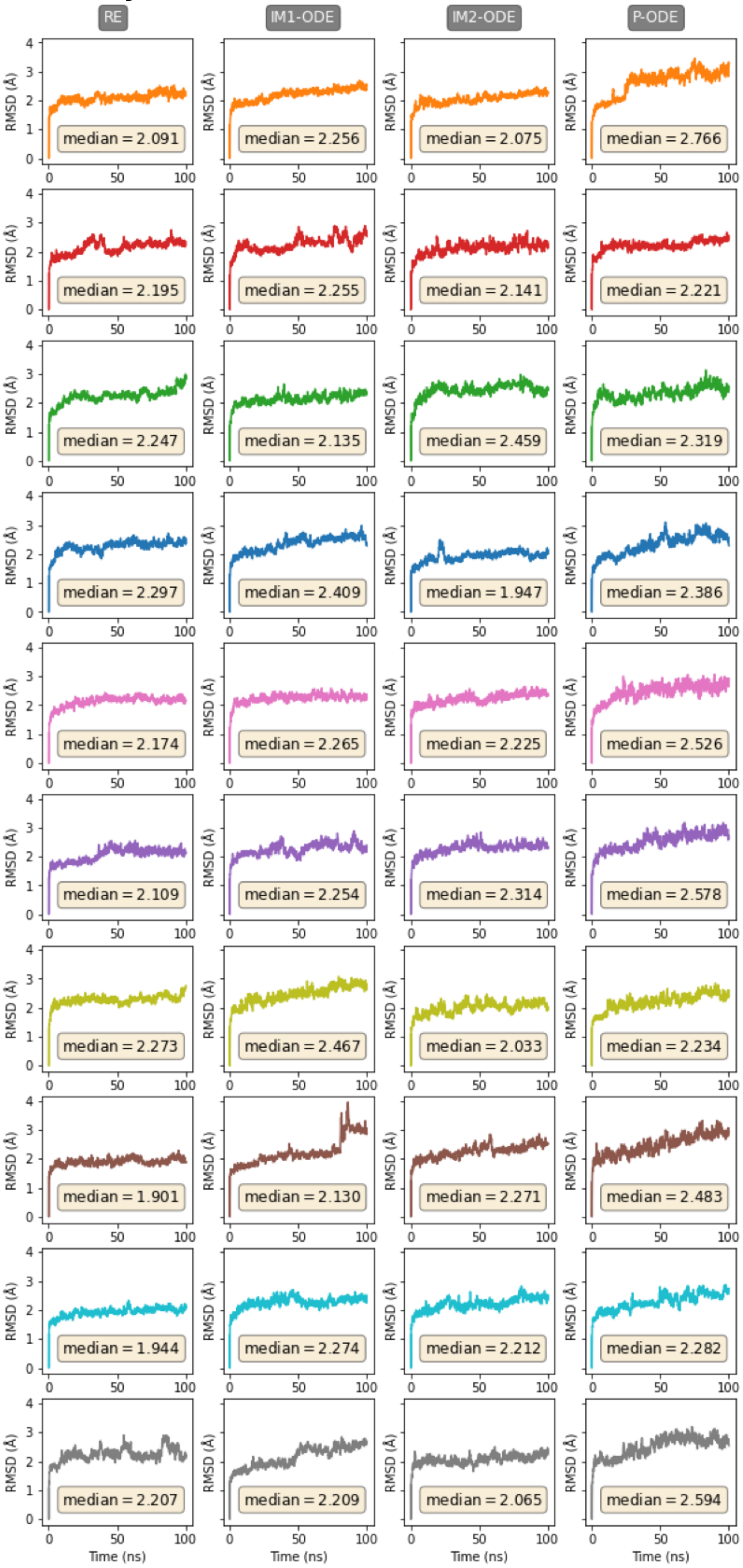

Figure S7 RMSD values of the protein for the MD simulations of various intermediates along the pathway for *O*-demethylation in 100 ns.

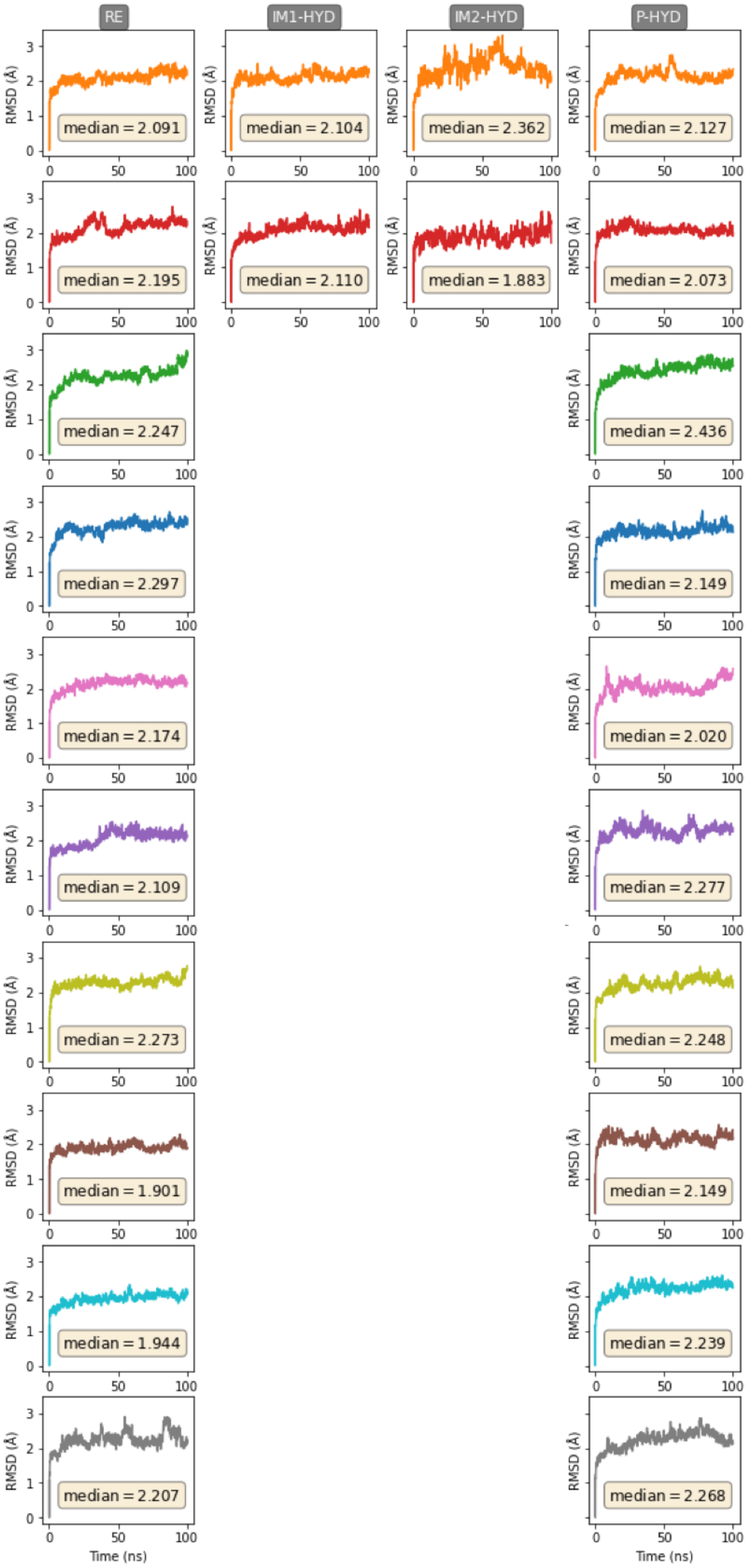

Figure S8 RMSD values of the protein for the MD simulations of various intermediates along the pathway for aromatic hydroxylation in 100 ns.

5 RMSD matching for different CYP1A2 systems

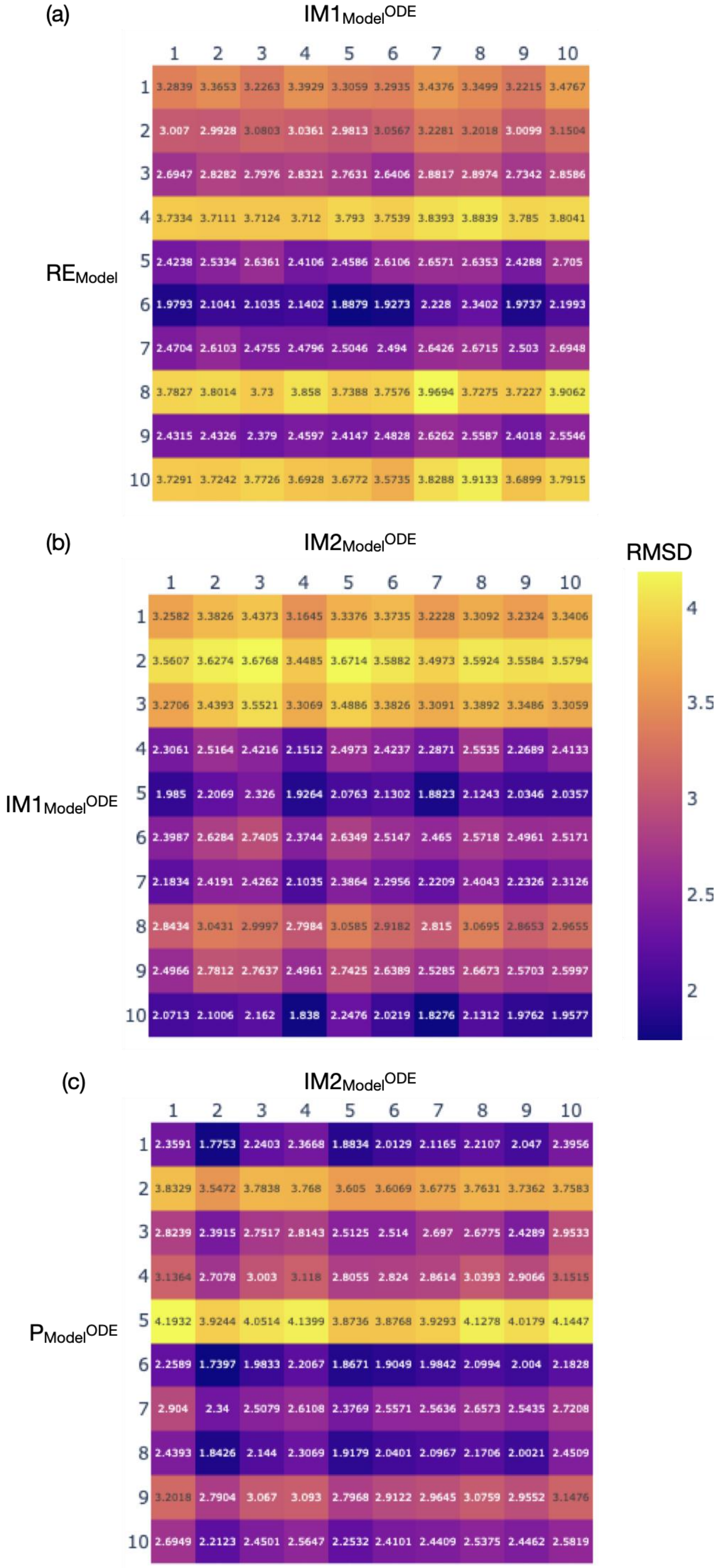

Figure S9 RMSD comparison of intermediate states for *O*-demethylation during a 100 ns MD simulation.

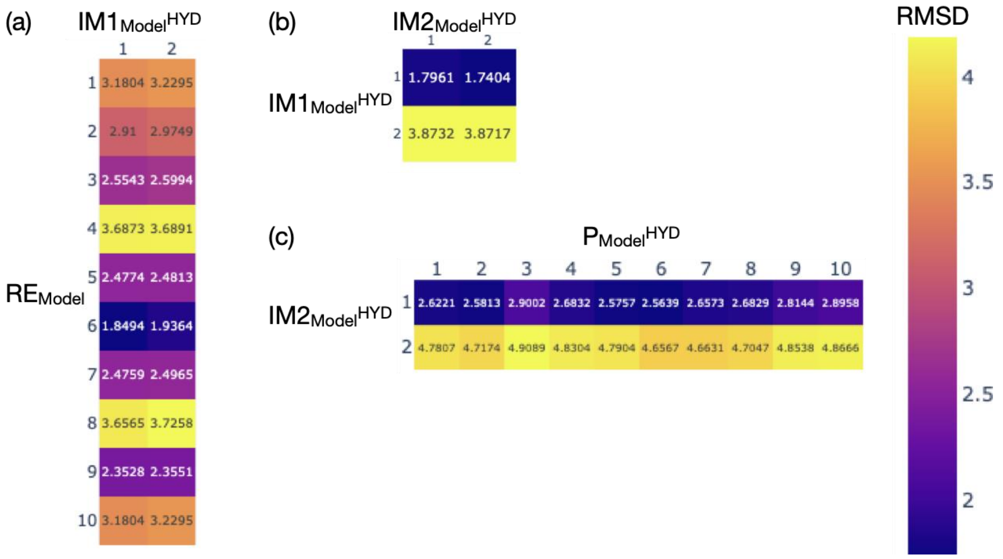

Figure S10 RMSD comparison of intermediate states for aromatic hydroxylation during a 100 ns MD simulation.

6 Substrate location in the active sites

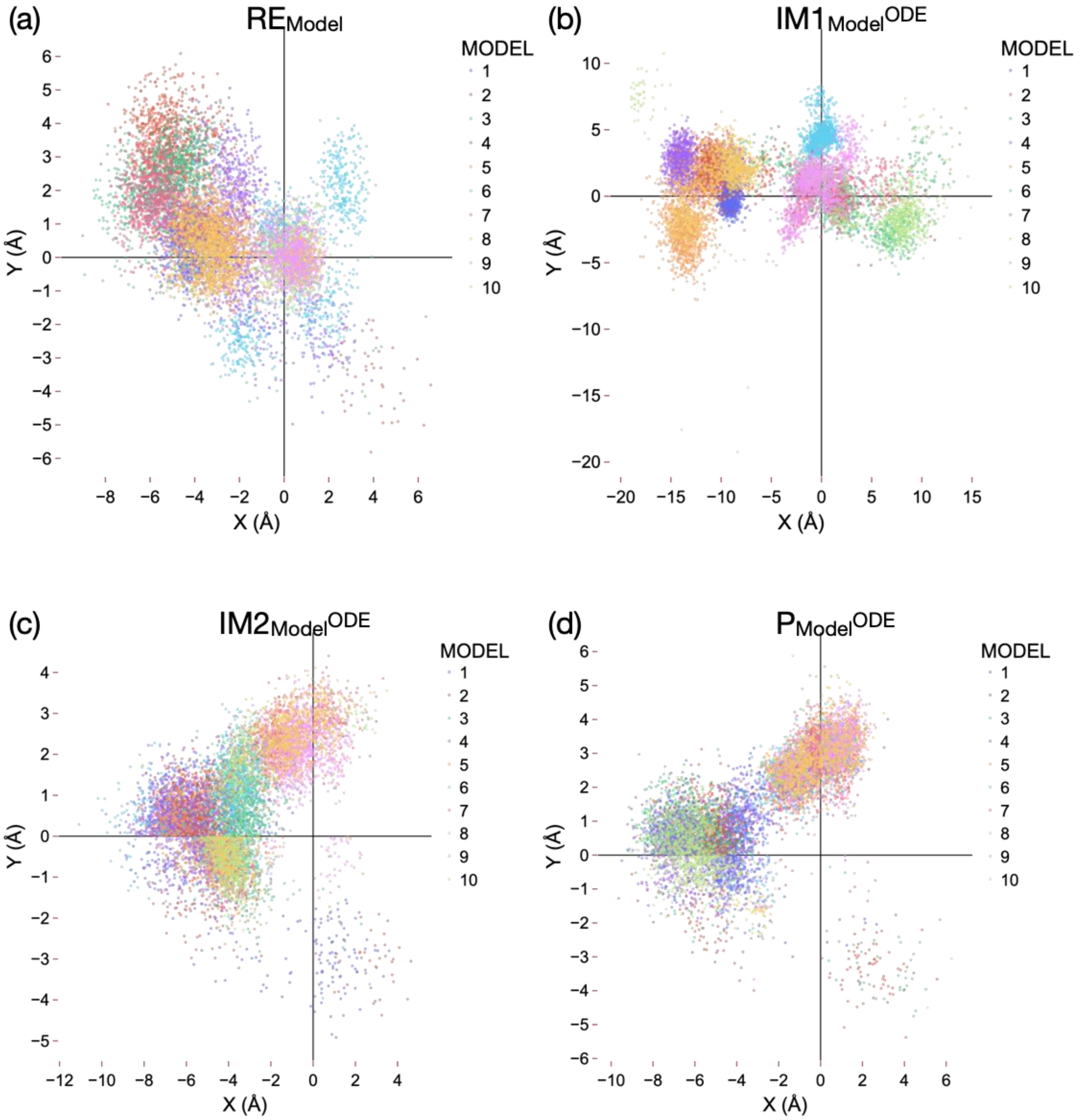

Figure S11 Substrate mapping in the active site in intermediates along the mechanism for *O*-demethylation as obtained from a 100 ns MD simulation.

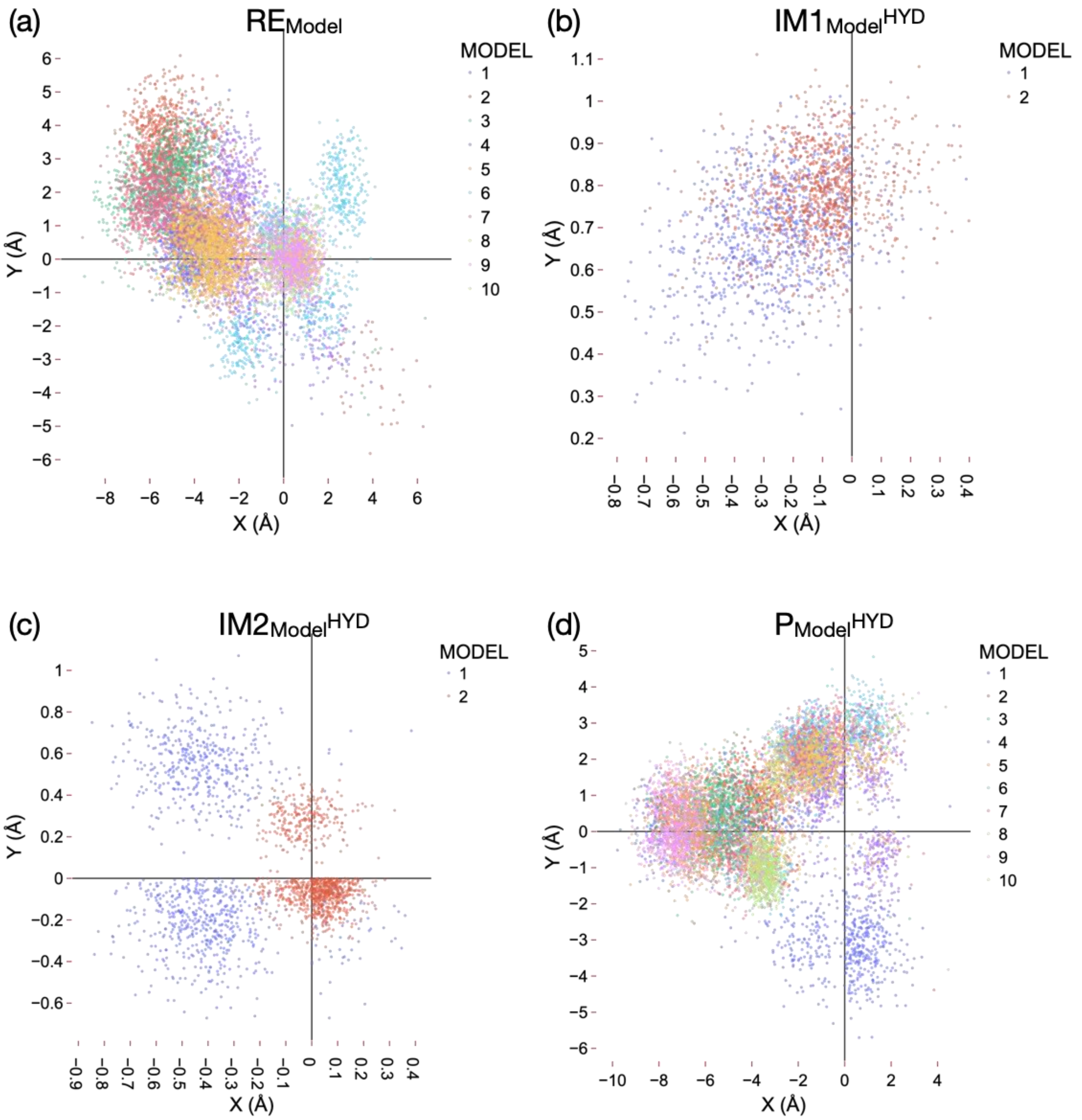

Figure S12 Substrate mapping in the active site in intermediates along the mechanism for aromatic hydroxylation as obtained from a 100 ns MD simulation.

7 Amino acid distributions

(a)

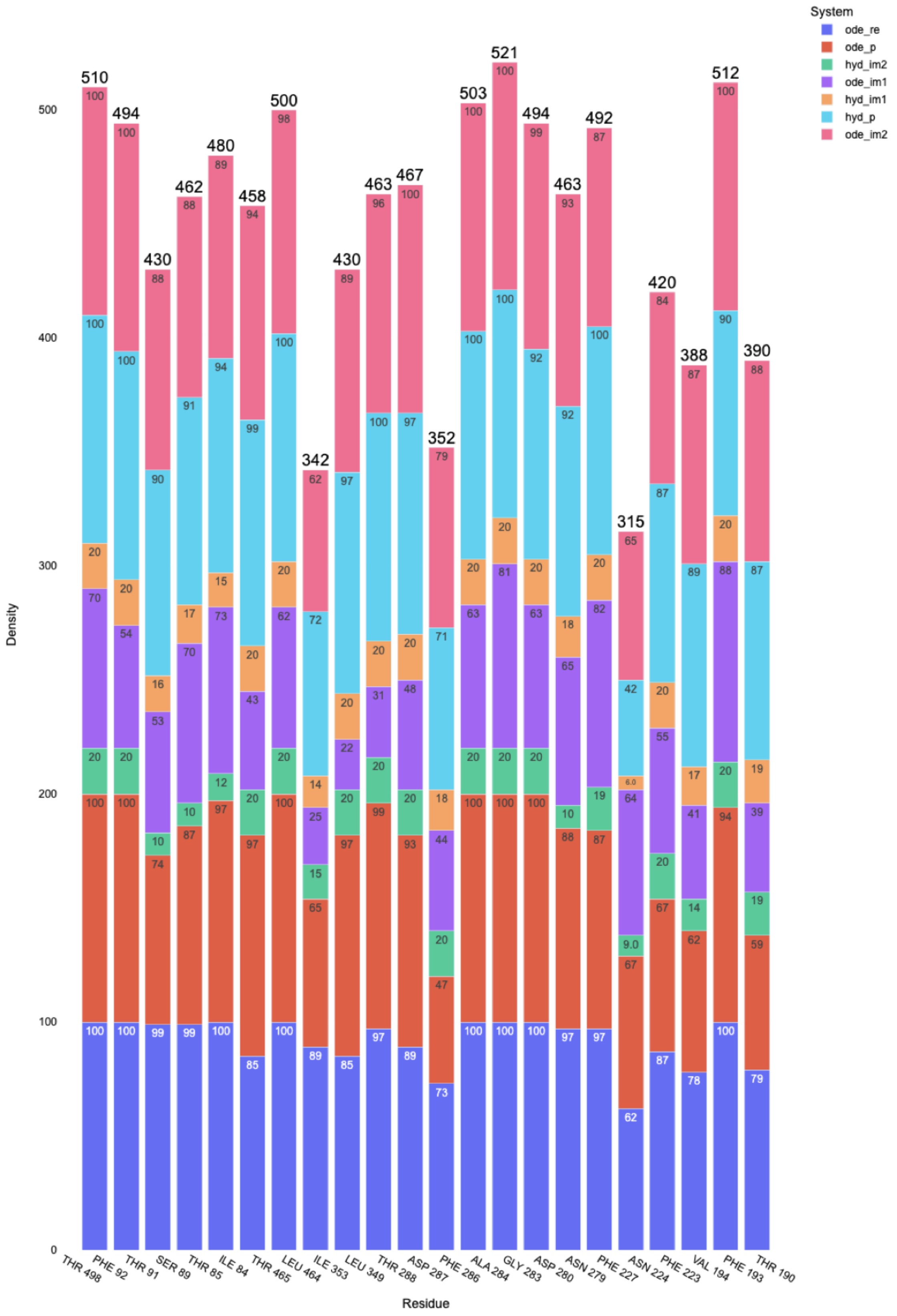

Figure S13 Amino acid distributions within 5 Å around the melatonin as derived from snapshot structures from MD simulations.

8 Linear interaction energy of 10 reactant models of CYP1A2

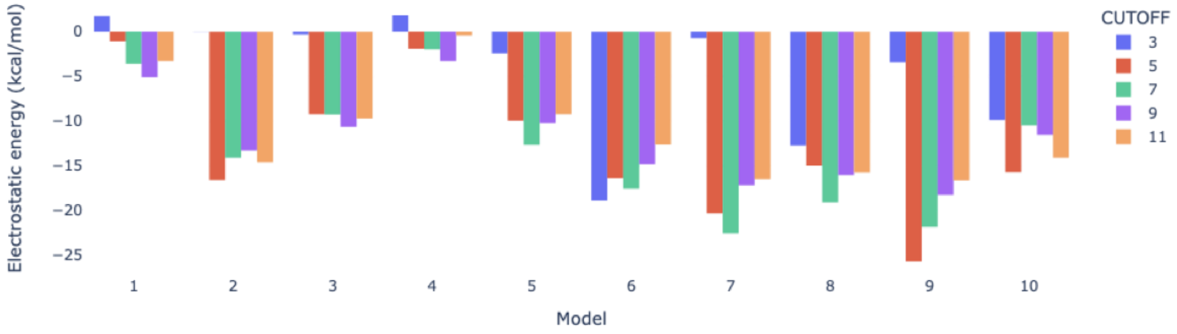

Figure S14 Linear interaction energy of the 10 reactant models as obtained from the CYP1A2 MD simulations.

9 Active site cluster of quantum mechanics simulation

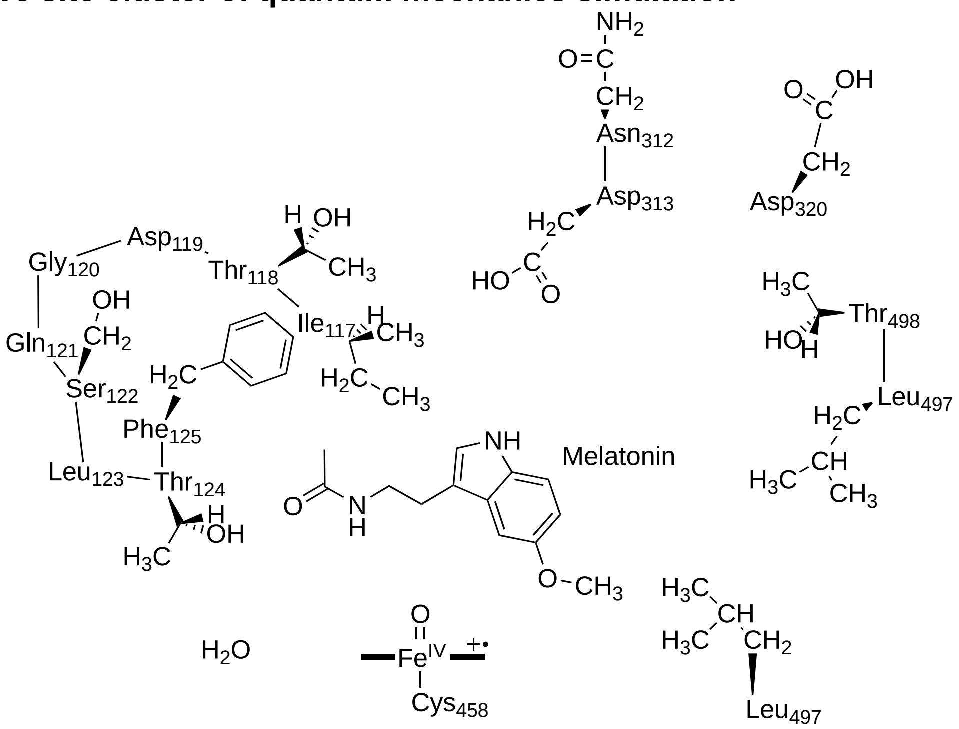

Figure S15 QM active site cluster of 283 atoms used in this work. It was selected from RE_6 at the lowest total energy (80.4 ns).

10 CYP1A1 molecular dynamics Simulation in 1 µs

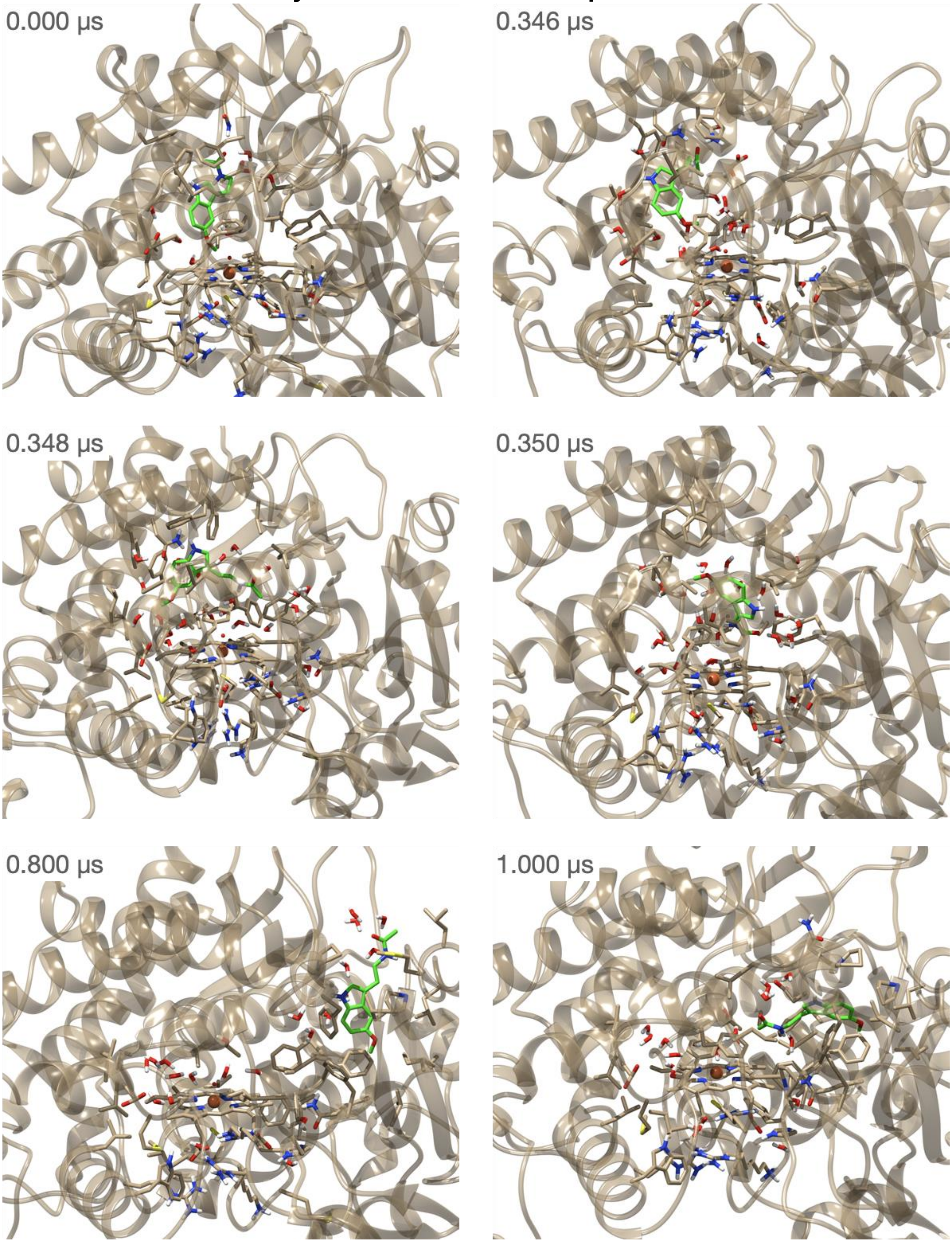

Figure S16 Binding poses of the substrate in the reactant complex in CYP1A1 as obtained from MD simulations. The starting structure was taken from Model 3 of Mokkawes et al (J. Phys. Chem. A 2022).

11 Model 6 Reactant of CYP1A2 molecular dynamics Simulation in 1 μs

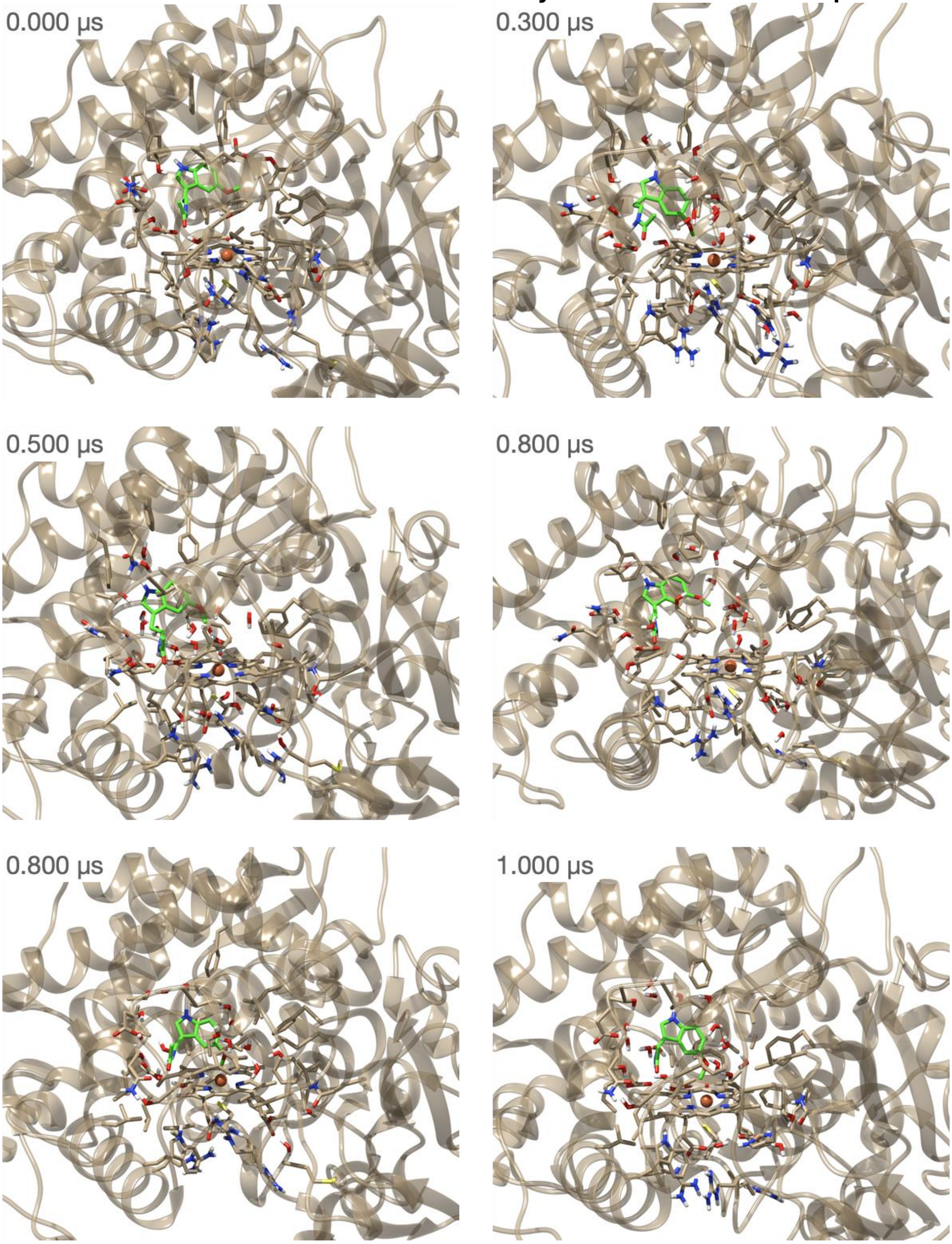

Figure S17 Binding poses of the substrate in the reactant complex in CYP1A1 as obtained from MD simulations. The starting structure was taken from Model 6 of the reactant complex discussed above.

12 Comparison of CYP1A1 and CYP1A2 from Molecular Dynamics Simulation

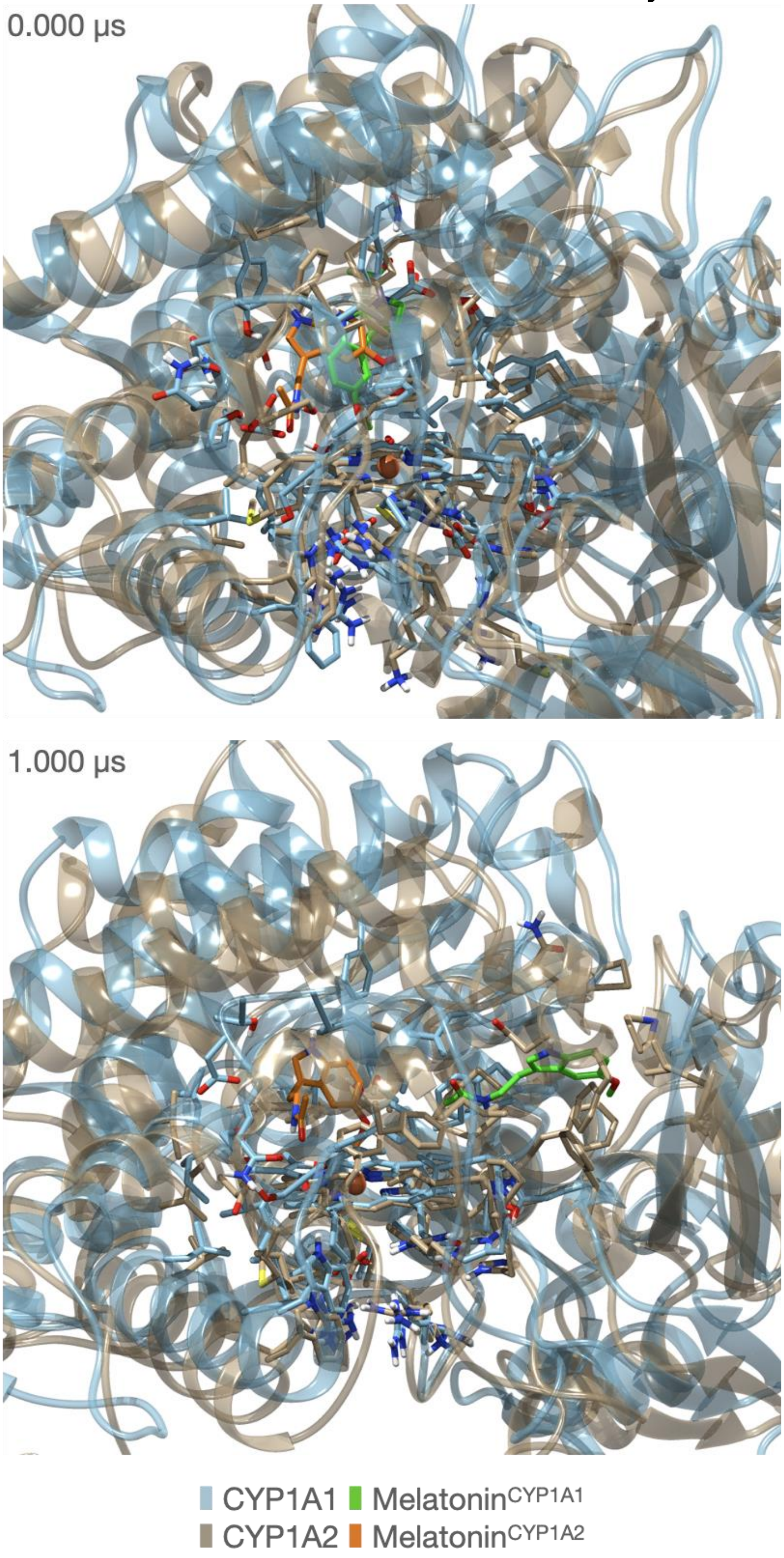

Figure S18 Overlay of the start and end point structures of the 1 μs MD simulation for CYP1A1 and CYP1A2.

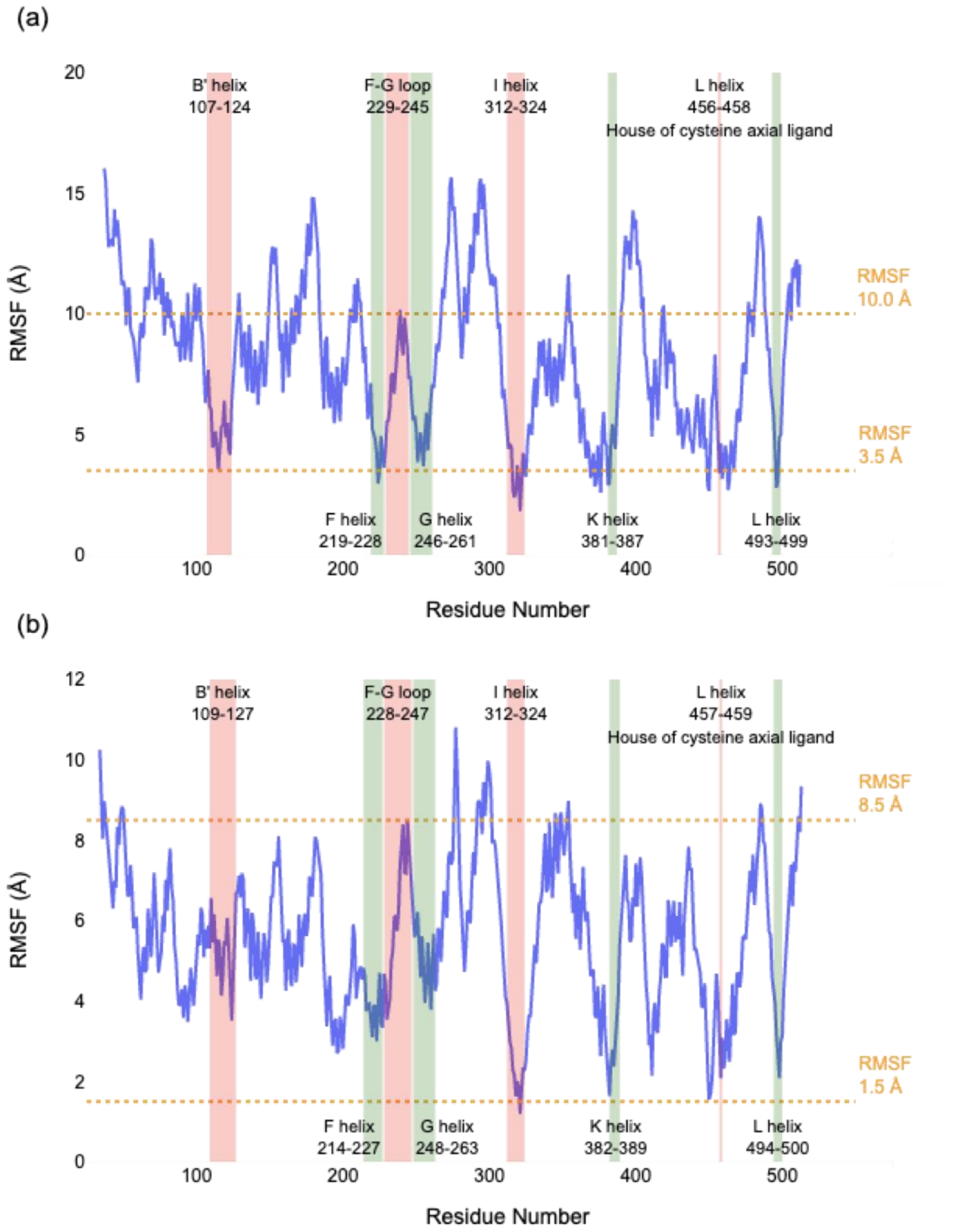

Figure S19 RMSF values of the 1 μs MD simulation for CYP1A1 and CYP1A2.

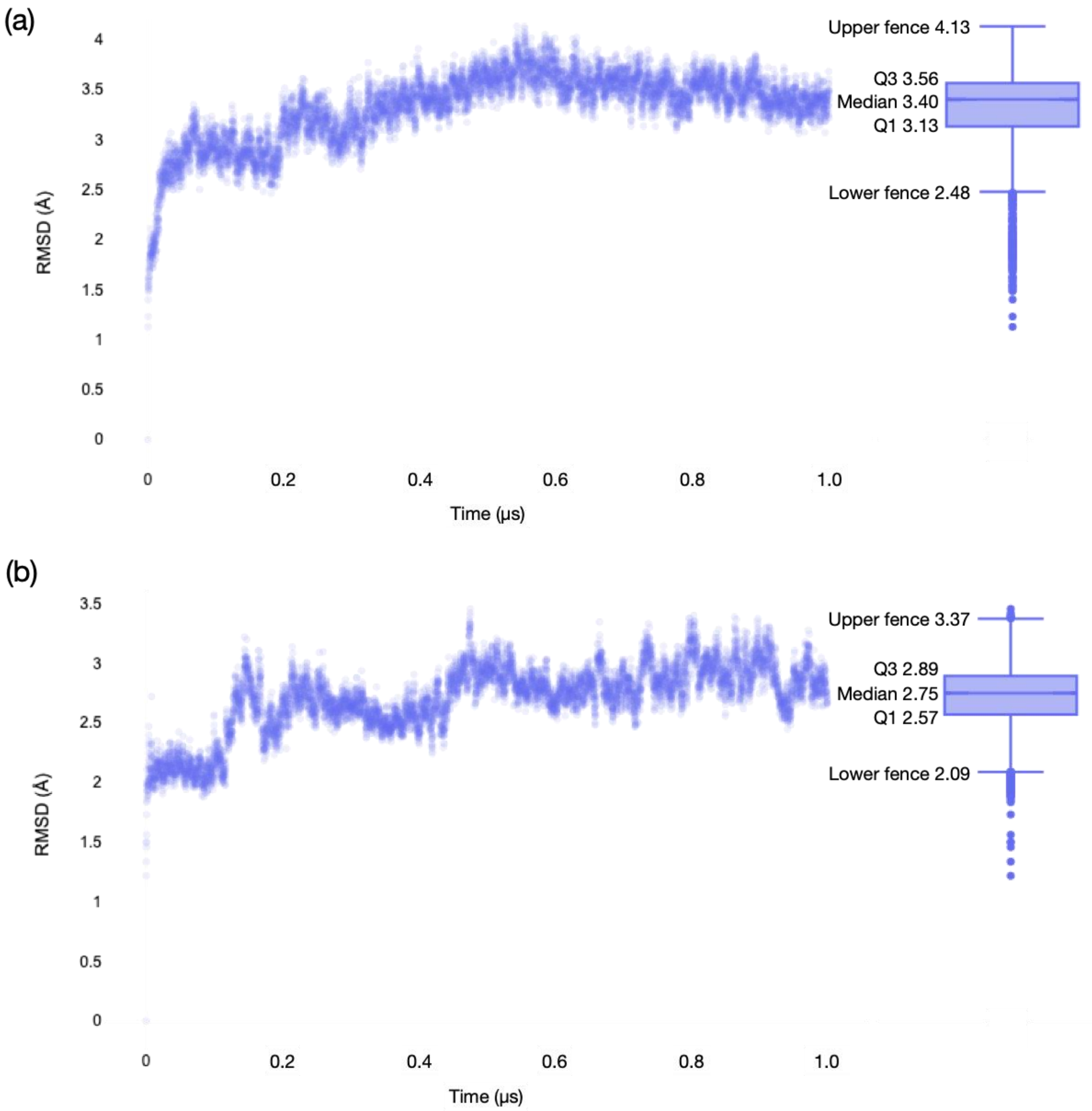

Figure S20 Enzyme RMSD values of the 1 μs MD simulations for CYP1A1 and CYP1A2.

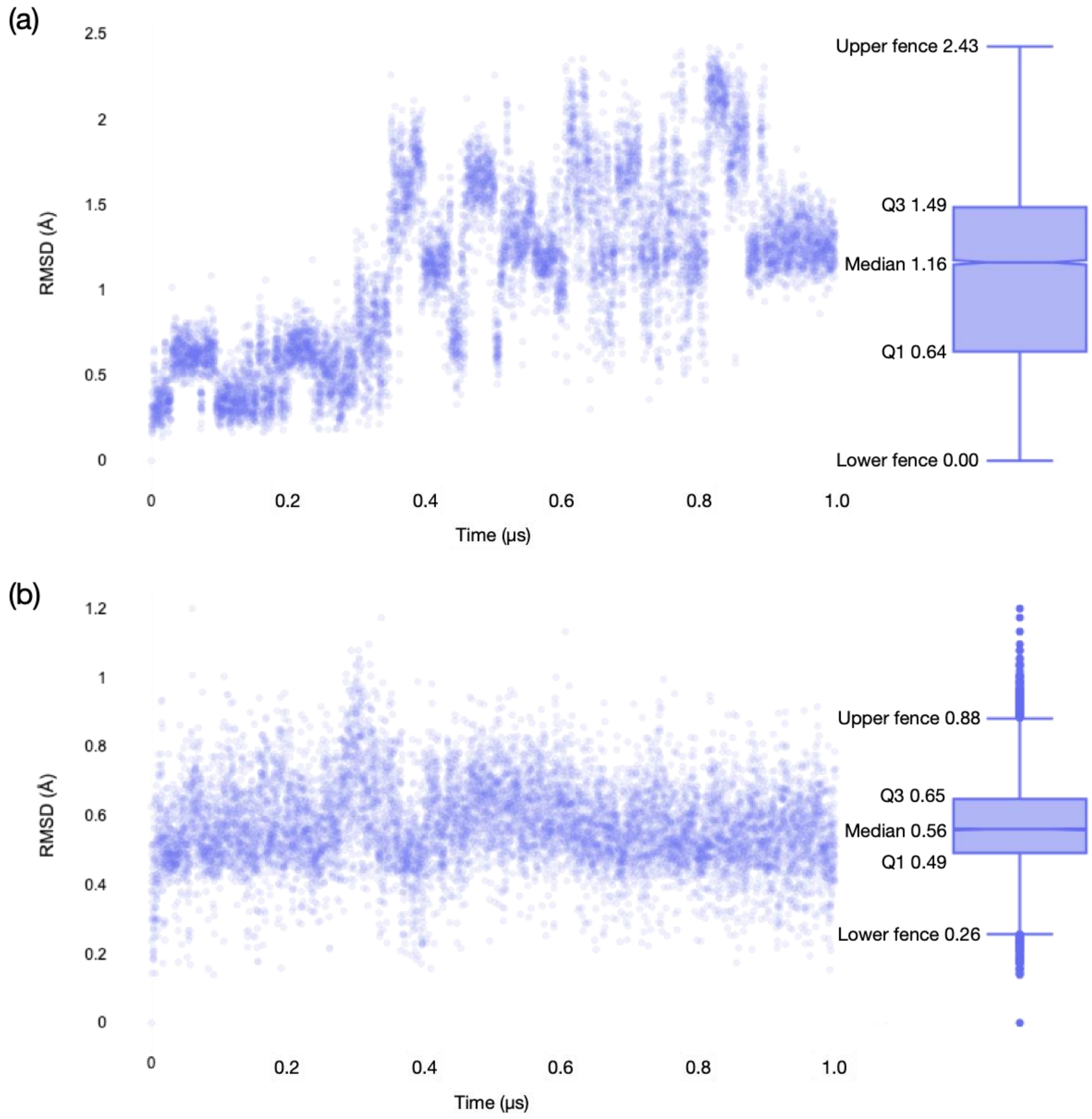

Figure S21 Substrate melatonin RMSD values of the 1 μs MD simulations for CYP1A1 and CYP1A2.

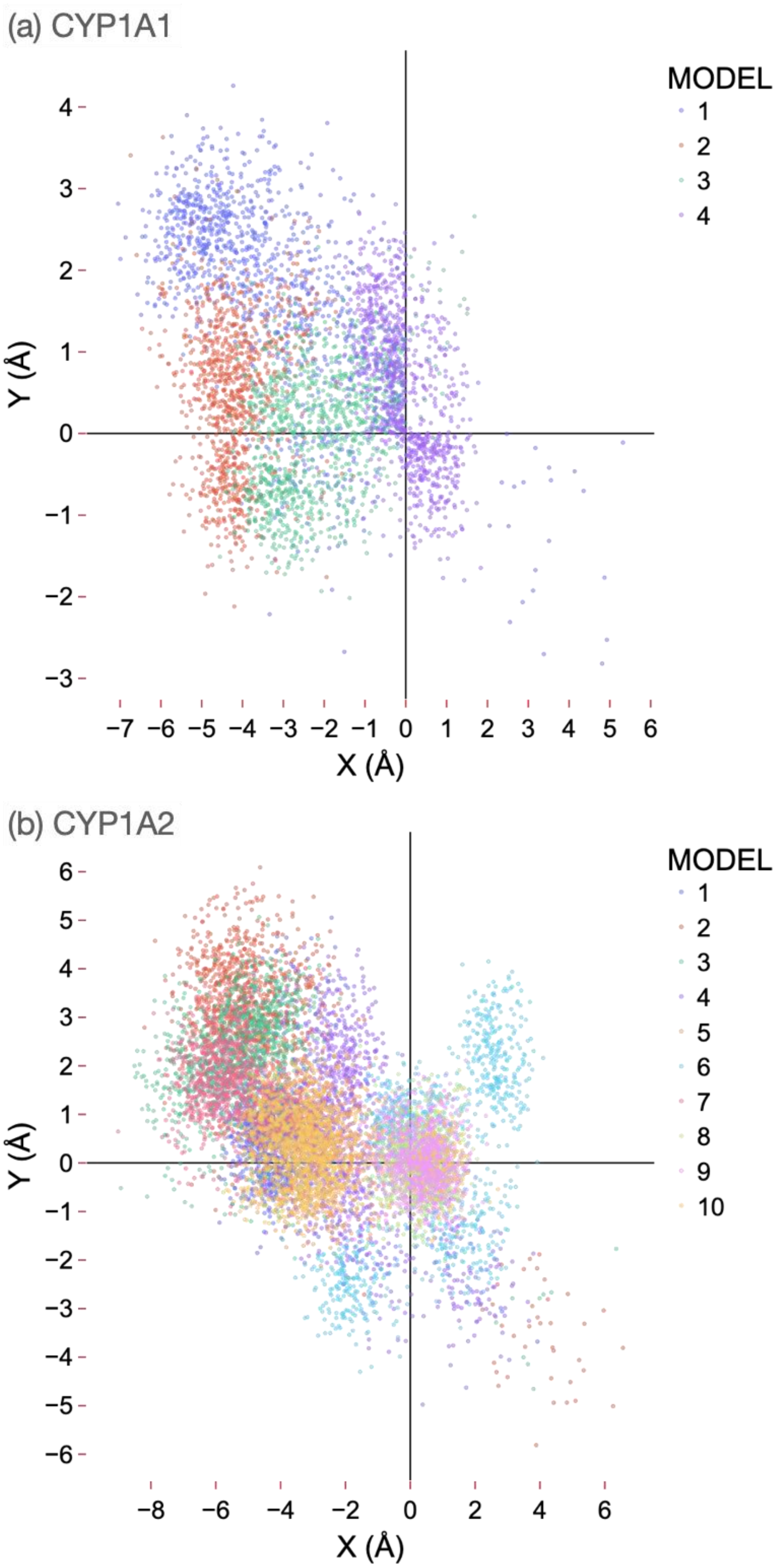

Figure S22 Substrate mapping of the reactant models as obtained from MD simulations for CYP1A1 and CYP1A2.

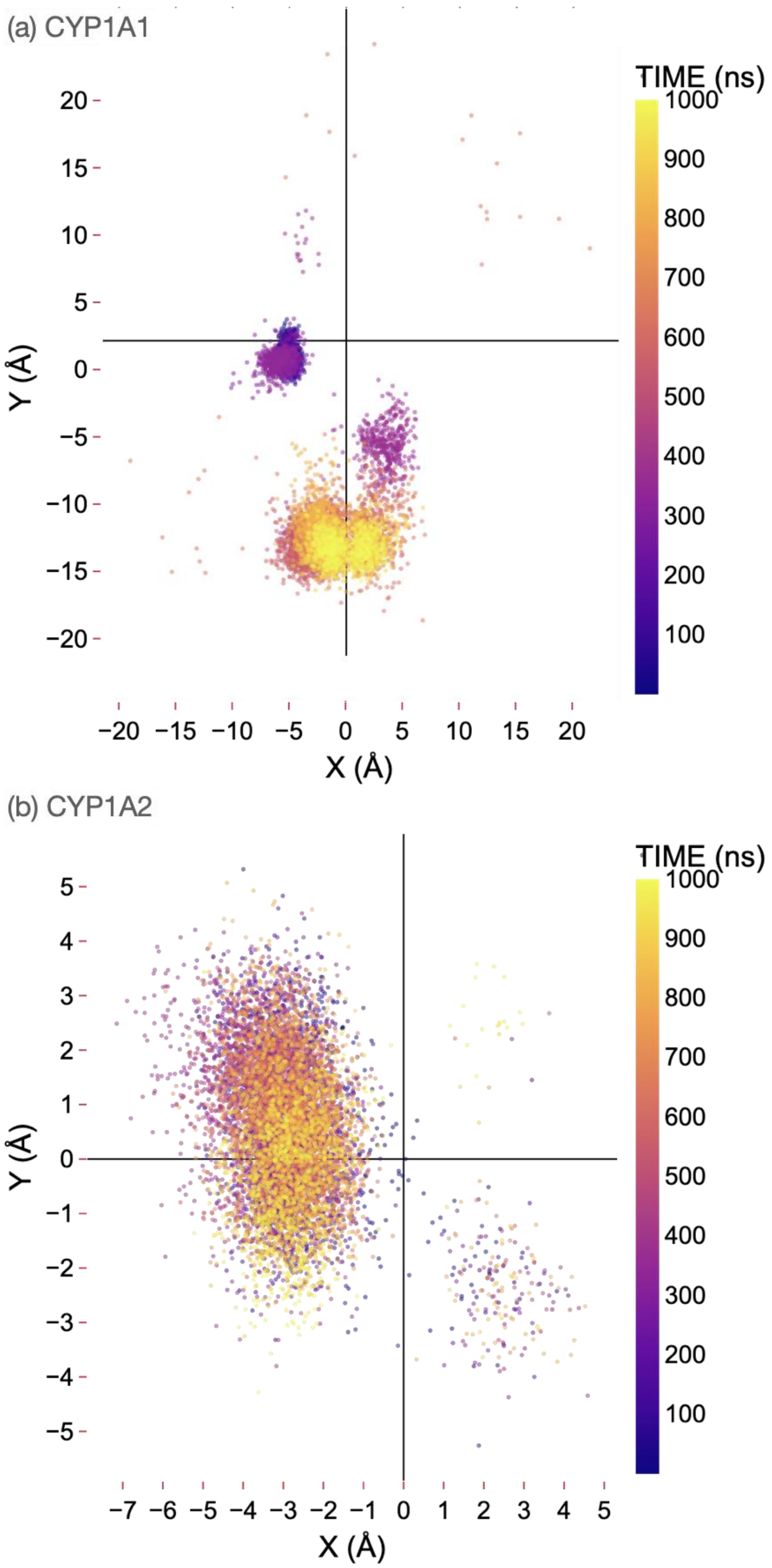

Figure S23 Substrate mapping of reactant models as obtained from the 1 μs MD simulations for CYP1A1 and CYP1A2.

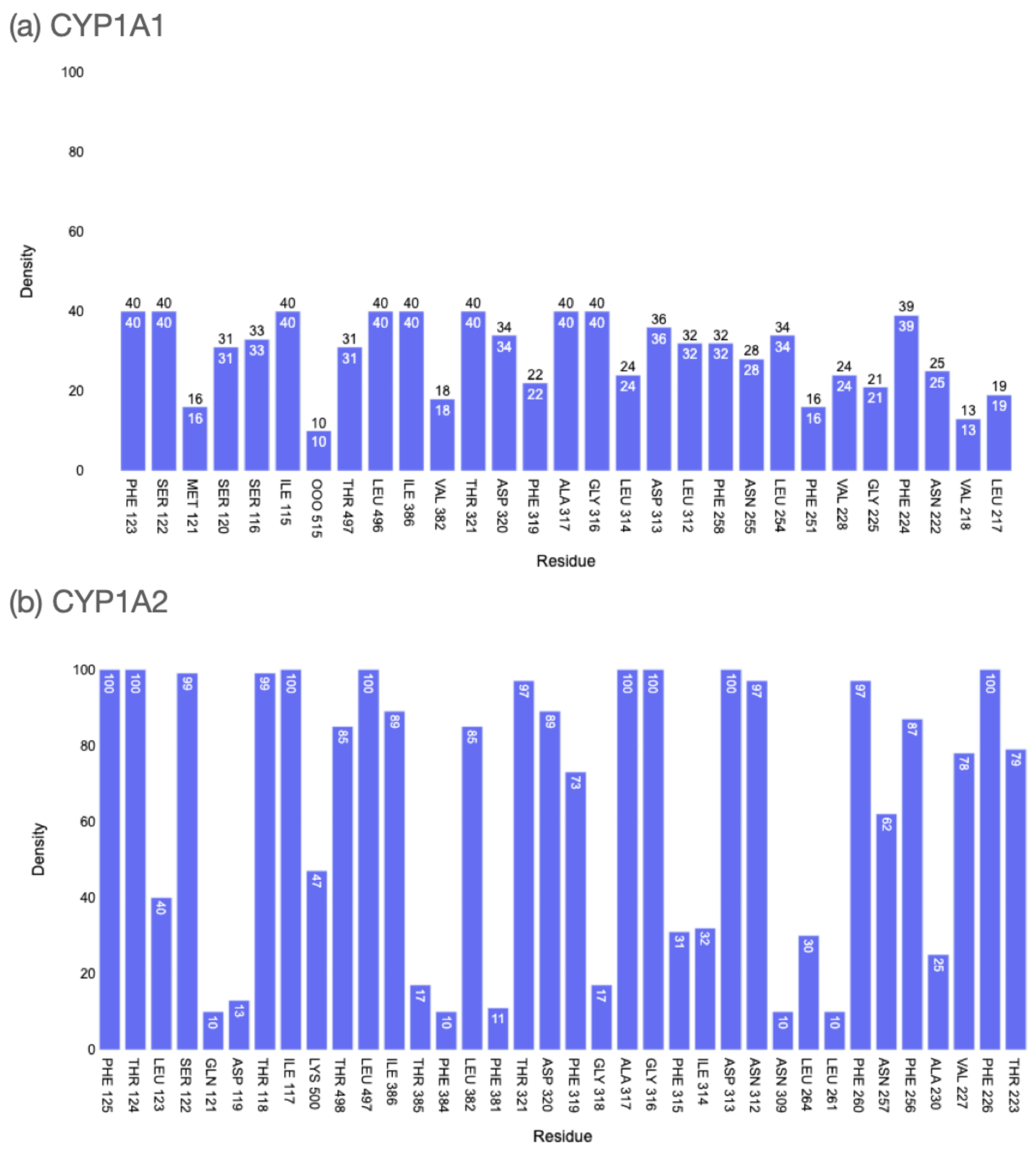

Figure S24 Statistical distribution of amino acid residues positioned within 5 Å from melatonin substrate in the molecular dynamics simulations of the 4 reactant models of CYP1A1 and the 10 models of CYP1A2. Densities in %.

13 Scanning from quantum mechanics calculation

(a)

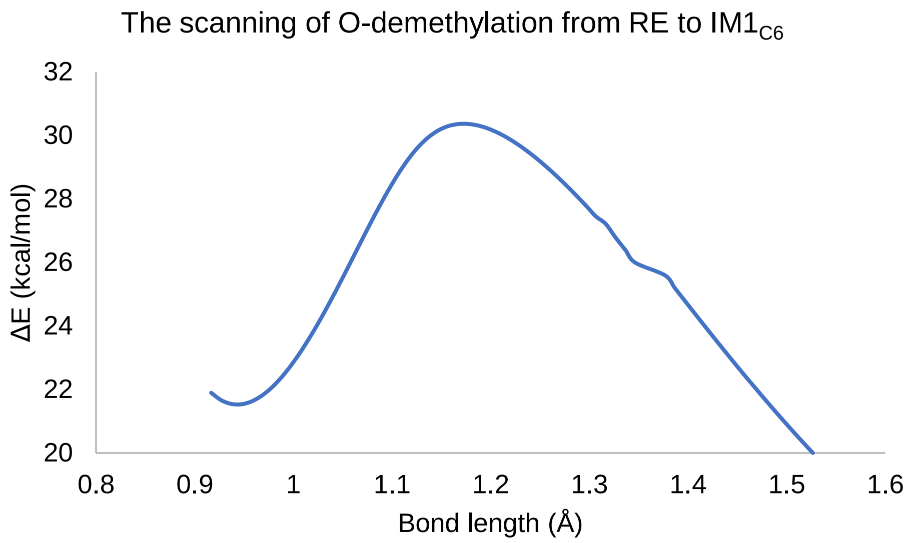

(b)

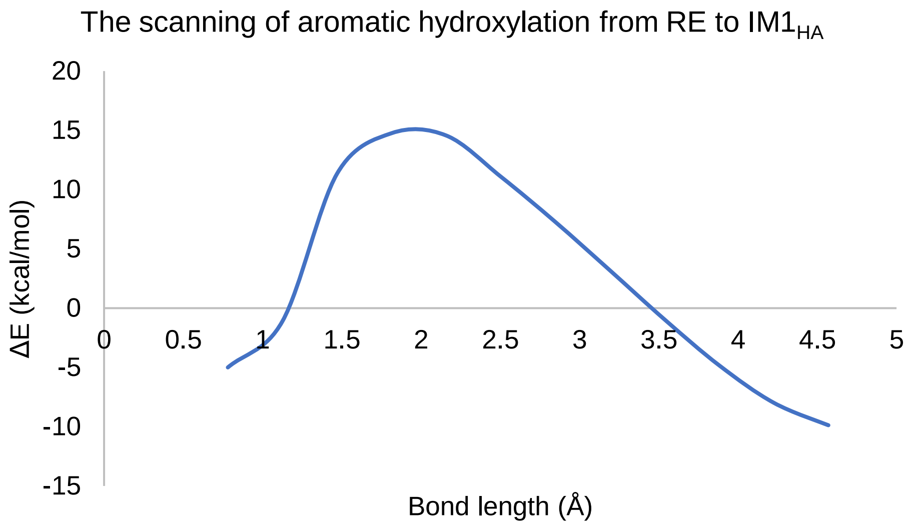

Figure S25 Scanning from quantum mechanics calculations. Each data point represents a full geometry optimization with one degree of freedom (the reaction coordinate) fixed.

14 Energy data from quantum mechanics calculation

Table S1 Absolute energies in kcal mol^{-1} of UB3LYP/BS1 optimized geometries for the metabolism of melatonin by CYP1A2 through aromatic hydroxylation in the doublet and quartet spin states.

CAL	Energy	State					MUL
		**RE**	**TS1$_{C6}$**	**IM1$_{C6}$**	**IM2$_{C6}$**	**P$_{C6}$**	
BS1	ZPE	1484.549	1484.561	1485.443	1487.218	1486.983	2
	E	-4556160.818	-4556145.862	-4556183.697	-4556187.538	-4556209.768	
	E+ZPE	-4554676.269	-4554661.300	-4554698.254	-4554700.320	-4554722.785	
	G	-4554821.969	-4554802.893	-4554844.535	-4554837.643	-4554863.286	
BS2_SOL	E	-5273049.633	-5273045.172	-5273078.670	-5273081.505	-5273101.941	
	E+ZPE	-5271565.084	-5271560.611	-5271593.227	-5271594.287	-5271614.958	
	G	-5271710.784	-5271702.204	-5271739.508	-5271731.610	-5271755.460	
BS1	ZPE	1484.553	1484.803	1483.859	1485.341	1484.780	4
	E	-4556160.729	-4556142.630	-4556170.360	-4556182.697	-4556197.669	
	E+ZPE	-4554676.176	-4554657.827	-4554686.501	-4554697.356	-4554712.888	
	G	-4554822.331	-4554798.990	-4554834.141	-4554838.216	-4554855.959	
BS2_SOL	E	-5273050.261	-5273041.624	-5273064.116	-5273077.967	-5273099.272	
	E+ZPE	-5271565.708	-5271556.821	-5271580.257	-5271592.626	-5271614.492	
	G	-5271711.863	-5271697.984	-5271727.898	-5271733.487	-5271757.562	

Table S2 Absolute energies in kcal mol^{-1} of UB3LYP/BS1 optimized geometries for the metabolism of melatonin by CYP1A2 through *O*-demethylation in the doublet and quartet spin state.

CAL	Energy	State				MUL
		**RE**	**TS1$_{HA}$**	**IM1$_{HA}$**	**IM2**	
BS1	ZPE	1484.549	1479.619	1481.210	1485.942	2
	E	-4556160.818	-4556124.127	-4556132.678	-4556197.813	
	E+ZPE	-4554676.269	-4554644.508	-4554651.467	-4554711.871	
	G	-4554821.969	-4554788.926	-4554796.824	-4554858.175	
BS2_SOL	E	-5273049.633	-5273028.234	-5273033.757	-5273098.633	
	E+ZPE	-5271565.084	-5271548.615	-5271552.546	-5271612.691	
	G	-5271710.784	-5271693.032	-5271697.903	-5271758.995	
BS1	ZPE	1484.553	1480.201	1482.271	1485.373	4
	E	-4556160.729	-4556123.845	-4556132.951	-4556198.009	
	E+ZPE	-4554676.176	-4554643.643	-4554650.680	-4554712.636	
	G	-4554822.331	-4554787.634	-4554797.893	-4554859.431	
BS2_SOL	E	-5273050.261	-5273026.520	-5273039.291	-5273102.415	
	E+ZPE	-5271565.708	-5271546.319	-5271557.020	-5271617.042	
	G	-5271711.863	-5271690.309	-5271704.233	-5271763.837	

Table S3 Relative energies in kcal mol^{-1} of UB3LYP/BS1 optimized geometries for the metabolism of melatonin by CYP1A2 through aromatic hydroxylation in the doublet and quartet spin state.

CAL	Energy	State					MUL
		RE	$TS1_{C6}$	$IM1_{C6}$	$IM2_{C6}$	P_{C6}	
BS1	ΔE	0.000	14.957	-22.878	-26.720	-48.950	2
	Δ(E+ZPE)	0.000	14.969	-21.984	-24.051	-46.515	
	ΔG	0.000	19.076	-22.566	-15.674	-41.317	
BS2_SOL	ΔE	0.000	4.461	-29.037	-31.872	-52.308	
	Δ(E+ZPE)	0.000	4.474	-28.143	-29.202	-49.874	
	ΔG	0.000	8.580	-28.724	-20.825	-44.676	
BS1	ΔE	0.089	18.188	-9.541	-21.879	-36.850	4
	Δ(E+ZPE)	0.093	18.443	-10.232	-21.086	-36.619	
	ΔG	-0.362	22.979	-12.172	-16.247	-33.990	
BS2_SOL	ΔE	-0.628	8.009	-14.483	-28.334	-49.639	
	Δ(E+ZPE)	-0.624	8.264	-15.173	-27.541	-49.407	
	ΔG	-1.079	12.800	-17.114	-22.703	-46.778	

Table S4 Relative energies in kcal mol^{-1} of UB3LYP/BS1 optimized geometries for the metabolism of melatonin by CYP1A2 through *O*-demethylation in the doublet and quartet spin state.

CAL	Energy	State				MUL
		RE	$TS1_{HA}$	$IM1_{HA}$	IM2	
BS1	ΔE	0.000	36.691	28.141	-36.995	2
	Δ(E+ZPE)	0.000	31.761	24.802	-35.602	
	ΔG	0.000	33.043	25.145	-36.206	
BS2_SOL	ΔE	0.000	21.399	15.876	-49.000	
	Δ(E+ZPE)	0.000	16.470	12.538	-47.606	
	ΔG	0.000	17.752	12.881	-48.211	
BS1	ΔE	0.089	36.973	27.868	-37.191	4
	Δ(E+ZPE)	0.093	32.626	25.590	-36.366	
	ΔG	-0.362	34.335	24.076	-37.462	
BS2_SOL	ΔE	-0.628	23.113	10.343	-52.782	
	Δ(E+ZPE)	-0.624	18.766	8.065	-51.957	
	ΔG	-1.079	20.475	6.551	-53.053	

15 Energy data from quantum mechanics calculation with RRHO fixed

Table S5 Absolute energies with the correction in kcal mol^{-1} of UB3LYP/BS1 optimized geometries for the metabolism of melatonin by CYP1A2 through aromatic hydroxylation in the doublet and quartet spin states.

CAL	Energy	State					MUL
		RE	$TS1_{C6}$	$IM1_{C6}$	$IM2_{C6}$	P_{C6}	
BS1	G	-4554821.969	-4554802.893	-4554844.535	-4554837.643	-4554863.286	2
	G_{Cr}	-4554812.403	-4554794.879	-4554833.892	-4554831.325	-4554855.788	
BS2_SOL	G	-5271710.784	-5271702.204	-5271739.508	-5271731.610	-5271755.460	
	G_{Cr}	-5271701.218	-5271694.190	-5271728.866	-5271725.291	-5271747.961	
BS1	G	-4554822.331	-4554798.990	-4554834.141	-4554838.216	-4554855.959	4
	G_{Cr}	-4554812.723	-4554791.224	-4554823.373	-4554831.325	-4554847.950	
BS2_SOL	G	-5271711.863	-5271697.984	-5271727.898	-5271733.487	-5271757.562	
	G_{Cr}	-5271701.226	-5271690.218	-5271717.129	-5271725.291	-5271749.553	

G_{Cr} is free energy with rroh correction

Table S6 Absolute energies with the correction in kcal mol^{-1} of UB3LYP/BS1 optimized geometries for the metabolism of melatonin by CYP1A2 through *O*-demethylation in the doublet and quartet spin state.

CAL	Energy	State				MUL
		RE	$TS1_{HA}$	$IM1_{HA}$	IM2	
BS1	G	-4554821.969	-4554788.926	-4554796.824	-4554858.175	2
	G_{Cr}	-4554812.403	-4554779.557	-4554787.152	-4554848.087	
BS2_SOL	G	-5271710.784	-5271693.032	-5271697.903	-5271758.995	
	G_{Cr}	-5271701.218	-5271683.664	-5271688.230	-5271748.906	
BS1	G	-4554822.331	-4554787.634	-4554797.893	-4554859.431	4
	G_{Cr}	-4554812.723	-4554778.606	-4554787.607	-4554849.552	
BS2_SOL	G	-5271711.863	-5271690.309	-5271704.233	-5271763.837	
	G_{Cr}	-5271702.666	-5271681.282	-5271693.947	-5271753.959	

G_{Cr} is free energy with rroh correction

Table S7 Relative energies with the correction in kcal mol^{-1} of UB3LYP/BS1 optimized geometries for the metabolism of melatonin by CYP1A2 through aromatic hydroxylation in the doublet and quartet spin state.

CAL	Energy	State					MUL
		RE	$TS1_{C6}$	$IM1_{C6}$	$IM2_{C6}$	P_{C6}	
BS1	ΔG	0.000	19.076	-22.566	-15.674	-41.317	2
	ΔG_{Cr}	0.000	17.524	-21.490	-18.922	-43.385	
BS2_SOL	ΔG	0.000	8.580	-28.724	-20.826	-44.676	
	ΔG_{Cr}	0.000	7.029	-27.647	-24.073	-46.743	
BS1	ΔG	-0.362	22.979	-12.172	-16.247	-33.990	4
	ΔG_{Cr}	-0.320	21.179	-10.970	-18.922	-35.547	
BS2_SOL	ΔG	-1.079	12.800	-17.114	-22.703	-46.778	
	ΔG_{Cr}	-0.008	11.000	-15.911	-24.073	-48.335	

G_{Cr} is free energy with rroh correction

Table S8 Relative energies with the correction in kcal mol^{-1} of UB3LYP/BS1 optimized geometries for the metabolism of melatonin by CYP1A2 through *O*-demethylation in the doublet and quartet spin state.

CAL	Energy	State				MUL
		RE	$TS1_{HA}$	$IM1_{HA}$	IM2	
BS1	G	0.000	33.043	25.145	-36.206	2
	G_{Cr}	0.000	32.846	25.251	-35.684	
BS2_SOL	G	0.000	17.752	12.881	-48.211	
	G_{Cr}	0.000	17.555	12.988	-47.688	
BS1	G	-0.362	34.335	24.076	-37.462	4
	G_{Cr}	-0.320	33.796	24.796	-37.150	
BS2_SOL	G	-1.079	20.475	6.551	-53.053	
	G_{Cr}	-1.448	19.936	7.272	-52.740	

G_{Cr} is free energy with rroh correction

16 Group charges and spin densities from quantum mechanics simulation

Table S9 Group charges (doublet/quartet) of UB3LYP/BS1 optimized geometries for the metabolism of melatonin by CYP1A2 through aromatic hydroxylation in the doublet and quartet spin state.

Residue group	Composition	RE	$TS1_{C6}$	$IM1_{C6}$	$IM2_{C6}$	P_{C6}
Compound I	Fe	0.479 / 0.471	0.413 / 0.432	0.274 / 0.461	0.264 / 0.428	0.238 / 0.447
	O	-0.422 / -0.418	-0.46 / -0.479	-0.557 / -0.546	-0.562 / -0.57	0.158 / 0.158
	Fe-O*	0.058 / 0.053	-0.047 / -0.047	-0.284 / -0.085	-0.298 / -0.142	0.396 / 0.605
	Por*	-0.054 / -0.047	-0.346 / -0.375	-0.559 / -0.519	-0.55 / -0.748	-0.417 / -0.52
	S	-0.189 / -0.191	-0.207 / -0.164	-0.177 / -0.349	-0.17 / -0.15	0.554 / 0.554
	S-H*	-0.101 / -0.104	-0.13 / -0.083	-0.111 / -0.277	-0.109 / -0.08	1.008 / 1.002
Melatonin	C_6*	-0.199 / -0.199	-0.129 / -0.082	0.102 / 0.103	0.101 / 0.098	0.323 / 0.326
	H_{C6}*	0.16 / 0.16	0.179 / 0.169	0.141 / 0.118	0.148 / 0.15	0.174 / 0.173
	The rest atoms*	-0.04 / -0.04	0.28 / 0.229	0.497 / 0.416	0.5 / 0.514	-0.832 / -0.941
Environment*		-1.824 / -1.824	-1.808 / -1.813	-1.787 / -1.755	-1.792 / -1.793	-2.651 / -2.644
**Total****		-2 / -2	-2 / -2	-2 / -2	-2 / -2	-2 / -2

**** is the summation of ***

Table S10 Group charges (doublet/quartet) of UB3LYP/BS1 optimized geometries for the metabolism of melatonin by CYP1A2 through *O*-demethylation in the doublet and quartet spin state.

Residue group	Composition	RE	$TS1_{HA}$	$IM1_{HA}$	IM2
Compound I	Fe	0.479 / 0.471	0.356 / 0.415	0.324 / 0.405	0.306 / 0.49
	O	-0.422 / -0.418	-0.6 / -0.596	-0.696 / -0.678	0.164 / 0.165
	Fe-O*	0.058 / 0.053	-0.244 / -0.181	-0.372 / -0.273	0.47 / 0.656
	Por*	-0.054 / -0.047	-0.102 / -0.247	-0.075 / -0.283	-0.36 / -0.376
	S	-0.189 / -0.191	-0.185 / -0.104	-0.184 / -0.091	0.534 / 0.534
	S-H*	-0.101 / -0.104	-0.101 / -0.023	-0.1 / -0.001	0.985 / 0.983
Melatonin	C_{Meth}*	-0.236 / -0.235	-0.246 / -0.262	-0.164 / -0.173	0.158 / 0.158
	H_{CMeth}*	0.219 / 0.218	0.389 / 0.396	0.412 / 0.441	0.154 / 0.153
	The rest atoms*	-0.061 / -0.061	0.12 / 0.129	0.112 / 0.103	-0.883 / -1.053
Environment*		-1.824 / -1.824	-1.816 / -1.812	-1.813 / -1.815	-2.523 / -2.521
**Total****		-2 / -2	-2 / -2	-2 / -2	-2 / -2

**** is the summation of ***

Table S11 Group spin densities (doublet/quartet) of UB3LYP/BS1 optimized geometries for the metabolism of melatonin by CYP1A2 through aromatic hydroxylation in the doublet and quartet spin state.

Residue group	Composition	RE	$TS1_{C6}$	$IM1_{C6}$	$IM2_{C6}$	P_{C6}
Compound I	Fe	1.239 / 1.113	1.418 / 1.44	0.992 / 2.573	0.988 / 2.988	1.123 / 3.253
	O	0.856 / 0.902	0.352 / 0.722	0.044 / 0.103	0.05 / 0.026	0 / 0
	Fe-O*	2.095 / 2.015	1.769 / 2.162	1.036 / 2.677	1.039 / 3.015	1.123 / 3.253
	Por*	-0.593 / 0.527	-0.21 / -0.002	-0.076 / -0.058	-0.075 / 0.14	-0.089 / -0.083
	S	-0.518 / 0.468	-0.172 / 0.233	0.035 / 0.358	0.037 / -0.163	0 / 0
	S-H*	-0.503 / 0.455	-0.167 / 0.229	0.033 / 0.35	0.034 / -0.163	0 / -0.001
Melatonin	C_6*	0 / 0.001	-0.12 / 0.046	-0.003 / -0.001	-0.004 / -0.003	0.001 / -0.003
	H_{C6}*	-0.001 / -0.001	0.006 / 0.009	0 / 0.009	0 / 0.001	0 / 0
	The rest atoms*	0.002 / 0.002	-0.277 / 0.555	0.011 / 0.023	0.005 / 0.01	-0.027 / -0.166
Environment*		0 / 0.001	-0.001 / 0.002	0 / 0	0 / 0	-0.008 / 0
**Total****		1 / 3	1 / 3	1 / 3	1 / 3	1 / 3

**** is the summation of ***

Table S12 Group spin densities (doublet/quartet) of UB3LYP/BS1 optimized geometries for the metabolism of melatonin by CYP1A2 through *O*-demethylation in the doublet and quartet spin state.

Residue group	Composition	RE	$TS1_{HA}$	$IM1_{HA}$	IM2
Compound I	Fe	1.239 / 1.113	1.022 / 1.295	0.992 / 1.755	1.222 / 2.49
	O	0.856 / 0.902	0.498 / 0.633	0.093 / 0.271	0 / 0
	Fe-O*	2.095 / 2.015	1.52 / 1.929	1.085 / 2.025	1.222 / 2.49
	Por*	-0.593 / 0.527	-0.616 / 0.157	-0.702 / -0.095	-0.14 / 0.06
	S	-0.518 / 0.468	-0.319 / 0.35	-0.325 / 0.096	0 / 0
	S-H*	-0.503 / 0.455	-0.311 / 0.342	-0.317 / 0.096	0 / -0.002
Melatonin	C_{Meth}*	0.002 / 0.003	0.386 / 0.424	0.848 / 0.846	0 / 0
	H_{Meth}*	-0.001 / -0.002	-0.059 / -0.037	0.009 / 0.021	0 / 0
	The rest atoms*	0 / 0.001	0.079 / 0.185	0.074 / 0.102	-0.081 / 0.458
Environment*		0 / 0.001	0.001 / 0	0.004 / 0.005	-0.001 / -0.006
**Total****		1 / 3	1 / 3	1 / 3	1 / 3

**** is the summation of ***

17 Imaginary frequency data from quantum mechanics simulation

Table S13 Imaginary frequencies for the metabolism of melatonin by CYP1A2 through aromatic hydroxylation and *O*-demethylation.

State	Imaginary frequency
^{2,4}**TS1$_{C6}$**	i240.44 (Doublet), i410.71 (Quartet)
^{2,4}**TS1$_{HA}$**	i1602.02 (Doublet), i1775.64 (Quartet)

18 Geometry data from quantum mechanics simulation

Table S14 Geometry data (doublet/quartet) of UB3LYP/BS1 optimized geometries for the metabolism of melatonin by CYP1A2 through aromatic hydroxylation in the doublet and quartet spin state. Bond length is Å and angle is degree.

Composition	RE	TS1$_{C6}$	IM1$_{C6}$	IM2$_{C6}$	P$_{C6}$
Fe-O$_{CpdI}$	1.629 / 1.631	1.679 / 1.715	1.922 / 1.933	1.927 / 1.920	2.356 / 2.595
Fe-S$_{CpdI}$	2.575 / 2.562	2.434 / 2.403	2.322 / 2.353	2.318 / 2.393	2.242 / 2.339
C$_6$-O$_{CpdI}$	3.743 / 3.739	2.052 / 1.887	1.387 / 1.388	1.326 / 1.326	1.387 / 1.376
H$_{C6}$-C$_6$	1.086 / 1.086	1.082 / 1.083	1.110 / 1.110	2.163 / 2.188	1.939 / 1.936
H$_{C6}$-N$_{CpdI}$	5.239 / 5.232	2.467 / 2.624	2.415 / 2.419	1.043 / 1.028	3.058 / 2.998
H$_{C6}$-O$_{CpdI}$	2.888 / 2.875	2.275 / 2.192	2.081 / 2.083	2.304 / 2.241	0.974 / 0.973
C$_6$-O$_{CpdI}$-Fe	156.966 / 157.370	126.974 / 136.423	126.884 / 126.325	129.186 / 135.900	132.156 / 134.214
C$_6$-H$_{C6}$-N$_{CpdI}$	150.798 / 151.609	136.892 / 126.877	118.091 / 118.952	144.397 / 154.665	85.503 / 91.265

Table S15 Geometry data (doublet/quartet) of UB3LYP/BS1 optimized geometries for the metabolism of melatonin by CYP1A2 through *O*-demethylation in the doublet and quartet spin state. Bond lengths are in Å and angles in degrees.

Composition	RE	TS1$_{HA}$	IM1$_{HA}$	IM2
Fe-O$_{CpdI}$	1.629 / 1.631	1.755 / 1.731	1.812 / 1.786	3.381 / 3.469
Fe-S$_{CpdI}$	2.575 / 2.562	2.440 / 2.383	2.431 / 2.329	2.236 / 2.429
H$_{CMeth}$-O$_{CpdI}$	2.369 / 2.384	1.175 / 1.187	0.975 / 0.981	0.974 / 0.973
H$_{Cmeth}$-C$_{Meth}$	1.095 / 1.095	1.354 / 1.375	2.397 / 2.311	1.956 / 1.956
O$_{CpdI}$-C$_{Meth}$	3.434 / 3.447	2.518 / 2.543	3.203 / 3.201	1.392 / 1.391
H$_{CMeth}$-O$_{CpdI}$-Fe	141.215 / 141.387	116.977 / 123.516	105.949 / 109.607	52.602 / 50.622
H$_{CMeth}$-O$_{CpdI}$-C$_{Meth}$	5.201 / 5.249	5.796 / 7.641	29.018 / 20.837	110.352 / 110.408

Geometry coordination

RE^{2}

6	2.719429000	26.652134000	18.197097000
6	6.914258000	28.986224000	17.600464000
6	8.246242000	27.743597000	22.080341000
6	3.995356000	25.526777000	22.719282000
6	3.762419000	27.387266000	17.638455000
6	3.740216000	27.936450000	16.304209000
6	4.919257000	28.595863000	16.136177000
6	5.656350000	28.444798000	17.367701000
6	7.647384000	28.859436000	18.774646000
6	8.965251000	29.405061000	18.981516000
6	9.345168000	29.042877000	20.239120000
6	8.254377000	28.284798000	20.798845000
6	7.193276000	27.053154000	22.657769000
6	7.193392000	26.529791000	24.001216000
6	5.994287000	25.912276000	24.184313000
6	5.260658000	26.051140000	22.950534000
6	3.281004000	25.636337000	21.532648000
6	1.959191000	25.085805000	21.325685000
6	1.612470000	25.394537000	20.046995000
6	2.716039000	26.139694000	19.485943000
7	4.934576000	27.705250000	18.267928000
7	7.229084000	28.187478000	19.894371000
7	5.998589000	26.763135000	22.036001000
7	3.724662000	26.280868000	20.405725000
26	5.526787000	27.105857000	20.096882000
1	1.830607000	26.467448000	17.598152000
1	7.367920000	29.551414000	16.791271000
1	9.135098000	27.891781000	22.686671000
1	3.521530000	24.983391000	23.531190000
6	5.964538000	19.705412000	17.895444000
6	6.717509000	20.689617000	17.231338000
6	6.004312000	21.056151000	19.962521000
6	5.626964000	19.903837000	19.265427000
6	5.370920000	18.454370000	17.482556000
6	7.080236000	21.833589000	17.930746000
6	6.729316000	22.028952000	19.280816000
7	4.901286000	18.800909000	19.677557000
1	4.268204000	18.754995000	20.481929000
6	5.441718000	17.860302000	16.101551000
6	4.823474000	16.458563000	15.935891000
7	3.375573000	16.400289000	16.067781000
6	2.553555000	16.697494000	15.030028000
6	1.066599000	16.576013000	15.333335000
8	2.966439000	17.029342000	13.913223000
1	2.963618000	16.155484000	16.969067000
8	7.834616000	22.813075000	17.274561000
6	7.055542000	23.915884000	16.785547000
1	4.069237000	17.104164000	18.700914000
1	7.002188000	20.586050000	16.187884000
1	5.724524000	21.202024000	21.002679000
1	7.014142000	22.954522000	19.771685000
1	4.957147000	18.524745000	15.373134000
1	6.495118000	17.791667000	15.785114000
1	5.252482000	15.771302000	16.674387000
1	5.078061000	16.090643000	14.936777000
1	0.582698000	17.535903000	15.127360000
1	0.865209000	16.284419000	16.367512000
1	0.626206000	15.838944000	14.652366000
1	7.750453000	24.571316000	16.252910000
1	6.586670000	24.466028000	17.608576000
1	6.285796000	23.556797000	16.090978000
16	4.695043000	29.291461000	21.175779000
6	11.206169000	24.106885000	18.798917000
8	12.394192000	24.158595000	19.123916000
7	10.663209000	23.038644000	18.155575000
6	11.452903000	21.846350000	17.880141000
6	12.358852000	21.923624000	16.634882000
6	11.691546000	21.675970000	15.267328000
6	10.619345000	22.715027000	14.903375000
6	12.763764000	21.606647000	14.167314000
1	9.671867000	23.034806000	17.924425000
1	10.758142000	21.002436000	17.794852000
1	13.149229000	21.168805000	16.763309000
1	12.864275000	22.898007000	16.641065000
1	11.199740000	20.691062000	15.313475000
1	10.239006000	22.535301000	13.889383000
1	11.035713000	23.730806000	14.928752000
1	9.765837000	22.682604000	15.585843000
1	12.318094000	21.374705000	13.191980000
1	13.516101000	20.838152000	14.385790000
1	13.288551000	22.567043000	14.072729000
6	7.991776000	17.698789000	22.263219000
6	6.842099000	17.043199000	23.034438000
8	6.968920000	16.675659000	24.203083000
6	9.364850000	17.153861000	22.677970000
6	9.619123000	15.668889000	22.349634000
6	9.581225000	15.378354000	20.841233000
6	10.959091000	15.218017000	22.951007000
1	7.955231000	18.773781000	22.489131000
1	9.461221000	17.290314000	23.761258000
1	10.145501000	17.765745000	22.202905000
1	8.823144000	15.086343000	22.833520000
1	9.786404000	14.319107000	20.641570000
1	8.605860000	15.610626000	20.400979000
1	10.337734000	15.969773000	20.307421000
1	11.141242000	14.151985000	22.764375000
1	10.982353000	15.378902000	24.035614000
1	11.795758000	15.778972000	22.512053000
7	5.681166000	16.922339000	22.331636000
6	4.436256000	16.448852000	22.917352000
6	3.353716000	17.543178000	23.007050000
8	3.007932000	18.022218000	21.713735000
6	3.771609000	18.737867000	23.858475000
1	5.642051000	17.298687000	21.391058000
1	4.682244000	16.062151000	23.909993000
1	2.468797000	17.070989000	23.466349000
1	2.493947000	17.290205000	21.255322000
1	2.957564000	19.469891000	23.896402000
1	4.647137000	19.232627000	23.424696000
1	4.027423000	18.427186000	24.877655000
6	0.457092000	13.274868000	20.490400000
6	-0.722758000	13.591736000	21.411606000
8	-1.640776000	12.793870000	21.599877000
6	0.689881000	14.282582000	19.337864000
6	1.612459000	15.476303000	19.655371000
8	1.618681000	15.904575000	20.860798000
8	2.278681000	15.960986000	18.714334000
1	1.368982000	13.201900000	21.097332000
1	1.123501000	13.762005000	18.478140000
1	-0.270619000	14.696806000	19.002009000
7	-0.663627000	14.820501000	22.012440000
6	-1.826339000	15.403834000	22.664818000
6	-1.715682000	15.325144000	24.197981000
8	-0.441012000	15.864698000	24.605671000
6	-1.896609000	13.916871000	24.761133000
1	0.076182000	15.439508000	21.651313000
1	-2.724027000	14.877429000	22.324586000
1	-2.460422000	15.998802000	24.638777000
1	0.206370000	15.444349000	24.007190000
1	-1.741043000	13.921926000	25.845734000
1	-1.187998000	13.220187000	24.302536000
1	-2.904289000	13.542456000	24.547808000
6	-1.839109000	21.837563000	23.183140000
6	-1.427845000	23.279500000	22.868775000
8	-0.360566000	23.738220000	23.298004000
6	-0.608516000	20.918356000	23.182693000
6	-0.968512000	19.449168000	23.342938000
8	-1.831852000	18.909810000	22.632028000
7	-0.302218000	18.766947000	24.297686000
1	-2.309209000	21.820765000	24.176223000
1	0.092656000	21.255399000	23.950933000
1	-0.084216000	21.013434000	22.223762000
1	0.450795000	19.212349000	24.800672000
1	-0.392907000	17.747221000	24.375201000
7	-2.299446000	23.980593000	22.119351000
6	-2.057386000	25.355298000	21.694201000
6	-3.375751000	26.055715000	21.329398000

6	-4.187012000	25.286672000	20.261197000
8	-4.426800000	25.891079000	19.164954000
8	-4.550741000	24.119612000	20.551481000
1	-3.159468000	23.578387000	21.734932000
1	-1.385055000	25.368339000	20.825459000
1	-3.148642000	27.065079000	20.975532000
1	-3.999957000	26.136267000	22.229384000
6	-1.793710000	20.726255000	19.072973000
6	-2.899195000	19.695520000	18.985275000
8	-3.581853000	19.550642000	17.934178000
6	-0.370199000	20.126468000	19.201987000
6	0.640788000	21.288835000	19.266690000
6	-0.066202000	19.133748000	18.070152000
6	2.082513000	20.864433000	19.556077000
1	-1.862186000	21.351543000	18.180067000
1	-0.316907000	19.575041000	20.152285000
1	0.602684000	21.842700000	18.317678000
1	0.313475000	21.999743000	20.039546000
1	0.920583000	18.678487000	18.196160000
1	-0.093026000	19.633033000	17.092490000
1	-0.790071000	18.311435000	18.057632000
1	2.723201000	21.744004000	19.684090000
1	2.145440000	20.259965000	20.469289000
1	2.503059000	20.269438000	18.739702000
7	-3.119277000	18.954946000	20.082223000
6	-4.014712000	17.793831000	20.140382000
6	-3.492404000	16.704435000	19.157377000
8	-2.334176000	16.312689000	19.240192000
6	-5.525704000	18.143945000	20.061197000
8	-5.965043000	18.450341000	18.729225000
6	-5.920923000	19.256894000	21.031196000
1	-2.528811000	19.097158000	20.907336000
1	-3.847919000	17.373848000	21.136961000
1	-6.068021000	17.229189000	20.334227000
1	-5.267238000	19.022605000	18.335758000
1	-6.999492000	19.426105000	20.964952000
1	-5.413604000	20.194813000	20.785168000
1	-5.664617000	18.991359000	22.064101000
7	-4.394139000	16.242025000	18.246887000
6	-4.005092000	15.407015000	17.127394000
6	-4.223735000	16.072409000	15.760379000
8	-4.425957000	15.394515000	14.760426000
1	-5.253339000	16.783837000	18.169227000
1	-2.943815000	15.166761000	17.249070000
7	-4.190212000	17.442924000	15.757362000
6	-4.283148000	18.199716000	14.523119000
6	-5.497037000	19.127764000	14.410898000
8	-5.523990000	20.021626000	13.562838000
1	-3.858655000	17.938305000	16.581811000
1	-3.391132000	18.814285000	14.370855000
1	-4.334758000	17.477349000	13.702249000
7	-6.534694000	18.874223000	15.250346000
6	-7.733151000	19.702326000	15.264429000
6	-7.632527000	20.868654000	16.265001000
8	-8.344753000	20.920072000	17.264995000
1	-6.382695000	18.231929000	16.017384000
1	-7.879013000	20.077228000	14.247126000
7	-6.676175000	21.779252000	15.956446000
6	-6.314477000	22.856443000	16.858128000
6	-5.905131000	24.078197000	16.023974000
8	-5.866878000	24.038427000	14.789330000
6	-5.168654000	22.439054000	17.798070000
8	-3.961697000	22.275098000	17.046739000
1	-6.199529000	21.738532000	15.061121000
1	-7.193077000	23.095800000	17.467276000
1	-5.007219000	23.176153000	18.591845000
1	-5.459758000	21.499818000	18.281448000
1	-3.753808000	21.322587000	17.031419000
7	-5.576818000	25.180628000	16.741746000
6	-5.149680000	26.395403000	16.084371000
6	-3.664554000	26.493115000	15.718049000
8	-3.196870000	27.577679000	15.362098000
1	-5.384633000	25.161657000	17.756170000
1	-5.704923000	26.511446000	15.148182000
7	-2.946651000	25.344750000	15.783325000

6	-1.577188000	25.246005000	15.296527000
6	-1.497103000	24.641427000	13.877521000
8	-0.490803000	24.831844000	13.192476000
6	-0.687288000	24.553312000	16.373323000
8	-0.320904000	25.493155000	17.375027000
6	0.606011000	23.947824000	15.837941000
1	-3.364688000	24.529094000	16.221854000
1	-1.203485000	26.265637000	15.178481000
1	-1.293508000	23.744935000	16.817295000
1	-1.094611000	26.034659000	17.683838000
1	1.206567000	23.606103000	16.687193000
1	1.180403000	24.683647000	15.268678000
1	0.407921000	23.093745000	15.184460000
7	-2.570032000	23.922247000	13.451715000
6	-2.702342000	23.458834000	12.074530000
6	-2.852182000	21.939241000	11.913321000
6	-1.638123000	21.064632000	12.192070000
6	-1.807480000	19.670464000	12.149211000
6	-0.356594000	21.564530000	12.458192000
6	-0.737918000	18.802751000	12.371367000
6	0.714661000	20.691889000	12.685847000
6	0.537508000	19.308202000	12.645914000
1	-3.408757000	23.918360000	14.021974000
1	-1.831070000	23.833854000	11.532896000
1	-3.693050000	21.588849000	12.526818000
1	-3.169380000	21.767918000	10.872644000
1	-2.797467000	19.268872000	11.940441000
1	-0.182183000	22.635663000	12.504056000
1	-0.898086000	17.727400000	12.334317000
1	1.697733000	21.104090000	12.901090000
1	1.368788000	18.632815000	12.837456000
1	8.014852000	26.629603000	24.699489000
1	5.625538000	25.398177000	25.062807000
1	9.515694000	29.982655000	18.249520000
1	10.272222000	29.261560000	20.753793000
1	2.919049000	27.821900000	15.608600000
1	5.272062000	29.139865000	15.269023000
1	1.375347000	24.559090000	22.073026000
1	0.712885000	25.163429000	19.490396000
1	7.831137000	17.616833000	21.182425000
1	4.034712000	15.629138000	22.309672000
1	0.249998000	12.281498000	20.082933000
1	-1.887243000	16.456568000	22.374354000
1	-2.588113000	21.471880000	22.473716000
1	-1.540935000	25.883653000	22.501625000
1	-3.592166000	23.932789000	11.639195000
1	-1.994216000	21.383453000	19.929499000
1	12.089507000	21.670478000	18.753875000
1	-5.378887000	27.247136000	16.729126000
1	-8.589977000	19.093086000	15.558698000
8	-2.209204000	27.263225000	18.303527000
1	-2.455058000	27.737460000	17.490551000
1	-3.046535000	26.825686000	18.612741000
1	-4.562502000	14.464966000	17.110706000
6	4.717514000	17.963691000	18.586427000
8	6.221110000	25.725059000	19.581913000
6	10.245257000	25.251964000	19.091701000
1	10.345082000	25.531218000	20.144873000
1	10.532590000	26.128120000	18.499448000
1	9.196526000	25.017707000	18.888955000
1	3.694683000	28.753017000	21.909191000

RE[4]

6	2.735608000	26.641516000	18.209257000
6	6.941482000	28.953093000	17.611260000
6	8.236055000	27.768898000	22.118283000
6	3.986212000	25.544592000	22.744821000
6	3.784695000	27.365966000	17.647435000
6	3.773430000	27.896362000	16.305083000
6	4.956717000	28.547566000	16.135303000
6	5.684466000	28.411038000	17.374408000
6	7.664222000	28.840731000	18.793389000
6	8.978496000	29.392778000	19.005159000
6	9.348444000	29.047940000	20.270626000

6	8.255281000	28.293513000	20.830010000
6	7.178831000	27.083628000	22.694022000
6	7.165821000	26.580884000	24.045031000
6	5.967648000	25.960310000	24.223673000
6	5.247282000	26.077085000	22.980046000
6	3.278938000	25.642425000	21.552824000
6	1.958463000	25.090261000	21.341330000
6	1.618166000	25.394587000	20.059876000
6	2.725036000	26.136989000	19.501219000
7	4.953873000	27.687477000	18.279467000
7	7.238416000	28.180404000	19.917862000
7	5.993338000	26.777322000	22.062151000
7	3.729095000	26.281250000	20.425807000
26	5.535222000	27.106054000	20.123615000
1	1.848641000	26.455232000	17.607979000
1	7.402894000	29.508146000	16.799413000
1	9.117910000	27.928231000	22.731960000
1	3.508432000	25.008325000	23.559144000
6	5.955206000	19.698185000	17.885265000
6	6.702475000	20.684616000	17.217988000
6	5.990143000	21.053961000	19.949074000
6	5.618093000	19.898033000	19.255116000
6	5.367053000	18.443467000	17.475759000
6	7.060082000	21.832038000	17.914325000
6	6.709263000	22.028894000	19.264210000
7	4.898336000	18.792443000	19.670644000
1	4.265324000	18.746020000	20.475222000
6	5.438915000	17.846738000	16.095990000
6	4.818749000	16.445607000	15.932490000
7	3.370850000	16.389850000	16.064885000
6	2.549048000	16.687741000	15.027176000
6	1.061965000	16.570148000	15.331359000
8	2.962244000	17.017013000	13.909714000
1	2.958814000	16.147179000	16.966727000
8	7.809551000	22.813310000	17.255142000
6	7.026460000	23.915420000	16.771158000
1	4.072498000	17.090321000	18.698186000
1	6.986475000	20.580069000	16.174450000
1	5.710834000	21.200846000	20.989207000
1	6.990115000	22.956814000	19.752976000
1	4.955981000	18.510507000	15.365896000
1	6.492561000	17.776062000	15.780762000
1	5.246947000	15.758780000	16.671871000
1	5.072658000	16.075809000	14.933895000
1	0.580733000	17.531686000	15.126715000
1	0.860524000	16.278176000	16.365422000
1	0.618938000	15.835105000	14.649926000
1	7.717681000	24.572253000	16.235484000
1	6.561071000	24.464231000	17.596996000
1	6.253623000	23.555949000	16.080231000
16	4.684698000	29.294602000	21.148596000
6	11.179979000	24.124581000	18.775323000
8	12.368119000	24.177866000	19.099630000
7	10.637623000	23.054629000	18.134263000
6	11.427996000	21.862129000	17.861465000
6	12.335169000	21.937877000	16.617004000
6	11.668915000	21.688990000	15.249157000
6	10.597069000	22.727790000	14.883488000
6	12.741966000	21.618684000	14.150001000
1	9.646059000	23.048889000	17.904137000
1	10.733440000	21.018009000	17.776568000
1	13.125278000	21.183033000	16.746873000
1	12.840749000	22.912170000	16.622612000
1	11.177011000	20.704159000	15.295839000
1	10.217573000	22.547267000	13.869319000
1	11.013473000	23.743584000	14.908397000
1	9.743024000	22.695867000	15.565292000
1	12.296976000	21.386216000	13.174481000
1	13.493983000	20.850170000	14.369531000
1	13.267036000	22.578905000	14.055211000
6	7.993618000	17.699475000	22.259213000
6	6.844086000	17.043401000	23.030203000
8	6.970949000	16.675887000	24.198854000
6	9.366772000	17.154236000	22.673330000
6	9.620787000	15.669343000	22.344468000
6	9.582266000	15.379203000	20.836003000
6	10.960941000	15.218196000	22.945210000
1	7.957145000	18.774281000	22.486015000
1	9.463495000	17.290346000	23.756628000
1	10.147331000	17.766207000	22.198213000
1	8.824946000	15.086734000	22.828506000
1	9.787496000	14.320037000	20.635974000
1	8.606671000	15.611459000	20.396252000
1	10.338459000	15.970857000	20.302004000
1	11.142898000	14.152185000	22.758272000
1	10.984666000	15.378842000	24.029844000
1	11.797493000	15.779155000	22.506040000
7	5.683354000	16.921913000	22.327201000
6	4.438774000	16.447189000	22.912621000
6	3.355039000	17.540311000	23.002398000
8	3.007493000	18.018171000	21.709052000
6	3.772117000	18.735983000	23.852827000
1	5.644482000	17.297235000	21.386200000
1	4.684968000	16.060680000	23.905286000
1	2.471030000	17.067320000	23.462589000
1	2.493245000	17.285590000	21.251843000
1	2.957042000	19.466816000	23.891277000
1	4.646476000	19.231789000	23.417909000
1	4.029452000	18.426106000	24.871870000
6	0.454019000	13.270479000	20.494702000
6	-0.724559000	13.589743000	21.416694000
8	-1.643324000	12.793190000	21.606820000
6	0.685856000	14.276195000	19.340208000
6	1.609678000	15.469867000	19.654558000
8	1.618474000	15.899318000	20.859548000
8	2.274250000	15.953046000	18.711615000
1	1.366630000	13.198153000	21.100605000
1	1.117960000	13.754024000	18.480690000
1	-0.274834000	14.690532000	19.005003000
7	-0.663612000	14.819337000	22.015698000
6	-1.825472000	15.405023000	22.667529000
6	-1.714583000	15.328431000	24.200777000
8	-0.438935000	15.866572000	24.607344000
6	-1.897637000	13.921339000	24.766184000
1	0.076324000	15.437091000	21.652690000
1	-2.723852000	14.879221000	22.328216000
1	-2.458128000	16.003971000	24.640702000
1	0.207498000	15.444386000	24.009131000
1	-1.741868000	13.927869000	25.850748000
1	-1.190270000	13.222746000	24.308566000
1	-2.905971000	13.548218000	24.553651000
6	-1.841753000	21.839584000	23.189725000
6	-1.430233000	23.281913000	22.877658000
8	-0.364868000	23.740978000	23.311340000
6	-0.611115000	20.920445000	23.186937000
6	-0.970399000	19.451133000	23.347360000
8	-1.835501000	18.911852000	22.638467000
7	-0.301009000	18.768527000	24.299579000
1	-2.310940000	21.821234000	24.183194000
1	0.091197000	21.257463000	23.954155000
1	-0.088390000	21.015919000	22.227160000
1	0.452300000	19.214242000	24.801824000
1	-0.391503000	17.748789000	24.377217000
7	-2.299029000	23.982737000	22.124808000
6	-2.055879000	25.357675000	21.701023000
6	-3.373124000	26.057398000	21.330956000
6	-4.180133000	25.287988000	20.259766000
8	-4.416629000	25.892658000	19.162975000
8	-4.544175000	24.120638000	20.548539000
1	-3.157218000	23.580202000	21.736626000
1	-1.379836000	25.371330000	20.835175000
1	-3.145167000	27.066803000	20.977771000
1	-4.000825000	26.137854000	22.228527000
6	-1.795127000	20.722895000	19.081902000
6	-2.900501000	19.692366000	18.992250000
8	-3.580968000	19.547282000	17.939728000
6	-0.371429000	20.122681000	19.207449000
6	0.639578000	21.285184000	19.269518000
6	-0.069981000	19.129718000	18.075158000
6	2.081949000	20.861165000	19.555989000

1	-1.864673000	21.350966000	18.191041000
1	-0.316074000	19.571387000	20.157697000
1	0.599233000	21.838638000	18.320372000
1	0.313769000	21.996387000	20.042739000
1	0.917168000	18.674665000	18.199092000
1	-0.099118000	19.628620000	17.097374000
1	-0.793737000	18.307284000	18.064647000
1	2.722852000	21.740824000	19.682122000
1	2.146863000	20.257170000	20.469361000
1	2.500779000	20.265825000	18.738993000
7	-3.122945000	18.952021000	20.088822000
6	-4.019475000	17.791662000	20.145146000
6	-3.496857000	16.702158000	19.162459000
8	-2.339169000	16.309139000	19.246873000
6	-5.529983000	18.143389000	20.063871000
8	-5.967076000	18.450845000	18.731431000
6	-5.925411000	19.256417000	21.033710000
1	-2.534261000	19.094488000	20.915249000
1	-3.854558000	17.370986000	21.141757000
1	-6.073673000	17.229099000	20.335742000
1	-5.267975000	19.022223000	18.339035000
1	-7.003774000	19.426511000	20.966339000
1	-5.417056000	20.193960000	20.788383000
1	-5.670482000	18.990482000	22.066856000
7	-4.397648000	16.241216000	18.250291000
6	-4.007895000	15.405464000	17.131658000
6	-4.221508000	16.071147000	15.764011000
8	-4.421806000	15.393408000	14.763580000
1	-5.256166000	16.783949000	18.171451000
1	-2.947460000	15.162787000	17.255827000
7	-4.186424000	17.441668000	15.760966000
6	-4.273391000	18.198038000	14.525979000
6	-5.485679000	19.127436000	14.408020000
8	-5.508666000	20.019674000	13.558149000
1	-3.856316000	17.936578000	16.586290000
1	-3.380032000	18.811501000	14.377148000
1	-4.322341000	17.475414000	13.705189000
7	-6.526354000	18.877031000	15.244700000
6	-7.723326000	19.707360000	15.253603000
6	-7.623940000	20.873958000	16.253950000
8	-8.338283000	20.926327000	17.252401000
1	-6.377713000	18.235720000	16.013231000
1	-7.864309000	20.081914000	14.235501000
7	-6.666214000	21.783717000	15.947243000
6	-6.304199000	22.859573000	16.850385000
6	-5.888688000	24.080438000	16.017992000
8	-5.846680000	24.041152000	14.783424000
6	-5.162564000	22.438358000	17.793743000
8	-3.954601000	22.269315000	17.045258000
1	-6.185972000	21.741273000	15.053947000
1	-7.183950000	23.101353000	17.456899000
1	-5.000507000	23.175270000	18.587634000
1	-5.458721000	21.500484000	18.276717000
1	-3.748090000	21.316412000	17.034635000
7	-5.560025000	25.181657000	16.737333000
6	-5.129782000	26.396647000	16.082402000
6	-3.643998000	26.492608000	15.718376000
8	-3.174400000	27.576716000	15.363628000
1	-5.371553000	25.161841000	17.752385000
1	-5.683439000	26.515191000	15.145574000
7	-2.927833000	25.343155000	15.783438000
6	-1.558465000	25.242451000	15.296842000
6	-1.479316000	24.639420000	13.877078000
8	-0.471823000	24.827208000	13.193045000
6	-0.670206000	24.546923000	16.373159000
8	-0.303725000	25.484712000	17.376758000
6	0.622920000	23.940980000	15.837945000
1	-3.347777000	24.527669000	16.220393000
1	-1.182882000	26.261557000	15.180124000
1	-1.277711000	23.738441000	16.815212000
1	-1.077372000	26.025944000	17.686121000
1	1.222142000	23.596720000	16.687114000
1	1.198757000	24.677394000	15.270895000
1	0.424590000	23.088613000	15.182336000
7	-2.554279000	23.924270000	13.449759000
6	-2.686915000	23.461901000	12.072301000
6	-2.841006000	21.942792000	11.910656000
6	-1.629589000	21.064762000	12.190142000
6	-1.803169000	19.671080000	12.148540000
6	-0.346534000	21.561104000	12.455513000
6	-0.736024000	18.800416000	12.370737000
6	0.722268000	20.685480000	12.683276000
6	0.541054000	19.302305000	12.644222000
1	-3.393385000	23.921927000	14.019502000
1	-1.814195000	23.834611000	11.531411000
1	-3.683155000	21.594618000	12.523616000
1	-3.157983000	21.772483000	10.869743000
1	-2.794587000	19.272391000	11.941079000
1	-0.169090000	22.631747000	12.501117000
1	-0.899337000	17.725501000	12.334619000
1	1.706618000	21.094941000	12.897934000
1	1.370437000	18.624586000	12.835737000
1	7.978535000	26.695831000	24.751145000
1	5.590905000	25.458195000	25.105691000
1	9.533412000	29.963187000	18.270915000
1	10.270043000	29.276412000	20.790780000
1	2.956463000	27.775152000	15.605658000
1	5.318129000	29.076999000	15.262702000
1	1.370929000	24.566391000	22.087789000
1	0.721320000	25.161515000	19.499634000
1	7.832819000	17.618330000	21.178380000
1	4.038130000	15.627070000	22.304888000
1	0.245868000	12.276542000	20.089161000
1	-1.885311000	16.457389000	22.375582000
1	-2.591295000	21.475087000	22.480261000
1	-1.543151000	25.886091000	22.510782000
1	-3.575042000	23.938613000	11.636512000
1	-1.994365000	21.377393000	19.940740000
1	12.063733000	21.687540000	18.736094000
1	-5.358540000	27.247645000	16.728262000
1	-8.582515000	19.099991000	15.544865000
8	-2.190824000	27.255750000	18.306767000
1	-2.433555000	27.732204000	17.494157000
1	-3.030442000	26.821374000	18.614017000
1	-4.567390000	14.464667000	17.113637000
6	4.717312000	17.952096000	18.581472000
8	6.210134000	25.719688000	19.592427000
6	10.218477000	25.269835000	19.065303000
1	10.323289000	25.556765000	20.115853000
1	10.501003000	26.142367000	18.465390000
1	9.169131000	25.032211000	18.869654000
1	3.673170000	28.767526000	21.874785000

TS1^{2}$_{C6}$

6	3.757582000	27.132835000	17.771144000
6	8.477556000	27.300010000	18.825825000
6	7.710630000	24.622692000	22.796287000
6	2.971826000	24.576212000	21.799138000
6	5.124003000	27.380565000	17.693748000
6	5.757591000	28.137132000	16.638136000
6	7.084707000	28.194397000	16.938848000
6	7.261198000	27.467570000	18.175726000
6	8.670918000	26.595393000	20.007393000
6	9.943693000	26.458793000	20.669934000
6	9.732671000	25.701188000	21.783302000
6	8.329382000	25.377137000	21.807060000
6	6.349446000	24.352395000	22.853606000
6	5.705715000	23.605540000	23.909334000
6	4.373325000	23.602489000	23.627694000
6	4.202397000	24.342440000	22.399465000
6	2.780824000	25.295787000	20.627684000
6	1.486021000	25.497769000	20.013611000
6	1.699402000	26.205161000	18.873014000
6	3.127591000	26.440947000	18.795254000
7	6.058916000	26.982521000	18.609966000
7	7.700488000	25.922690000	20.711627000
7	5.419164000	24.778222000	21.942190000
7	3.761504000	25.888911000	19.880096000
26	5.749235000	25.815806000	20.241935000

1	3.131536000	27.516169000	16.971175000
1	9.349861000	27.771843000	18.382021000
1	8.334631000	24.237372000	23.597257000
1	2.082585000	24.165747000	22.268499000
6	6.077011000	20.896628000	17.711683000
6	6.890843000	21.884628000	17.100265000
6	6.416612000	21.918278000	19.930433000
6	5.855566000	20.943828000	19.129564000
6	5.383960000	19.762933000	17.204532000
6	7.440499000	22.866104000	17.894366000
6	7.174293000	22.939804000	19.305937000
7	5.062160000	19.852828000	19.452135000
1	4.549688000	19.674551000	20.324556000
6	5.345026000	19.307268000	15.769058000
6	5.021083000	17.815620000	15.539270000
7	3.627807000	17.450669000	15.736583000
6	2.715512000	17.603914000	14.740208000
6	1.303779000	17.151963000	15.081082000
8	3.006381000	18.060611000	13.630146000
1	3.321756000	17.081398000	16.638200000
8	8.272702000	23.856896000	17.428393000
6	8.121839000	24.252047000	16.067661000
1	4.099689000	18.314469000	18.362615000
1	7.070752000	21.854529000	16.031250000
1	6.248801000	21.955450000	21.001645000
1	7.796572000	23.595253000	19.900233000
1	4.621902000	19.895950000	15.188799000
1	6.326292000	19.505786000	15.312718000
1	5.632326000	17.193245000	16.202885000
1	5.277494000	17.570823000	14.503872000
1	0.613144000	17.982605000	14.906546000
1	1.204357000	16.809660000	16.114354000
1	1.017718000	16.343563000	14.398713000
1	8.665938000	25.192558000	15.964222000
1	7.064315000	24.414917000	15.835438000
1	8.543332000	23.507335000	15.379600000
16	5.479277000	27.874264000	21.513038000
6	12.202945000	24.110051000	18.290946000
8	13.323541000	23.832382000	18.716578000
7	11.118100000	23.311828000	18.480962000
6	11.200837000	22.088736000	19.271801000
6	11.814077000	20.870227000	18.558339000
6	10.966101000	20.198952000	17.457323000
6	10.878353000	21.028730000	16.166303000
6	11.521298000	18.799438000	17.144949000
1	10.206517000	23.618374000	18.155847000
1	10.186404000	21.853736000	19.613242000
1	12.010216000	20.122540000	19.341424000
1	12.794115000	21.159944000	18.157791000
1	9.943794000	20.073933000	17.846255000
1	10.286908000	20.502084000	15.406802000
1	11.879170000	21.204802000	15.749134000
1	10.415410000	22.003115000	16.340548000
1	10.923656000	18.297637000	16.374156000
1	11.523707000	18.160158000	18.036484000
1	12.554664000	18.861783000	16.777167000
6	8.335819000	18.427480000	21.779422000
6	7.240105000	17.817882000	22.661857000
8	7.427461000	17.546521000	23.847142000
6	9.749889000	18.123314000	22.289084000
6	10.179460000	16.643574000	22.226125000
6	10.235075000	16.101692000	20.789405000
6	11.536855000	16.459662000	22.921637000
1	8.182157000	19.516152000	21.777345000
1	9.805384000	18.455680000	23.331832000
1	10.468156000	18.729931000	21.717891000
1	9.434499000	16.063935000	22.787110000
1	10.556793000	15.052823000	20.778274000
1	9.261022000	16.148494000	20.290523000
1	10.949501000	16.673684000	20.180864000
1	11.845142000	15.406509000	22.921475000
1	11.497578000	16.798916000	23.963554000
1	12.321916000	17.034989000	22.411760000
7	6.048940000	17.619916000	22.028652000
6	4.837961000	17.180321000	22.705272000
6	3.764331000	18.283869000	22.788487000
8	3.419407000	18.744233000	21.486738000
6	4.192107000	19.488367000	23.619438000
1	5.949358000	17.935760000	21.072482000
1	5.134495000	16.845828000	23.702745000
1	2.876253000	17.826607000	23.256644000
1	2.947248000	17.991217000	21.016970000
1	3.390701000	20.234308000	23.634184000
1	5.080299000	19.964594000	23.190986000
1	4.429499000	19.192682000	24.647426000
6	1.341288000	13.821673000	20.121950000
6	0.148835000	13.962183000	21.070684000
8	-0.657400000	13.049803000	21.247229000
6	1.431131000	14.888245000	19.003287000
6	2.210578000	16.171150000	19.348003000
8	2.200720000	16.554493000	20.566461000
8	2.787648000	16.767943000	18.408997000
1	2.266757000	13.835099000	20.712424000
1	1.905378000	14.451832000	18.118431000
1	0.421897000	15.196565000	18.697042000
7	0.064308000	15.169865000	21.709727000
6	-1.151629000	15.582452000	22.395861000
6	-1.008482000	15.471953000	23.923654000
8	0.183769000	16.172310000	24.336868000
6	-0.983486000	14.035939000	24.443295000
1	0.714124000	15.886891000	21.363022000
1	-1.980946000	14.957650000	22.048989000
1	-1.833291000	16.020978000	24.393635000
1	0.878865000	15.855910000	23.728712000
1	-0.816852000	14.030515000	25.526195000
1	-0.189382000	13.459708000	23.958177000
1	-1.930684000	13.529299000	24.226409000
6	-1.863759000	21.976798000	22.705563000
6	-1.558560000	23.383950000	22.172184000
8	-0.407886000	23.842384000	22.199174000
6	-0.577550000	21.201043000	23.002465000
6	-0.789042000	19.699703000	23.134382000
8	-1.557007000	19.071803000	22.389150000
7	-0.072102000	19.082230000	24.099161000
1	-2.479201000	22.063411000	23.610997000
1	-0.086425000	21.607492000	23.892607000
1	0.129154000	21.355237000	22.177744000
1	0.604192000	19.606111000	24.634534000
1	-0.031848000	18.058442000	24.149358000
7	-2.617461000	24.062355000	21.695382000
6	-2.508551000	25.406602000	21.130809000
6	-3.886900000	26.087168000	21.111692000
6	-4.914760000	25.275660000	20.293494000
8	-5.387815000	25.816550000	19.240842000
8	-5.196165000	24.124527000	20.714124000
1	-3.546868000	23.646077000	21.583863000
1	-2.099367000	25.364405000	20.111545000
1	-3.774675000	27.093668000	20.700896000
1	-4.258310000	26.170482000	22.141690000
6	-1.907101000	20.870518000	18.780427000
6	-2.914297000	19.738365000	18.787442000
8	-3.661724000	19.518147000	17.796883000
6	-0.433037000	20.421907000	18.939264000
6	0.461215000	21.676802000	18.990088000
6	-0.028502000	19.443474000	17.826899000
6	1.941471000	21.390166000	19.256445000
1	-2.034368000	21.409650000	17.839001000
1	-0.331926000	19.897497000	19.900766000
1	0.358404000	22.224664000	18.042935000
1	0.084483000	22.350817000	19.771200000
1	0.995548000	19.084782000	17.966257000
1	-0.093038000	19.924736000	16.841852000
1	-0.673436000	18.557765000	17.821442000
1	2.493578000	22.325128000	19.399614000
1	2.075819000	20.776785000	20.156827000
1	2.408634000	20.857007000	18.422483000
7	-2.979708000	18.993232000	19.904763000
6	-3.706004000	17.721948000	20.020961000
6	-3.085799000	16.703557000	19.018238000
8	-1.880200000	16.480911000	19.038355000

6	-5.253134000	17.853670000	20.020109000
8	-5.795933000	18.091232000	18.712996000
6	-5.752483000	18.900014000	21.015484000
1	-2.340842000	19.197045000	20.677670000
1	-3.431143000	17.345723000	21.010934000
1	-5.645327000	16.871703000	20.316006000
1	-5.207134000	18.760264000	18.291258000
1	-6.845262000	18.931939000	20.985650000
1	-5.375082000	19.896157000	20.765361000
1	-5.428622000	18.660459000	22.035663000
7	-3.958026000	16.109206000	18.157831000
6	-3.515710000	15.352680000	17.001936000
6	-3.963525000	15.974326000	15.669660000
8	-4.153801000	15.273025000	14.683375000
1	-4.888738000	16.520977000	18.128588000
1	-2.422466000	15.302652000	17.037915000
7	-4.146954000	17.331760000	15.687796000
6	-4.515885000	18.070201000	14.495741000
6	-5.854451000	18.813530000	14.574821000
8	-6.117910000	19.707666000	13.769211000
1	-3.817755000	17.874655000	16.482656000
1	-3.750526000	18.808479000	14.239444000
1	-4.575376000	17.350727000	13.672869000
7	-6.725318000	18.396584000	15.531295000
6	-8.018132000	19.036676000	15.729773000
6	-7.974546000	20.186646000	16.753673000
8	-8.607898000	20.135416000	17.804675000
1	-6.381477000	17.783741000	16.259662000
1	-8.347803000	19.408353000	14.754850000
7	-7.160860000	21.213491000	16.396461000
6	-6.953848000	22.367705000	17.252618000
6	-6.831858000	23.610804000	16.358547000
8	-6.877337000	23.523276000	15.126027000
6	-5.696335000	22.204964000	18.127783000
8	-4.510387000	22.282681000	17.333183000
1	-6.832264000	21.289216000	15.437676000
1	-7.824124000	22.462499000	17.912257000
1	-5.641990000	22.977219000	18.901631000
1	-5.764489000	21.235299000	18.635027000
1	-4.250708000	21.374292000	17.094775000
7	-6.659831000	24.784454000	17.012017000
6	-6.473403000	26.015608000	16.275997000
6	-5.046579000	26.326075000	15.809799000
8	-4.794697000	27.437767000	15.336220000
1	-6.385766000	24.849522000	18.005667000
1	-7.096318000	25.996394000	15.376863000
7	-4.132434000	25.333979000	15.933340000
6	-2.776337000	25.449378000	15.411120000
6	-2.639971000	24.832241000	14.001645000
8	-1.698661000	25.160560000	13.278232000
6	-1.758583000	24.937225000	16.479439000
8	-1.487794000	25.951205000	17.433366000
6	-0.413644000	24.497016000	15.909010000
1	-4.376928000	24.496341000	16.454904000
1	-2.574472000	26.511912000	15.256445000
1	-2.228009000	24.067369000	16.971796000
1	-2.302025000	26.458157000	17.681935000
1	0.258927000	24.279420000	16.744485000
1	0.028499000	25.284889000	15.293571000
1	-0.513966000	23.597973000	15.293398000
7	-3.599414000	23.942866000	13.621988000
6	-3.723927000	23.474038000	12.245657000
6	-3.659365000	21.950443000	12.065771000
6	-2.312106000	21.264889000	12.245853000
6	-2.254505000	19.868986000	12.093902000
6	-1.121194000	21.945714000	12.531371000
6	-1.054085000	19.172106000	12.234211000
6	0.081935000	21.244394000	12.675546000
6	0.129085000	19.857040000	12.531530000
1	-4.407135000	23.825699000	14.223836000
1	-2.939967000	23.974874000	11.672850000
1	-4.398603000	21.474941000	12.725260000
1	-4.017100000	21.741626000	11.045681000
1	-3.169301000	19.327154000	11.859125000
1	-1.118487000	23.025533000	12.647505000
1	-1.040109000	18.091165000	12.112147000
1	0.991191000	21.794404000	12.906429000
1	1.064758000	19.316427000	12.657181000
1	6.220749000	23.160531000	24.751840000
1	3.563177000	23.154834000	24.189104000
1	10.871259000	26.892979000	20.318017000
1	10.450562000	25.385583000	22.530152000
1	5.235379000	28.567188000	15.792379000
1	7.881400000	28.683880000	16.391600000
1	0.561091000	25.127827000	20.435132000
1	0.960651000	26.526925000	18.148808000
1	8.203774000	18.108462000	20.738528000
1	4.409553000	16.331799000	22.159085000
1	1.245545000	12.825896000	19.680703000
1	-1.348316000	16.627242000	22.138648000
1	-2.472939000	21.426201000	21.980608000
1	-1.800503000	25.980271000	21.737143000
1	-4.691178000	23.817268000	11.854834000
1	-2.170088000	21.576934000	19.578683000
1	11.808876000	22.308238000	20.156469000
1	-6.794292000	26.852775000	16.900472000
1	-8.733057000	18.300017000	16.101633000
8	-3.593666000	27.601105000	18.169924000
1	-3.946540000	27.914199000	17.319054000
1	-4.304319000	27.017928000	18.548233000
1	-3.899186000	14.327460000	17.014608000
6	4.762815000	19.173049000	18.304659000
8	5.790800000	24.455002000	19.258714000
6	11.944197000	25.368962000	17.469237000
1	12.199599000	25.177955000	16.419637000
1	10.903760000	25.705020000	17.519246000
1	12.602352000	26.162677000	17.831625000
1	4.392135000	28.353214000	20.869153000

TS1^{4}$_{C6}$

6	3.582118000	27.189307000	17.766084000
6	8.343231000	27.544556000	18.575604000
6	7.838540000	24.956599000	22.639764000
6	3.084898000	24.563214000	21.804325000
6	4.932065000	27.482109000	17.618603000
6	5.486162000	28.245490000	16.524431000
6	6.824719000	28.342067000	16.751430000
6	7.091728000	27.637310000	17.984490000
6	8.620573000	26.891521000	19.774160000
6	9.922819000	26.826988000	20.392979000
6	9.778834000	26.098785000	21.536519000
6	8.388080000	25.724853000	21.618794000
6	6.501201000	24.597293000	22.750430000
6	5.956538000	23.798755000	23.824956000
6	4.619453000	23.682743000	23.588888000
6	4.347522000	24.407912000	22.369542000
6	2.820427000	25.278508000	20.647175000
6	1.494616000	25.437847000	20.094898000
6	1.625940000	26.171803000	18.957651000
6	3.035664000	26.467720000	18.816565000
7	5.925241000	27.115577000	18.485405000
7	7.706298000	26.219463000	20.535842000
7	5.503037000	24.945930000	21.877343000
7	3.745768000	25.923248000	19.858906000
26	5.712055000	25.992585000	20.137185000
1	2.900634000	27.551041000	17.002633000
1	9.170576000	28.037816000	18.072950000
1	8.511045000	24.616541000	23.422048000
1	2.234381000	24.102798000	22.298190000
6	6.131569000	20.973432000	17.812930000
6	6.976689000	21.942494000	17.208588000
6	6.414368000	22.035678000	20.022997000
6	5.872059000	21.051034000	19.222778000
6	5.451884000	19.830716000	17.308103000
6	7.506839000	22.936381000	17.993525000
6	7.172078000	23.074322000	19.399544000
7	5.078494000	19.959434000	19.546205000
1	4.533186000	19.807645000	20.401090000
6	5.448951000	19.355115000	15.878406000

6	5.124850000	17.862530000	15.657308000
7	3.726728000	17.502814000	15.828461000
6	2.834000000	17.649868000	14.813850000
6	1.416317000	17.198532000	15.130750000
8	3.145039000	18.099629000	13.706297000
1	3.401273000	17.143159000	16.727242000
8	8.367096000	23.907914000	17.534504000
6	8.264418000	24.275236000	16.161913000
1	4.140599000	18.400106000	18.457158000
1	7.201408000	21.880784000	16.149144000
1	6.231001000	22.079809000	21.091271000
1	7.856334000	23.653796000	20.006961000
1	4.743869000	19.939710000	15.272367000
1	6.442673000	19.543790000	15.445263000
1	5.719549000	17.245302000	16.340414000
1	5.402928000	17.606132000	14.630243000
1	0.728492000	18.028797000	14.943805000
1	1.298962000	16.857094000	16.162363000
1	1.142392000	16.389713000	14.443895000
1	8.820620000	25.208560000	16.055972000
1	7.216175000	24.440647000	15.892025000
1	8.700979000	23.513034000	15.502910000
16	5.499093000	27.962946000	21.495956000
6	12.280417000	24.139212000	18.442055000
8	13.394702000	23.861332000	18.884348000
7	11.189484000	23.349104000	18.630196000
6	11.261092000	22.127568000	19.424947000
6	11.913896000	20.917881000	18.731401000
6	11.102294000	20.221682000	17.619080000
6	10.980981000	21.058432000	16.335553000
6	11.721912000	18.851861000	17.295880000
1	10.282305000	23.657447000	18.293930000
1	10.238745000	21.879290000	19.731599000
1	12.111003000	20.178154000	19.521655000
1	12.894800000	21.227190000	18.348577000
1	10.085354000	20.046019000	18.002490000
1	10.423390000	20.508578000	15.566933000
1	11.973948000	21.290980000	15.927477000
1	10.465518000	22.004954000	16.514897000
1	11.146818000	18.328139000	16.522575000
1	11.756401000	18.207283000	18.182957000
1	12.750658000	18.964652000	16.927128000
6	8.322544000	18.418918000	22.018106000
6	7.191241000	17.773073000	22.822718000
8	7.377650000	17.323199000	23.953048000
6	9.657737000	17.681156000	22.193992000
6	9.691430000	16.230613000	21.671871000
6	9.416069000	16.133094000	20.163609000
6	11.038537000	15.578159000	22.018149000
1	8.435173000	19.444570000	22.396134000
1	9.890203000	17.670117000	23.265072000
1	10.449099000	18.261472000	21.696770000
1	8.905411000	15.674177000	22.201180000
1	9.476378000	15.092528000	19.821668000
1	8.419789000	16.504557000	19.901600000
1	10.151413000	16.713954000	19.589878000
1	11.067086000	14.529430000	21.696775000
1	11.227736000	15.604855000	23.098083000
1	11.867566000	16.100123000	21.520708000
7	5.980844000	17.754515000	22.198245000
6	4.754308000	17.286054000	22.824557000
6	3.667958000	18.377977000	22.868531000
8	3.372262000	18.837096000	21.554274000
6	4.051270000	19.585353000	23.717661000
1	5.896285000	18.164797000	21.277229000
1	5.020190000	16.947826000	23.829464000
1	2.766249000	17.912169000	23.299705000
1	2.916792000	18.081397000	21.072628000
1	3.243099000	20.324214000	23.698050000
1	4.953945000	20.067623000	23.327978000
1	4.243844000	19.290522000	24.755635000
6	1.419925000	13.869384000	20.172085000
6	0.209983000	13.974096000	21.102781000
8	-0.575378000	13.040657000	21.262491000
6	1.493125000	14.938590000	19.054499000
6	2.230148000	16.243566000	19.409810000
8	2.185991000	16.630774000	20.626220000
8	2.808451000	16.853685000	18.480333000
1	2.335943000	13.911072000	20.775770000
1	1.991606000	14.516324000	18.176207000
1	0.479401000	15.217386000	18.735098000
7	0.084452000	15.176131000	21.746418000
6	-1.154625000	15.554285000	22.411217000
6	-1.041273000	15.428530000	23.940582000
8	0.132648000	16.140341000	24.385148000
6	-1.007014000	13.987471000	24.445637000
1	0.718396000	15.912412000	21.409570000
1	-1.963703000	14.917305000	22.039528000
1	-1.883128000	15.960869000	24.399494000
1	0.842401000	15.841112000	23.784940000
1	-0.862663000	13.972888000	25.531679000
1	-0.195764000	13.426872000	23.970869000
1	-1.942700000	13.470656000	24.204043000
6	-1.881114000	21.945113000	22.730028000
6	-1.569006000	23.350334000	22.196621000
8	-0.419791000	23.811199000	22.245356000
6	-0.598396000	21.160608000	23.019910000
6	-0.823819000	19.662487000	23.162827000
8	-1.590087000	19.035787000	22.414924000
7	-0.123363000	19.046966000	24.140995000
1	-2.489691000	22.035926000	23.639820000
1	-0.096064000	21.569247000	23.902601000
1	0.102108000	21.301890000	22.187649000
1	0.552462000	19.569534000	24.678253000
1	-0.090936000	18.023133000	24.199090000
7	-2.620696000	24.023063000	21.696423000
6	-2.509541000	25.366786000	21.131339000
6	-3.889007000	26.044957000	21.099734000
6	-4.910993000	25.231176000	20.275820000
8	-5.386642000	25.774424000	19.225615000
8	-5.187204000	24.076849000	20.691144000
1	-3.547782000	23.604477000	21.573672000
1	-2.091906000	25.324444000	20.115544000
1	-3.775276000	27.050996000	20.688233000
1	-4.268570000	26.129818000	22.126694000
6	-1.918723000	20.848747000	18.802167000
6	-2.916330000	19.708464000	18.798728000
8	-3.654650000	19.485440000	17.801765000
6	-0.441359000	20.411721000	18.962233000
6	0.441431000	21.674616000	19.011830000
6	-0.026446000	19.436090000	17.851401000
6	1.922772000	21.402066000	19.285583000
1	-2.047810000	21.392829000	17.863931000
1	-0.336422000	19.888925000	19.924258000
1	0.337131000	22.217459000	18.061947000
1	0.055539000	22.348132000	19.788881000
1	0.999668000	19.085089000	17.994537000
1	-0.091581000	19.916523000	16.865983000
1	-0.664703000	18.545546000	17.844355000
1	2.466596000	22.342914000	19.422283000
1	2.058108000	20.798391000	20.192153000
1	2.397676000	20.864873000	18.458688000
7	-2.983818000	18.959112000	19.912918000
6	-3.702666000	17.682784000	20.020808000
6	-3.067787000	16.670435000	19.021198000
8	-1.861431000	16.453727000	19.052295000
6	-5.250551000	17.804512000	20.008098000
8	-5.785126000	18.040593000	18.697432000
6	-5.764269000	18.846303000	21.000997000
1	-2.352681000	19.165431000	20.691514000
1	-3.433101000	17.306634000	21.012225000
1	-5.638517000	16.819535000	20.299533000
1	-5.198339000	18.715276000	18.282075000
1	-6.857056000	18.869777000	20.964343000
1	-5.393166000	19.845514000	20.753575000
1	-5.444804000	18.608864000	22.023054000
7	-3.928613000	16.074355000	18.150334000
6	-3.470657000	15.324553000	16.996119000
6	-3.906065000	15.950018000	15.661474000
8	-4.081463000	15.252367000	14.669859000

1	-4.861588000	16.480492000	18.113946000
1	-2.377675000	15.278725000	17.043985000
7	-4.096306000	17.306462000	15.683609000
6	-4.453991000	18.048970000	14.490735000
6	-5.796141000	18.786982000	14.558328000
8	-6.053057000	19.686143000	13.756101000
1	-3.780104000	17.847345000	16.485121000
1	-3.688264000	18.791076000	14.246630000
1	-4.501561000	17.332986000	13.664015000
7	-6.677526000	18.359581000	15.500359000
6	-7.976379000	18.991867000	15.685487000
6	-7.949873000	20.141822000	16.710137000
8	-8.587524000	20.083457000	17.758277000
1	-6.340572000	17.742527000	16.228363000
1	-8.298657000	19.361514000	14.707323000
7	-7.145454000	21.176941000	16.357136000
6	-6.946108000	22.328849000	17.217967000
6	-6.822079000	23.575036000	16.328515000
8	-6.865548000	23.492276000	15.095836000
6	-5.693183000	22.165577000	18.099386000
8	-4.504290000	22.242865000	17.308935000
1	-6.805843000	21.252438000	15.402249000
1	-7.820410000	22.419772000	17.872838000
1	-5.641990000	22.937205000	18.874087000
1	-5.763867000	21.195710000	18.605840000
1	-4.236164000	21.333395000	17.084258000
7	-6.650923000	24.746691000	16.986502000
6	-6.463899000	25.980062000	16.254485000
6	-5.035657000	26.295033000	15.795787000
8	-4.783989000	27.408903000	15.327217000
1	-6.380177000	24.808557000	17.981038000
1	-7.082539000	25.961305000	15.352420000
7	-4.119771000	25.304596000	15.920654000
6	-2.759838000	25.426945000	15.409951000
6	-2.606942000	24.809820000	14.002266000
8	-1.664051000	25.147670000	13.285421000
6	-1.748376000	24.920207000	16.486479000
8	-1.492708000	25.935275000	17.443789000
6	-0.395821000	24.489135000	15.927096000
1	-4.365281000	24.465302000	16.439029000
1	-2.562756000	26.490543000	15.256703000
1	-2.216390000	24.047088000	16.974307000
1	-2.311697000	26.438308000	17.685492000
1	0.271006000	24.273154000	16.767564000
1	0.047136000	25.280850000	15.317202000
1	-0.485514000	23.590767000	15.308947000
7	-3.553177000	23.908687000	13.617407000
6	-3.661087000	23.434634000	12.241362000
6	-3.575835000	21.911510000	12.065888000
6	-2.221727000	21.242992000	12.258893000
6	-2.146053000	19.847170000	12.113990000
6	-1.040973000	21.939384000	12.549071000
6	-0.938237000	19.165564000	12.265389000
6	0.169623000	21.253475000	12.704538000
6	0.234568000	19.866187000	12.567235000
1	-4.364309000	23.784770000	14.213128000
1	-2.879081000	23.944090000	11.673523000
1	-4.315082000	21.428610000	12.719950000
1	-3.922169000	21.696112000	11.043211000
1	-3.052264000	19.292840000	11.875061000
1	-1.051891000	23.019664000	12.660277000
1	-0.910468000	18.084331000	12.148643000
1	1.070564000	21.815537000	12.938861000
1	1.176070000	19.337816000	12.701433000
1	6.534802000	23.395983000	24.647519000
1	3.870738000	23.165694000	24.175468000
1	10.819930000	27.284642000	19.995124000
1	10.534090000	25.831284000	22.265103000
1	4.906814000	28.650822000	15.704232000
1	7.576175000	28.847561000	16.156880000
1	0.604581000	25.027705000	20.554285000
1	0.842962000	26.471521000	18.270792000
1	8.049431000	18.513751000	20.960962000
1	4.354321000	16.434576000	22.260451000
1	1.360840000	12.871248000	19.729771000
1	-1.366722000	16.597940000	22.162094000
1	-2.498683000	21.398676000	22.009149000
1	-1.807667000	25.942416000	21.742987000
1	-4.629506000	23.764643000	11.842225000
1	-2.189628000	21.548093000	19.604016000
1	11.836304000	22.353248000	20.330040000
1	-6.789597000	26.815250000	16.879178000
1	-8.690032000	18.250428000	16.050113000
8	-3.606423000	27.575728000	18.166269000
1	-3.956756000	27.887486000	17.313813000
1	-4.314883000	26.987064000	18.540666000
1	-3.850135000	14.297773000	17.000239000
6	4.804989000	19.257104000	18.400672000
8	5.994079000	24.542474000	19.265829000
6	12.039121000	25.386132000	17.597327000
1	12.700876000	26.180422000	17.951645000
1	12.301700000	25.173945000	16.553529000
1	11.001399000	25.731637000	17.632999000
1	4.268259000	27.688161000	21.983742000

IM1$^{2}_{C6}$

6	6.440845000	27.177957000	20.454796000
6	10.780520000	28.560678000	18.831598000
6	12.854221000	25.280653000	21.724707000
6	8.497041000	23.761191000	23.204268000
6	7.447680000	27.859915000	19.780128000
6	7.210668000	28.933607000	18.835976000
6	8.429070000	29.317991000	18.371144000
6	9.407583000	28.480789000	19.034805000
6	11.730573000	27.773314000	19.472669000
6	13.159138000	27.895656000	19.281681000
6	13.742550000	26.984754000	20.109788000
6	12.666319000	26.301258000	20.797010000
6	11.845836000	24.575565000	22.373375000
6	12.078734000	23.516252000	23.329099000
6	10.854479000	23.076600000	23.735520000
6	9.873346000	23.872282000	23.034603000
6	7.547965000	24.567589000	22.582492000
6	6.119645000	24.468735000	22.787963000
6	5.546587000	25.435065000	22.017377000
6	6.624244000	26.121612000	21.339600000
7	8.786110000	27.611102000	19.884524000
7	11.460395000	26.797249000	20.390982000
7	10.498100000	24.767086000	22.200238000
7	7.828054000	25.572610000	21.695240000
26	9.639594000	26.191130000	21.043548000
1	5.418074000	27.493232000	20.269437000
1	11.140030000	29.304470000	18.125939000
1	13.878581000	25.014034000	21.969786000
1	8.136560000	23.005155000	23.896098000
6	8.802793000	22.146399000	17.106352000
6	10.140385000	22.655339000	17.145924000
6	8.255669000	23.105440000	19.310371000
6	7.895304000	22.408956000	18.212438000
6	8.112442000	21.386283000	16.160336000
6	10.542565000	23.350391000	18.246970000
6	9.636176000	23.703291000	19.422711000
7	6.674778000	21.804470000	17.877608000
1	5.835772000	21.664541000	18.473091000
6	8.636033000	20.830179000	14.863911000
6	8.532343000	19.290328000	14.724043000
7	7.185646000	18.807911000	14.481851000
6	6.686169000	18.767492000	13.212987000
6	5.252286000	18.267231000	13.104629000
8	7.345036000	19.090813000	12.223830000
1	6.568314000	18.587183000	15.269719000
8	11.777230000	23.863475000	18.420865000
6	12.624344000	24.003201000	17.281987000
1	5.992599000	20.596615000	16.314566000
1	10.803100000	22.476618000	16.306814000
1	7.570158000	23.326919000	20.121368000
1	10.109498000	23.283330000	20.335110000
1	8.118551000	21.272129000	14.003544000
1	9.692302000	21.110753000	14.763452000

1	8.921802000	18.805666000	15.626721000
1	9.146384000	18.985100000	13.871708000
1	4.695290000	18.936604000	12.442333000
1	4.752820000	18.208678000	14.075320000
1	5.253152000	17.274932000	12.638511000
1	13.458670000	24.624043000	17.610558000
1	12.087463000	24.505684000	16.470209000
1	12.992954000	23.030281000	16.934482000
16	9.743293000	27.747946000	22.763703000
6	14.967979000	21.898736000	20.414115000
8	15.650312000	21.032628000	20.957201000
7	13.611823000	21.809350000	20.307318000
6	12.871007000	20.715267000	20.932216000
6	12.884025000	19.375570000	20.176115000
6	12.186137000	19.347531000	18.797474000
6	13.092018000	19.864545000	17.668130000
6	11.696823000	17.927626000	18.469015000
1	13.089179000	22.598706000	19.946803000
1	11.841447000	21.062445000	21.067967000
1	12.384732000	18.653555000	20.838586000
1	13.923371000	19.037331000	20.079983000
1	11.301600000	20.001067000	18.847079000
1	12.559846000	19.867412000	16.708518000
1	13.975939000	19.222171000	17.557554000
1	13.444936000	20.879811000	17.869670000
1	11.199709000	17.893821000	17.491661000
1	10.984522000	17.565566000	19.219922000
1	12.537549000	17.221337000	18.438626000
6	4.977127000	20.460160000	23.934946000
6	3.667574000	20.461594000	23.144465000
8	2.586697000	20.292017000	23.727684000
6	5.031875000	19.325258000	24.969711000
6	5.026620000	17.895997000	24.391455000
6	6.245231000	17.609325000	23.500659000
6	4.936737000	16.868212000	25.529796000
1	5.025651000	21.420612000	24.466242000
1	4.163643000	19.440191000	25.628553000
1	5.929177000	19.458141000	25.591783000
1	4.120829000	17.791046000	23.777374000
1	6.223280000	16.578034000	23.128559000
1	6.282968000	18.268067000	22.626231000
1	7.181069000	17.740881000	24.061149000
1	4.883961000	15.845136000	25.138180000
1	4.047993000	17.037827000	26.149144000
1	5.817364000	16.928729000	26.184081000
7	3.783494000	20.678527000	21.820971000
6	2.679108000	20.736841000	20.868798000
6	3.169484000	21.443530000	19.590568000
8	4.444511000	20.900328000	19.210648000
6	3.308608000	22.950712000	19.764828000
1	4.699397000	20.694127000	21.387980000
1	1.836851000	21.283410000	21.300055000
1	2.437901000	21.238871000	18.798484000
1	4.306283000	19.967779000	18.855656000
1	3.646405000	23.410864000	18.829322000
1	4.036972000	23.192215000	20.547892000
1	2.340800000	23.377904000	20.045536000
6	4.383359000	15.516721000	18.443793000
6	2.885391000	15.211350000	18.520935000
8	2.444513000	14.071196000	18.388948000
6	4.800597000	16.403506000	17.251886000
6	4.693970000	17.930623000	17.445288000
8	4.041331000	18.366059000	18.447556000
8	5.272847000	18.646451000	16.589412000
1	4.705753000	15.999477000	19.374259000
1	5.846997000	16.199570000	16.995763000
1	4.224893000	16.137587000	16.354939000
7	2.094246000	16.303765000	18.748177000
6	0.640327000	16.248540000	18.633032000
6	-0.055057000	16.223338000	20.006044000
8	0.458637000	17.305384000	20.806090000
6	0.098128000	14.892198000	20.740349000
1	2.573030000	17.207450000	18.701097000
1	0.371034000	15.358303000	18.057409000
1	-1.117391000	16.444088000	19.864257000
1	1.424826000	17.186363000	20.817573000
1	-0.368844000	14.955563000	21.729331000
1	1.154076000	14.626945000	20.864502000
1	-0.380484000	14.083601000	20.177272000
6	-1.583348000	22.815125000	22.913291000
6	-1.022809000	23.232531000	21.549021000
8	0.165374000	23.042946000	21.251789000
6	-0.824076000	21.634181000	23.518006000
6	-1.033118000	20.352654000	22.715115000
8	-2.102503000	20.161360000	22.109171000
7	-0.033950000	19.457948000	22.754634000
1	-1.502522000	23.679910000	23.585809000
1	-1.190563000	21.448947000	24.537238000
1	0.243492000	21.857547000	23.591125000
1	0.880252000	19.728482000	23.122374000
1	-0.078084000	18.641897000	22.143849000
7	-1.903969000	23.859695000	20.746265000
6	-1.569951000	24.353179000	19.416348000
6	-2.610296000	25.378657000	18.942875000
6	-4.062386000	24.842349000	18.928965000
8	-4.747630000	25.070327000	17.879820000
8	-4.480734000	24.245343000	19.952105000
1	-2.912914000	23.862142000	20.927606000
1	-1.521163000	23.520460000	18.700927000
1	-2.336342000	25.727794000	17.943236000
1	-2.587525000	26.246806000	19.617010000
6	-2.982945000	20.471825000	18.543295000
6	-4.003945000	19.510802000	19.107042000
8	-5.094562000	19.277860000	18.514069000
6	-1.785205000	19.745949000	17.865833000
6	-0.825673000	20.812625000	17.298058000
6	-2.268516000	18.754530000	16.796075000
6	0.462254000	20.259325000	16.675431000
1	-3.482526000	21.108454000	17.806268000
1	-1.249171000	19.182731000	18.644871000
1	-1.371027000	21.408054000	16.553988000
1	-0.554191000	21.498436000	18.112510000
1	-1.424763000	18.257587000	16.305330000
1	-2.850281000	19.274361000	16.025454000
1	-2.895537000	17.969260000	17.231405000
1	1.120674000	21.078781000	16.363664000
1	1.025787000	19.638212000	17.384244000
1	0.263340000	19.650711000	15.786338000
7	-3.681673000	18.894716000	20.252564000
6	-4.471465000	17.830746000	20.872507000
6	-4.585727000	16.644492000	19.870620000
8	-3.581959000	16.169100000	19.349577000
6	-5.791934000	18.321647000	21.529134000
8	-6.817170000	18.597231000	20.557597000
6	-5.579400000	19.525263000	22.444781000
1	-2.883960000	19.244027000	20.798265000
1	-3.841043000	17.457826000	21.687303000
1	-6.181060000	17.484988000	22.124656000
1	-6.367440000	19.036548000	19.800042000
1	-6.538492000	19.813824000	22.884317000
1	-5.182557000	20.378875000	21.888077000
1	-4.866878000	19.292970000	23.244599000
7	-5.846775000	16.182171000	19.643183000
6	-6.160189000	15.291189000	18.539896000
6	-7.195904000	15.890215000	17.572946000
8	-7.998224000	15.170655000	16.991239000
1	-6.592626000	16.793657000	19.966949000
1	-5.225903000	15.078280000	18.009021000
7	-7.154706000	17.253021000	17.442476000
6	-8.035381000	17.955752000	16.527024000
6	-9.188281000	18.745642000	17.156987000
8	-9.941975000	19.403772000	16.434910000
1	-6.356099000	17.763360000	17.812523000
1	-7.461167000	18.675769000	15.936231000
1	-8.468885000	17.231339000	15.833808000
7	-9.339641000	18.680836000	18.505482000
6	-10.292282000	19.547558000	19.194410000
6	-9.631765000	20.884471000	19.581198000
8	-9.383187000	21.164966000	20.751497000
1	-8.593691000	18.275817000	19.059412000

1	-11.139483000	19.700263000	18.520712000
7	-9.295932000	21.660855000	18.519257000
6	-8.265826000	22.681193000	18.619121000
6	-8.345969000	23.568476000	17.366250000
8	-9.193860000	23.373410000	16.489300000
6	-6.872678000	22.040555000	18.723376000
8	-6.597992000	21.418573000	17.464217000
1	-9.533866000	21.358805000	17.579158000
1	-8.441599000	23.278836000	19.519652000
1	-6.111948000	22.785851000	18.990041000
1	-6.907608000	21.298612000	19.530697000
1	-5.886376000	20.764018000	17.610254000
7	-7.408409000	24.543399000	17.292613000
6	-7.269303000	25.340387000	16.095708000
6	-6.458065000	24.715166000	14.957020000
8	-6.115768000	25.423948000	14.004957000
1	-6.567149000	24.565774000	17.887679000
1	-8.261242000	25.558115000	15.688112000
7	-6.196995000	23.383731000	15.030228000
6	-5.532672000	22.712844000	13.925546000
6	-6.537932000	22.045234000	12.961383000
8	-6.189387000	21.698797000	11.829184000
6	-4.452490000	21.703971000	14.431530000
8	-3.810720000	22.182330000	15.598324000
6	-3.442031000	21.377900000	13.327981000
1	-6.271002000	22.889571000	15.920522000
1	-5.040887000	23.488078000	13.330658000
1	-4.963186000	20.780506000	14.737387000
1	-3.579017000	23.141972000	15.487033000
1	-2.738530000	20.618715000	13.686191000
1	-2.869116000	22.278143000	13.072739000
1	-3.942018000	21.019581000	12.424198000
7	-7.792601000	21.859197000	13.448889000
6	-8.912217000	21.423221000	12.624092000
6	-9.558365000	20.098951000	13.059032000
6	-8.832732000	18.802930000	12.729715000
6	-9.417921000	17.596471000	13.152892000
6	-7.628323000	18.739274000	12.017117000
6	-8.827649000	16.365550000	12.871100000
6	-7.033961000	17.503480000	11.735551000
6	-7.627292000	16.314316000	12.155907000
1	-8.007647000	22.256197000	14.357879000
1	-8.550348000	21.376157000	11.594008000
1	-9.748161000	20.126468000	14.140046000
1	-10.552414000	20.060280000	12.587174000
1	-10.343313000	17.632687000	13.724395000
1	-7.132467000	19.649450000	11.691790000
1	-9.295908000	15.448483000	13.220607000
1	-6.094260000	17.479642000	11.188430000
1	-7.157872000	15.357490000	11.940186000
1	13.056789000	23.168158000	23.638057000
1	10.621042000	22.296529000	24.449979000
1	13.634526000	28.601154000	18.610734000
1	14.797214000	26.788993000	20.263406000
1	6.233459000	29.320289000	18.574144000
1	8.664285000	30.089826000	17.648402000
1	5.634176000	23.754718000	23.441695000
1	4.495841000	25.672491000	21.908173000
1	5.841780000	20.433409000	23.261521000
1	2.340650000	19.720976000	20.622499000
1	4.879232000	14.544854000	18.373372000
1	0.308472000	17.136553000	18.085339000
1	-2.645845000	22.570870000	22.822466000
1	-0.570602000	24.799248000	19.449911000
1	-9.684165000	22.202724000	12.667281000
1	-2.608900000	21.107314000	19.355213000
1	13.295448000	20.551004000	21.928948000
1	-6.783015000	26.283582000	16.352578000
1	-10.628336000	19.061703000	20.112284000
8	-3.394042000	24.915997000	15.516446000
1	-4.003267000	25.302718000	14.863194000
1	-3.866342000	25.042872000	16.385438000
1	-6.574598000	14.339789000	18.888506000
6	6.809300000	21.199222000	16.694853000
8	9.513643000	25.083328000	19.477797000
6	15.595333000	23.131940000	19.773133000
1	15.938395000	22.880932000	18.761929000
1	14.907267000	23.980524000	19.706738000
1	16.472762000	23.418856000	20.358034000
1	9.590825000	26.901990000	23.808863000

$IM1^{4}{}_{C6}$

6	6.685615000	27.489936000	20.398869000
6	11.313684000	28.416603000	19.155513000
6	12.802906000	24.773586000	21.975029000
6	8.155574000	23.805225000	23.171142000
6	7.820578000	28.079537000	19.822581000
6	7.796380000	29.209792000	18.910637000
6	9.088219000	29.468480000	18.562211000
6	9.917949000	28.499960000	19.257315000
6	12.165450000	27.500737000	19.787738000
6	13.615610000	27.471013000	19.695993000
6	14.041147000	26.450709000	20.499957000
6	12.848366000	25.850250000	21.077140000
6	11.663306000	24.188539000	22.545582000
6	11.684461000	23.089092000	23.488986000
6	10.387806000	22.815135000	23.817526000
6	9.554814000	23.745331000	23.083632000
6	7.299977000	24.712534000	22.530057000
6	5.851906000	24.783431000	22.653557000
6	5.442119000	25.822171000	21.867903000
6	6.636884000	26.389482000	21.263259000
7	9.115290000	27.688491000	20.006739000
7	11.752740000	26.506556000	20.618580000
7	10.358157000	24.549692000	22.311910000
7	7.724266000	25.686150000	21.681601000
26	9.731781000	26.112201000	21.136040000
1	5.732057000	27.944715000	20.142788000
1	11.788379000	29.156910000	18.515781000
1	13.756451000	24.349768000	22.280062000
1	7.689420000	23.080115000	23.833845000
6	8.759133000	22.253584000	17.040474000
6	10.102197000	22.748857000	17.072083000
6	8.272279000	23.109625000	19.300038000
6	7.879492000	22.471354000	18.179278000
6	8.039269000	21.543122000	16.078424000
6	10.535609000	23.391630000	18.193342000
6	9.657545000	23.697561000	19.402208000
7	6.645021000	21.895547000	17.845983000
1	5.816577000	21.742444000	18.454916000
6	8.524532000	21.032304000	14.749393000
6	8.445950000	19.493748000	14.578952000
7	7.098939000	18.988455000	14.392538000
6	6.544763000	18.947020000	13.146165000
6	5.117721000	18.419017000	13.096809000
8	7.153010000	19.290435000	12.131838000
1	6.521520000	18.748701000	15.204623000
8	11.779322000	23.878393000	18.369077000
6	12.624188000	24.026110000	17.230183000
1	5.915913000	20.769313000	16.244821000
1	10.745549000	22.597745000	16.212784000
1	7.610497000	23.299080000	20.138814000
1	10.153179000	23.244160000	20.285484000
1	7.963687000	21.482647000	13.921236000
1	9.569998000	21.337874000	14.614832000
1	8.889956000	18.998670000	15.450443000
1	9.022263000	19.220054000	13.690461000
1	4.519697000	19.080203000	12.462699000
1	4.661965000	18.345808000	14.087768000
1	5.118115000	17.429124000	12.625589000
1	13.481466000	24.605749000	17.574523000
1	12.101460000	24.572714000	16.437654000
1	12.956207000	23.052302000	16.849414000
16	9.895368000	27.627088000	22.928918000
6	14.948949000	21.632257000	20.238500000
8	15.598529000	20.706638000	20.719953000
7	13.585768000	21.638038000	20.209313000
6	12.806775000	20.576174000	20.843192000
6	12.687673000	19.263388000	20.050014000

6	11.920361000	19.326121000	18.710024000
6	12.799716000	19.820381000	17.549901000
6	11.322604000	17.952094000	18.366358000
1	13.099421000	22.468167000	19.892998000
1	11.812413000	20.986323000	21.048212000
1	12.173954000	18.556502000	20.717678000
1	13.694772000	18.858641000	19.889278000
1	11.084311000	20.032992000	18.825627000
1	12.220906000	19.886728000	16.620220000
1	13.633597000	19.127234000	17.375584000
1	13.226845000	20.804888000	17.760375000
1	10.775712000	17.982295000	17.415840000
1	10.626531000	17.612306000	19.142404000
1	12.112382000	17.194636000	18.271215000
6	4.967093000	20.709351000	23.963291000
6	3.664578000	20.651525000	23.163184000
8	2.585066000	20.456784000	23.741076000
6	5.049849000	19.599646000	25.023094000
6	5.099771000	18.158950000	24.476226000
6	6.339566000	17.894180000	23.608169000
6	5.028369000	17.153322000	25.635493000
1	4.980215000	21.682426000	24.473334000
1	4.171189000	19.698860000	25.670562000
1	5.935322000	19.776722000	25.651043000
1	4.206527000	18.010635000	23.852744000
1	6.356309000	16.855423000	23.257141000
1	6.368791000	18.536111000	22.721006000
1	7.262510000	18.067484000	24.178546000
1	5.015899000	16.120832000	25.265778000
1	4.125627000	17.305794000	26.238839000
1	5.896992000	17.257952000	26.300152000
7	3.783014000	20.849017000	21.836769000
6	2.682853000	20.851226000	20.877766000
6	3.162689000	21.530133000	19.581081000
8	4.447720000	20.997276000	19.219472000
6	3.272773000	23.044305000	19.710821000
1	4.701029000	20.878413000	21.409129000
1	1.825112000	21.389914000	21.288078000
1	2.437063000	21.288578000	18.793979000
1	4.322353000	20.059602000	18.871549000
1	3.601334000	23.482979000	18.761829000
1	3.995331000	23.325719000	20.485811000
1	2.296525000	23.460455000	19.978707000
6	4.447979000	15.619654000	18.424722000
6	2.953864000	15.300267000	18.519030000
8	2.520909000	14.158045000	18.378973000
6	4.841267000	16.518657000	17.233787000
6	4.717960000	18.043196000	17.437680000
8	4.079474000	18.462940000	18.456068000
8	5.269747000	18.772112000	16.575349000
1	4.778389000	16.098915000	19.354128000
1	5.887423000	16.329403000	16.965455000
1	4.259525000	16.250802000	16.341351000
7	2.156480000	16.383037000	18.770029000
6	0.701971000	16.317262000	18.669239000
6	0.020181000	16.273437000	20.048448000
8	0.530241000	17.352967000	20.854104000
6	0.192770000	14.937252000	20.769112000
1	2.627134000	17.291103000	18.725857000
1	0.433875000	15.430374000	18.087933000
1	-1.045478000	16.484871000	19.917505000
1	1.497455000	17.242705000	20.857800000
1	-0.265239000	14.987220000	21.763034000
1	1.252399000	14.681189000	20.880827000
1	-0.283420000	14.129284000	20.203112000
6	-1.625412000	22.865930000	22.872240000
6	-1.076393000	23.278057000	21.501629000
8	0.112046000	23.099529000	21.198967000
6	-0.841137000	21.709013000	23.490946000
6	-1.022401000	20.413339000	22.704165000
8	-2.085839000	20.193193000	22.097577000
7	-0.005936000	19.539217000	22.758946000
1	-1.562279000	23.740662000	23.533702000
1	-1.203958000	21.528519000	24.512369000
1	0.221424000	21.955555000	23.561856000
1	0.900545000	19.833132000	23.127809000
1	-0.031540000	18.713080000	22.160804000
7	-1.969199000	23.888897000	20.698951000
6	-1.647869000	24.378910000	19.364658000
6	-2.702615000	25.389840000	18.891545000
6	-4.147702000	24.834832000	18.886117000
8	-4.838447000	25.043713000	17.836605000
8	-4.555510000	24.241605000	19.915716000
1	-2.976944000	23.882280000	20.886538000
1	-1.593324000	23.543203000	18.653215000
1	-2.437107000	25.738134000	17.889353000
1	-2.687620000	26.260785000	19.562262000
6	-2.987015000	20.473068000	18.533867000
6	-3.990233000	19.498733000	19.106649000
8	-5.078175000	19.243211000	18.518007000
6	-1.778473000	19.762768000	17.859202000
6	-0.835944000	20.841368000	17.285617000
6	-2.246464000	18.757790000	16.795358000
6	0.459609000	20.304769000	16.664267000
1	-3.498626000	21.096187000	17.793532000
1	-1.233380000	19.212228000	18.641028000
1	-1.391007000	21.424899000	16.539279000
1	-0.574164000	21.534803000	18.096706000
1	-1.395121000	18.271384000	16.307223000
1	-2.836695000	19.263875000	16.022023000
1	-2.860819000	17.965231000	17.235524000
1	1.105639000	21.132714000	16.349043000
1	1.032579000	19.694863000	17.375262000
1	0.269289000	19.689668000	15.777757000
7	-3.654621000	18.896503000	20.255608000
6	-4.423581000	17.823281000	20.885784000
6	-4.516871000	16.626661000	19.894245000
8	-3.504652000	16.164567000	19.377657000
6	-5.751804000	18.295121000	21.540734000
8	-6.783664000	18.544135000	20.569116000
6	-5.559608000	19.509750000	22.446343000
1	-2.859638000	19.260994000	20.795168000
1	-3.784952000	17.469336000	21.702621000
1	-6.124482000	17.456318000	22.143684000
1	-6.343599000	18.986959000	19.807976000
1	-6.522768000	19.783771000	22.886286000
1	-5.180537000	20.366218000	21.881678000
1	-4.840646000	19.297340000	23.245924000
7	-5.769558000	16.140364000	19.670538000
6	-6.067093000	15.234491000	18.574969000
6	-7.113026000	15.806893000	17.602906000
8	-7.902076000	15.068310000	17.026914000
1	-6.526221000	16.740811000	19.989814000
1	-5.129097000	15.033429000	18.046041000
7	-7.096663000	17.169153000	17.461794000
6	-7.989961000	17.848714000	16.541126000
6	-9.156395000	18.623171000	17.165302000
8	-9.921478000	19.262653000	16.438446000
1	-6.307904000	17.696993000	17.828405000
1	-7.428807000	18.573915000	15.944125000
1	-8.410749000	17.111082000	15.854054000
7	-9.306165000	18.566290000	18.514291000
6	-10.273965000	19.420923000	19.197114000
6	-9.637991000	20.772672000	19.573490000
8	-9.394649000	21.066717000	20.741563000
1	-8.553699000	18.177586000	19.071085000
1	-11.124170000	19.553102000	18.522840000
7	-9.316970000	21.547086000	18.505625000
6	-8.307784000	22.588776000	18.597672000
6	-8.406093000	23.464905000	17.338290000
8	-9.249562000	23.245735000	16.462742000
6	-6.901847000	21.977229000	18.705877000
8	-6.614749000	21.352568000	17.450945000
1	-9.549591000	21.233915000	17.567837000
1	-8.495385000	23.189393000	19.493828000
1	-6.156398000	22.739684000	18.967262000
1	-6.921622000	21.240150000	19.518146000
1	-5.894445000	20.708805000	17.602236000
7	-7.488932000	24.458467000	17.257335000
6	-7.365948000	25.248896000	16.054316000

6	-6.542932000	24.631310000	14.919955000
8	-6.216399000	25.339297000	13.961745000
1	-6.648333000	24.502704000	17.852119000
1	-8.362090000	25.444040000	15.645523000
7	-6.253781000	23.306393000	15.003741000
6	-5.577416000	22.640447000	13.903368000
6	-6.570472000	21.947285000	12.944564000
8	-6.216465000	21.600722000	11.814107000
6	-4.478041000	21.656088000	14.415882000
8	-3.843057000	22.156380000	15.577203000
6	-3.463573000	21.339031000	13.313383000
1	-6.317288000	22.817602000	15.897801000
1	-5.100758000	23.420552000	13.302565000
1	-4.971175000	20.726108000	14.730740000
1	-3.631335000	23.119591000	15.457874000
1	-2.745335000	20.596522000	13.677229000
1	-2.908092000	22.247501000	13.048921000
1	-3.958125000	20.963169000	12.413753000
7	-7.820804000	21.739984000	13.434656000
6	-8.932607000	21.277449000	12.613761000
6	-9.552863000	19.943888000	13.057800000
6	-8.802433000	18.659985000	12.736639000
6	-9.364082000	17.445256000	13.167988000
6	-7.597334000	18.614882000	12.023790000
6	-8.750283000	16.224130000	12.893982000
6	-6.979280000	17.388973000	11.750139000
6	-7.549373000	16.191377000	12.178626000
1	-8.042686000	22.138327000	14.341418000
1	-8.570750000	21.230719000	11.583673000
1	-9.742717000	19.974868000	14.138714000
1	-10.546173000	19.882909000	12.586745000
1	-10.289834000	17.467387000	13.739631000
1	-7.119300000	19.532261000	11.692080000
1	-9.200659000	15.300504000	13.249669000
1	-6.039476000	17.379679000	11.202759000
1	-7.061620000	15.242397000	11.969015000
1	12.582134000	22.601677000	23.850033000
1	10.017792000	22.059997000	24.500717000
1	14.219985000	28.154136000	19.110380000
1	15.060299000	26.142336000	20.702540000
1	6.899620000	29.723020000	18.585202000
1	9.459589000	30.237287000	17.895183000
1	5.237964000	24.128573000	23.260072000
1	4.430701000	26.176569000	21.711029000
1	5.837630000	20.696770000	23.296968000
1	2.370394000	19.820381000	20.660776000
1	4.951625000	14.652877000	18.340583000
1	0.357341000	17.207618000	18.133295000
1	-2.682740000	22.598474000	22.786068000
1	-0.653557000	24.836604000	19.390660000
1	-9.719269000	22.042334000	12.652678000
1	-2.621999000	21.120321000	19.340551000
1	13.274368000	20.350976000	21.808420000
1	-6.898204000	26.203345000	16.303899000
1	-10.600696000	18.936066000	20.118867000
8	-3.484265000	24.898086000	15.472638000
1	-4.102731000	25.265988000	14.817200000
1	-3.957983000	25.021958000	16.341274000
1	-6.464797000	14.278999000	18.931766000
6	6.746441000	21.344787000	16.634593000
8	9.517413000	25.073671000	19.520031000
6	15.622161000	22.837824000	19.592330000
1	15.872975000	22.599664000	18.551304000
1	14.998192000	23.737097000	19.602487000
1	16.557715000	23.037247000	20.120687000
1	10.221135000	26.737669000	23.894985000

IM2^{2}$_{C6}$

6	3.683668000	27.726092000	18.511008000
6	7.956126000	25.719565000	19.657896000
6	5.962643000	24.056974000	23.790379000
6	1.650062000	25.863901000	22.479346000
6	5.002163000	27.296676000	18.458060000
6	5.910057000	27.594312000	17.373408000
6	7.095074000	27.000728000	17.681866000
6	6.911105000	26.346335000	18.959436000
6	7.850563000	25.125123000	20.892865000
6	8.887300000	24.742332000	21.777075000
6	8.320256000	24.293696000	22.951684000
6	6.909218000	24.362278000	22.841398000
6	4.589310000	24.351343000	23.735392000
6	3.651326000	23.949098000	24.761906000
6	2.438915000	24.459525000	24.404747000
6	2.643231000	25.175493000	23.164906000
6	1.841527000	26.568927000	21.294468000
6	0.787597000	27.270191000	20.589616000
6	1.363698000	27.812875000	19.484099000
6	2.762064000	27.429613000	19.511926000
7	5.635372000	26.533985000	19.405571000
7	6.583239000	24.811526000	21.508441000
7	3.955277000	25.086581000	22.780271000
7	3.024226000	26.687048000	20.624639000
26	4.759718000	25.843747000	21.076983000
1	3.327401000	28.325863000	17.678316000
1	8.946971000	25.742283000	19.214722000
1	6.321395000	23.587510000	24.702350000
1	0.636476000	25.842414000	22.870954000
6	6.038129000	21.216878000	18.133833000
6	5.478852000	22.300129000	17.417316000
6	5.305799000	22.239527000	20.253420000
6	5.940587000	21.205003000	19.546519000
6	6.684305000	19.989325000	17.736826000
6	4.851531000	23.328294000	18.103578000
6	4.747678000	23.322118000	19.547690000
7	6.524043000	20.041185000	19.999688000
1	6.415201000	19.617854000	20.926671000
6	6.881312000	19.492903000	16.327878000
6	6.763466000	17.961207000	16.172368000
7	5.506178000	17.411973000	16.658195000
6	4.345287000	17.610238000	15.982022000
6	3.086610000	17.086286000	16.656441000
8	4.299493000	18.173775000	14.884347000
1	5.497433000	16.983768000	17.582883000
8	4.234281000	24.392172000	17.499078000
6	4.144425000	24.386124000	16.088397000
1	7.330844000	18.312108000	19.061037000
1	5.517632000	22.302524000	16.333269000
1	5.137609000	22.171423000	21.326047000
1	6.218203000	23.984704000	20.988869000
1	6.123066000	19.944939000	15.678802000
1	7.857312000	19.793784000	15.912034000
1	7.569083000	17.455938000	16.715754000
1	6.866794000	17.709707000	15.111230000
1	2.391931000	17.921038000	16.796056000
1	3.272927000	16.617248000	17.625966000
1	2.597915000	16.364239000	15.994020000
1	3.583367000	25.283830000	15.820036000
1	3.609559000	23.500314000	15.719402000
1	5.137499000	24.427838000	15.616775000
16	5.423097000	27.725018000	22.257486000
6	9.878303000	22.863140000	18.466744000
8	10.568837000	23.721760000	19.025747000
7	9.677211000	21.624673000	18.991242000
6	10.152811000	21.270605000	20.327442000
6	11.656621000	20.955531000	20.425551000
6	12.121233000	19.570866000	19.930954000
6	11.939713000	19.372380000	18.417250000
6	13.592558000	19.347254000	20.317342000
1	8.965580000	21.033370000	18.572535000
1	9.559103000	20.414351000	20.660945000
1	11.924782000	21.038543000	21.489069000
1	12.209851000	21.749773000	19.908045000
1	11.527375000	18.804594000	20.451646000
1	12.322251000	18.391460000	18.107109000
1	12.487503000	20.139371000	17.853201000
1	10.890066000	19.433790000	18.115502000
1	13.933713000	18.350229000	20.013083000
1	13.738290000	19.433430000	21.401016000
1	14.243625000	20.087493000	19.831734000

6	9.533510000	16.174256000	19.376325000
6	9.338614000	16.381336000	20.880024000
8	10.304915000	16.444197000	21.646896000
6	10.323982000	14.885284000	19.086864000
6	9.601320000	13.573413000	19.453544000
6	8.314303000	13.356837000	18.640294000
6	10.555815000	12.381272000	19.282493000
1	10.110384000	17.028659000	19.002109000
1	11.265691000	14.943599000	19.646447000
1	10.584817000	14.860684000	18.018271000
1	9.327187000	13.629352000	20.517379000
1	7.851154000	12.396017000	18.899673000
1	7.567777000	14.139291000	18.813788000
1	8.535292000	13.334125000	17.563867000
1	10.074895000	11.440480000	19.578504000
1	11.461168000	12.503593000	19.889800000
1	10.868585000	12.277071000	18.234255000
7	8.045174000	16.464557000	21.282302000
6	7.646263000	16.507663000	22.679399000
6	7.139699000	17.884284000	23.155975000
8	6.126517000	18.408121000	22.306597000
6	8.254587000	18.920424000	23.256732000
1	7.297967000	16.391891000	20.583944000
1	8.506923000	16.205985000	23.283786000
1	6.721408000	17.724150000	24.167036000
1	5.572253000	17.669099000	21.933007000
1	7.837201000	19.896044000	23.529692000
1	8.774510000	19.019443000	22.299115000
1	8.991468000	18.627068000	24.012608000
6	3.042466000	14.045222000	20.895385000
6	1.706072000	14.789772000	20.836453000
8	0.658391000	14.217769000	20.543349000
6	4.080764000	14.505865000	19.853655000
6	4.912389000	15.752752000	20.219156000
8	4.636754000	16.350616000	21.305203000
8	5.843096000	16.063084000	19.421476000
1	3.478492000	14.153640000	21.895489000
1	4.806840000	13.701341000	19.681341000
1	3.605367000	14.675674000	18.879214000
7	1.778094000	16.124949000	21.128635000
6	0.651423000	17.026109000	20.901991000
6	-0.090828000	17.402688000	22.191721000
8	0.815883000	18.044073000	23.118345000
6	-0.786462000	16.220978000	22.862107000
1	2.720623000	16.497990000	21.252678000
1	-0.048140000	16.549162000	20.211807000
1	-0.823859000	18.173621000	21.944323000
1	1.595955000	17.467266000	23.186131000
1	-1.280440000	16.551749000	23.782664000
1	-0.069481000	15.431582000	23.114118000
1	-1.533018000	15.791307000	22.186936000
6	-2.146577000	23.394801000	22.398268000
6	-2.312058000	24.823311000	21.881426000
8	-1.617244000	25.749094000	22.328066000
6	-0.715143000	22.881942000	22.175514000
6	-0.589344000	21.381827000	22.411803000
8	-1.479150000	20.593014000	22.050455000
7	0.541820000	20.955132000	23.005258000
1	-2.370622000	23.395416000	23.472540000
1	-0.007221000	23.440579000	22.792656000
1	-0.417368000	23.062267000	21.133391000
1	1.279903000	21.607825000	23.230321000
1	0.727176000	19.948337000	23.075913000
7	-3.243645000	24.970227000	20.922705000
6	-3.606389000	26.241762000	20.304778000
6	-5.114982000	26.278272000	20.015174000
6	-5.593921000	25.154543000	19.063893000
8	-6.345996000	25.507303000	18.098336000
8	-5.231455000	23.974612000	19.319782000
1	-3.817223000	24.193932000	20.584310000
1	-3.050035000	26.387505000	19.368835000
1	-5.371777000	27.254620000	19.598016000
1	-5.663049000	26.162597000	20.961346000
6	-2.399996000	21.657627000	18.284758000
6	-3.404650000	20.603192000	18.698753000

8	-4.248684000	20.147082000	17.878251000
6	-0.921498000	21.245383000	18.485158000
6	-0.008197000	22.421860000	18.091818000
6	-0.593325000	19.954368000	17.720848000
6	1.469730000	22.220149000	18.438362000
1	-2.583879000	21.884144000	17.229961000
1	-0.765027000	21.046689000	19.553988000
1	-0.111993000	22.611807000	17.014710000
1	-0.371735000	23.329489000	18.595220000
1	0.447386000	19.657468000	17.887307000
1	-0.725960000	20.093674000	16.641068000
1	-1.222954000	19.119883000	18.050375000
1	2.036847000	23.139042000	18.266456000
1	1.600795000	21.953885000	19.495224000
1	1.928184000	21.428182000	17.836885000
7	-3.362958000	20.188079000	19.971264000
6	-4.156704000	19.079662000	20.517260000
6	-3.737371000	17.764416000	19.798604000
8	-2.566045000	17.400194000	19.836828000
6	-5.675942000	19.375522000	20.634157000
8	-6.375957000	19.236523000	19.390730000
6	-5.964373000	20.737074000	21.269089000
1	-2.619849000	20.526157000	20.590986000
1	-3.781380000	18.965449000	21.538866000
1	-6.089962000	18.587059000	21.276305000
1	-5.825481000	19.696834000	18.719773000
1	-7.042095000	20.832855000	21.434026000
1	-5.649030000	21.562634000	20.622126000
1	-5.453649000	20.838679000	22.234763000
7	-4.721489000	17.080114000	19.151437000
6	-4.431697000	15.936000000	18.312154000
6	-4.569505000	16.172631000	16.799685000
8	-4.629583000	15.219307000	16.031961000
1	-5.622703000	17.554005000	19.076884000
1	-3.399309000	15.634322000	18.514107000
7	-4.625400000	17.479106000	16.392937000
6	-4.568361000	17.838671000	14.986625000
6	-5.831744000	18.509426000	14.432468000
8	-5.770263000	19.298519000	13.489646000
1	-4.448680000	18.222063000	17.065359000
1	-3.737707000	18.523710000	14.798996000
1	-4.393662000	16.916900000	14.421510000
7	-7.002106000	18.121811000	15.005132000
6	-8.286192000	18.689890000	14.611393000
6	-8.625603000	19.953131000	15.424406000
8	-9.491412000	19.941745000	16.297034000
1	-6.947016000	17.595401000	15.866665000
1	-8.229049000	18.915438000	13.542788000
7	-7.853482000	21.021809000	15.111290000
6	-7.793152000	22.212357000	15.936968000
6	-7.751448000	23.461619000	15.036679000
8	-7.923351000	23.390706000	13.823586000
6	-6.603875000	22.126438000	16.900942000
8	-5.391271000	22.031814000	16.138818000
1	-7.145575000	20.920577000	14.391680000
1	-8.713012000	22.235744000	16.531868000
1	-6.550721000	22.972279000	17.593363000
1	-6.741116000	21.222747000	17.504589000
1	-4.815256000	21.409179000	16.627808000
7	-7.527214000	24.633653000	15.698016000
6	-7.345837000	25.866743000	14.965403000
6	-5.889626000	26.275785000	14.693534000
8	-5.653805000	27.376907000	14.188809000
1	-7.253206000	24.668095000	16.687984000
1	-7.833890000	25.766144000	13.992436000
7	-4.946383000	25.373682000	15.050668000
6	-3.521808000	25.520743000	14.773234000
6	-3.035675000	24.446391000	13.777566000
8	-2.334206000	24.767699000	12.817893000
6	-2.687812000	25.565095000	16.089289000
8	-2.841732000	26.796244000	16.764762000
6	-1.190315000	25.411696000	15.826951000
1	-5.258987000	24.567639000	15.579429000
1	-3.396688000	26.476741000	14.262784000
1	-3.030894000	24.726179000	16.722636000

1	-3.787021000	27.057048000	16.881985000
1	-0.651856000	25.548223000	16.769789000
1	-0.849658000	26.175751000	15.120047000
1	-0.942983000	24.427023000	15.420189000
7	-3.388385000	23.156506000	14.049015000
6	-3.183481000	22.062536000	13.098541000
6	-2.243480000	20.943115000	13.582062000
6	-0.736909000	21.167198000	13.541398000
6	0.106643000	20.093978000	13.882546000
6	-0.144877000	22.379443000	13.164877000
6	1.496029000	20.218547000	13.861853000
6	1.249293000	22.508488000	13.140447000
6	2.071446000	21.437381000	13.487564000
1	-4.073447000	22.972678000	14.778033000
1	-2.824700000	22.504770000	12.166426000
1	-2.538971000	20.672060000	14.607077000
1	-2.476174000	20.056478000	12.974665000
1	-0.337711000	19.140621000	14.166030000
1	-0.761130000	23.229786000	12.887289000
1	2.136516000	19.381904000	14.132766000
1	1.686550000	23.459835000	12.845019000
1	3.153756000	21.538852000	13.465824000
1	3.906367000	23.360577000	25.634610000
1	1.494156000	24.392319000	24.929831000
1	9.938701000	24.825553000	21.536692000
1	8.842510000	23.977478000	23.846232000
1	5.655328000	28.181230000	16.499784000
1	8.019955000	26.999523000	17.118991000
1	-0.243636000	27.287675000	20.917482000
1	0.896803000	28.393196000	18.698357000
1	8.573304000	16.170014000	18.850578000
1	6.842691000	15.775643000	22.819736000
1	2.799355000	12.990923000	20.737498000
1	1.032129000	17.942222000	20.436035000
1	-2.855991000	22.714458000	21.919018000
1	-3.320079000	27.044195000	20.990822000
1	-4.159636000	21.603025000	12.906415000
1	-2.621391000	22.575828000	18.845088000
1	9.927379000	22.106311000	20.999211000
1	-7.817908000	26.696013000	15.501638000
1	-9.073930000	17.955531000	14.790821000
8	-5.364224000	27.911351000	17.185528000
1	-5.583089000	28.056901000	16.248537000
1	-5.868038000	27.106222000	17.469456000
1	-5.080699000	15.089156000	18.558654000
6	6.945575000	19.308443000	18.909243000
8	4.082284000	24.280702000	20.177007000
6	9.182071000	23.133059000	17.141528000
1	9.793640000	23.826592000	16.558836000
1	9.005836000	22.221602000	16.562446000
1	8.209065000	23.600684000	17.330822000
1	6.680695000	27.830349000	21.766832000

IM2$^{4}_{C6}$

6	3.471715000	27.685011000	18.431109000
6	7.909553000	25.964292000	19.682920000
6	6.029754000	24.183124000	23.783992000
6	1.604926000	25.892584000	22.479046000
6	4.831095000	27.347144000	18.436701000
6	5.741693000	27.680388000	17.365551000
6	6.965134000	27.183312000	17.706722000
6	6.820080000	26.533994000	18.991520000
6	7.895570000	25.324673000	20.914877000
6	8.952757000	25.139036000	21.849104000
6	8.415682000	24.639586000	23.021252000
6	7.009911000	24.489093000	22.849481000
6	4.646365000	24.460460000	23.722620000
6	3.719343000	24.071785000	24.763253000
6	2.493108000	24.559768000	24.411994000
6	2.656811000	25.259976000	23.156908000
6	1.676949000	26.566544000	21.255628000
6	0.561559000	27.151339000	20.520643000
6	1.086110000	27.661111000	19.370828000
6	2.518177000	27.383290000	19.409175000
7	5.518038000	26.657156000	19.407908000
7	6.714674000	24.855193000	21.521395000
7	3.971903000	25.174739000	22.767946000
7	2.825579000	26.738711000	20.559179000
26	4.684130000	25.920277000	21.051142000
1	3.121681000	28.220124000	17.552236000
1	8.886981000	26.094455000	19.227272000
1	6.377073000	23.759322000	24.722780000
1	0.617194000	25.823752000	22.928017000
6	6.084021000	21.194205000	18.134594000
6	5.522104000	22.269502000	17.407886000
6	5.370695000	22.240652000	20.249045000
6	5.995448000	21.198929000	19.551094000
6	6.732936000	19.965349000	17.749601000
6	4.905363000	23.308284000	18.087005000
6	4.820288000	23.317999000	19.531757000
7	6.585270000	20.040848000	20.013159000
1	6.491177000	19.627433000	20.947355000
6	6.934106000	19.454359000	16.346553000
6	6.813299000	17.921173000	16.209079000
7	5.552631000	17.382543000	16.697410000
6	4.395279000	17.580499000	16.015598000
6	3.130615000	17.071600000	16.690190000
8	4.357155000	18.132647000	14.911616000
1	5.540521000	16.968534000	17.628987000
8	4.294007000	24.371779000	17.481111000
6	4.187537000	24.355408000	16.070748000
1	7.397670000	18.307942000	19.090394000
1	5.554252000	22.259715000	16.323842000
1	5.234484000	22.202439000	21.327161000
1	6.254722000	24.111230000	20.980822000
1	6.179585000	19.900529000	15.689181000
1	7.913409000	19.748463000	15.934783000
1	7.615419000	17.420357000	16.761618000
1	6.919863000	17.656927000	15.151425000
1	2.437096000	17.910318000	16.810242000
1	3.308795000	16.618872000	17.668915000
1	2.644641000	16.339206000	16.037146000
1	3.632242000	25.256468000	15.802600000
1	3.639763000	23.472086000	15.716029000
1	5.175641000	24.384173000	15.588238000
16	5.428761000	27.788064000	22.349027000
6	9.808853000	23.258636000	18.302898000
8	10.602946000	24.061360000	18.801116000
7	9.526915000	22.054524000	18.868144000
6	10.098123000	21.652319000	20.150116000
6	11.536266000	21.107752000	20.067071000
6	11.708898000	19.655603000	19.578025000
6	11.206713000	19.422125000	18.144137000
6	13.182705000	19.235406000	19.701085000
1	8.779836000	21.491936000	18.477134000
1	9.424694000	20.907110000	20.585579000
1	11.965396000	21.174487000	21.077649000
1	12.123955000	21.791052000	19.439957000
1	11.124334000	19.006671000	20.248795000
1	11.433264000	18.398413000	17.819040000
1	11.690885000	20.111497000	17.440028000
1	10.125154000	19.562979000	18.059348000
1	13.319997000	18.185151000	19.416594000
1	13.548858000	19.354657000	20.728158000
1	13.819236000	19.846129000	19.046180000
6	9.599198000	15.742394000	19.592174000
6	9.348620000	15.931040000	21.090763000
8	10.241826000	15.730983000	21.917301000
6	10.201048000	14.360264000	19.287942000
6	9.258580000	13.164555000	19.533767000
6	8.025411000	13.183215000	18.616102000
6	10.030625000	11.844984000	19.378029000
1	10.320718000	16.513325000	19.291394000
1	11.093803000	14.246242000	19.914702000
1	10.536574000	14.338694000	18.240263000
1	8.910342000	13.224289000	20.575516000
1	7.402100000	12.296364000	18.789073000
1	7.395773000	14.064759000	18.776593000
1	8.327412000	13.170181000	17.559363000

1	9.387439000	10.980524000	19.585497000
1	10.887810000	11.799933000	20.061194000
1	10.413828000	11.733550000	18.354125000
7	8.089974000	16.330308000	21.414890000
6	7.640838000	16.463846000	22.790466000
6	7.238621000	17.898359000	23.180638000
8	6.237359000	18.430858000	22.320054000
6	8.419328000	18.864776000	23.183012000
1	7.376881000	16.380581000	20.681802000
1	8.455746000	16.121426000	23.434340000
1	6.836976000	17.835449000	24.208746000
1	5.653050000	17.703663000	21.967927000
1	8.077498000	19.876801000	23.426999000
1	8.893652000	18.888323000	22.196257000
1	9.171890000	18.560889000	23.919292000
6	3.123098000	14.077416000	20.958537000
6	1.775361000	14.800074000	20.894325000
8	0.737319000	14.211266000	20.600437000
6	4.154036000	14.548856000	19.914296000
6	4.968185000	15.809289000	20.272996000
8	4.680428000	16.412335000	21.353465000
8	5.898467000	16.126104000	19.477620000
1	3.556912000	14.198355000	21.958133000
1	4.891190000	13.753831000	19.746115000
1	3.675487000	14.706241000	18.939280000
7	1.825775000	16.137239000	21.182854000
6	0.686098000	17.019850000	20.948061000
6	-0.073718000	17.377921000	22.232924000
8	0.819911000	18.015287000	23.175035000
6	-0.768139000	16.184938000	22.884353000
1	2.762650000	16.525776000	21.303776000
1	0.001112000	16.535028000	20.248723000
1	-0.809526000	18.146270000	21.986121000
1	1.602792000	17.441912000	23.241795000
1	-1.272881000	16.503414000	23.803446000
1	-0.049422000	15.396763000	23.134864000
1	-1.505862000	15.758064000	22.197756000
6	-2.206470000	23.323037000	22.479472000
6	-2.387941000	24.756673000	21.983708000
8	-1.705478000	25.683595000	22.445369000
6	-0.761926000	22.840980000	22.274775000
6	-0.614063000	21.339638000	22.488682000
8	-1.486748000	20.542658000	22.104625000
7	0.517525000	20.921076000	23.087283000
1	-2.450033000	23.300457000	23.549287000
1	-0.076174000	23.403376000	22.913828000
1	-0.448742000	23.043970000	21.241361000
1	1.242089000	21.581480000	23.331801000
1	0.715346000	19.915955000	23.149637000
7	-3.319572000	24.908151000	21.025187000
6	-3.684064000	26.185597000	20.420917000
6	-5.181799000	26.202302000	20.080422000
6	-5.605886000	25.091807000	19.088977000
8	-6.319882000	25.454893000	18.098704000
8	-5.239524000	23.911706000	19.337621000
1	-3.872379000	24.129141000	20.659963000
1	-3.098751000	26.361200000	19.507788000
1	-5.441459000	27.183035000	19.675487000
1	-5.759787000	26.056111000	21.004253000
6	-2.345727000	21.647724000	18.337666000
6	-3.351429000	20.581412000	18.717540000
8	-4.174662000	20.129753000	17.874109000
6	-0.869028000	21.239506000	18.558853000
6	0.047130000	22.425102000	18.201063000
6	-0.520116000	19.962049000	17.780789000
6	1.514410000	22.231271000	18.594479000
1	-2.512029000	21.889432000	17.283329000
1	-0.731910000	21.024899000	19.627267000
1	-0.023311000	22.620219000	17.122170000
1	-0.337745000	23.327756000	18.697295000
1	0.519765000	19.669071000	17.959059000
1	-0.636608000	20.116453000	16.701113000
1	-1.149400000	19.118788000	18.087953000
1	2.082111000	23.152830000	18.439436000
1	1.611295000	21.964892000	19.655129000

1	1.996654000	21.441451000	18.008990000
7	-3.334034000	20.151152000	19.985690000
6	-4.131613000	19.030278000	20.500158000
6	-3.688276000	17.727206000	19.773685000
8	-2.515380000	17.370567000	19.831640000
6	-5.654996000	19.314396000	20.589255000
8	-6.328501000	19.186352000	19.330178000
6	-5.966831000	20.665649000	21.234979000
1	-2.605551000	20.484843000	20.624777000
1	-3.777043000	18.904817000	21.527844000
1	-6.076339000	18.514945000	21.212774000
1	-5.768852000	19.660527000	18.676891000
1	-7.047994000	20.749193000	21.383335000
1	-5.649588000	21.501657000	20.602528000
1	-5.473072000	20.760967000	22.210100000
7	-4.654001000	17.044751000	19.097568000
6	-4.339465000	15.911436000	18.252712000
6	-4.435275000	16.164644000	16.739682000
8	-4.467044000	15.219561000	15.960189000
1	-5.556832000	17.513452000	19.010177000
1	-3.312491000	15.608649000	18.478921000
7	-4.486342000	17.475167000	16.345554000
6	-4.383912000	17.850347000	14.945784000
6	-5.632007000	18.518340000	14.354803000
8	-5.543421000	19.317915000	13.423072000
1	-4.335413000	18.211577000	17.031241000
1	-3.552292000	18.543113000	14.794762000
1	-4.182757000	16.936087000	14.377286000
7	-6.818736000	18.116603000	14.882300000
6	-8.091987000	18.681875000	14.451282000
6	-8.467899000	19.929251000	15.272472000
8	-9.353934000	19.894301000	16.123965000
1	-6.789918000	17.581303000	15.739641000
1	-7.997888000	18.924535000	13.389104000
7	-7.702777000	21.012470000	14.993622000
6	-7.677079000	22.189412000	15.840600000
6	-7.609885000	23.453752000	14.963307000
8	-7.733524000	23.402104000	13.743386000
6	-6.521311000	22.092943000	16.843442000
8	-5.283733000	22.018918000	16.120803000
1	-6.973086000	20.929899000	14.293818000
1	-8.616967000	22.199027000	16.403662000
1	-6.497266000	22.926908000	17.551672000
1	-6.673689000	21.177947000	17.426349000
1	-4.716967000	21.397605000	16.622205000
7	-7.423156000	24.615951000	15.652994000
6	-7.228134000	25.864175000	14.950088000
6	-5.768079000	26.292316000	14.733770000
8	-5.528069000	27.401106000	14.248314000
1	-7.183825000	24.634311000	16.652195000
1	-7.681951000	25.777957000	13.959343000
7	-4.826353000	25.398063000	15.114515000
6	-3.396512000	25.559307000	14.874207000
6	-2.879713000	24.501548000	13.875406000
8	-2.150592000	24.839354000	12.942377000
6	-2.593132000	25.593496000	16.209660000
8	-2.772344000	26.814581000	16.897661000
6	-1.088867000	25.455472000	15.979464000
1	-5.146107000	24.585459000	15.628595000
1	-3.266790000	26.522805000	14.379126000
1	-2.943523000	24.743790000	16.824253000
1	-3.722881000	27.063533000	16.998850000
1	-0.572487000	25.588451000	16.935237000
1	-0.739640000	26.228103000	15.286167000
1	-0.824161000	24.476610000	15.569662000
7	-3.241424000	23.207797000	14.113608000
6	-3.020357000	22.131158000	13.146925000
6	-2.095783000	20.998822000	13.629629000
6	-0.587286000	21.210429000	13.605059000
6	0.244043000	20.122665000	13.929733000
6	0.018146000	22.424730000	13.257503000
6	1.634575000	20.233726000	13.918833000
6	1.413625000	22.540284000	13.242100000
6	2.223485000	21.454065000	13.571228000
1	-3.945488000	23.011731000	14.820914000

1	-2.640490000	22.588615000	12.230706000
1	-2.404170000	20.721880000	14.649224000
1	-2.330227000	20.119584000	13.012585000
1	-0.211065000	19.168008000	14.190921000
1	-0.588439000	23.286731000	12.994642000
1	2.265013000	19.385013000	14.175501000
1	1.861704000	23.492672000	12.966717000
1	3.306912000	21.544728000	13.554395000
1	3.985807000	23.499677000	25.643589000
1	1.559267000	24.482463000	24.955375000
1	9.979669000	25.428242000	21.669588000
1	8.939820000	24.476089000	23.954580000
1	5.466350000	28.226055000	16.471463000
1	7.891961000	27.238504000	17.149442000
1	-0.466856000	27.129506000	20.856938000
1	0.558553000	28.151298000	18.561818000
1	8.684068000	15.912997000	19.015847000
1	6.775157000	15.807597000	22.941470000
1	2.897435000	13.018421000	20.806342000
1	1.056586000	17.944067000	20.490054000
1	-2.894539000	22.638575000	21.975789000
1	-3.435903000	26.979010000	21.132033000
1	-3.994251000	21.680057000	12.926155000
1	-2.582467000	22.555811000	18.908111000
1	10.093852000	22.529084000	20.805991000
1	-7.726373000	26.678666000	15.485335000
1	-8.880322000	17.939513000	14.590914000
8	-5.321612000	27.884803000	17.267965000
1	-5.512329000	28.047478000	16.327615000
1	-5.827165000	27.069772000	17.518526000
1	-4.993643000	15.061036000	18.471702000
6	7.001204000	19.298859000	18.929588000
8	4.181456000	24.299986000	20.152416000
6	9.060749000	23.564570000	17.013116000
1	9.700240000	24.181211000	16.376352000
1	8.761182000	22.662637000	16.471208000
1	8.153144000	24.132710000	17.245957000
1	5.790642000	28.580349000	21.315170000

P^{2}_{C6}

6	3.664982000	27.862768000	18.560745000
6	7.929626000	26.119936000	20.056069000
6	5.796317000	24.092687000	23.901624000
6	1.565369000	26.010303000	22.507448000
6	5.016849000	27.555242000	18.633276000
6	5.996619000	27.946186000	17.649737000
6	7.195915000	27.462663000	18.077070000
6	6.949517000	26.775335000	19.322267000
6	7.709075000	25.420546000	21.234709000
6	8.744470000	24.718981000	21.956038000
6	8.146008000	24.145356000	23.035538000
6	6.746863000	24.495288000	22.973309000
6	4.448749000	24.430622000	23.855787000
6	3.473227000	24.061300000	24.846383000
6	2.287228000	24.621179000	24.461178000
6	2.535106000	25.324798000	23.229190000
6	1.785514000	26.671343000	21.307216000
6	0.728647000	27.294320000	20.541848000
6	1.311242000	27.808312000	19.425591000
6	2.720607000	27.500144000	19.510217000
7	5.615665000	26.842924000	19.643418000
7	6.498400000	25.272889000	21.865642000
7	3.857661000	25.185165000	22.863663000
7	2.991819000	26.803193000	20.662278000
26	4.768003000	26.137820000	21.308774000
1	3.320653000	28.425093000	17.698036000
1	8.941558000	26.112208000	19.662995000
1	6.133353000	23.481404000	24.733413000
1	0.543498000	26.000783000	22.875969000
6	6.213832000	21.153324000	18.164408000
6	5.601322000	22.222450000	17.476369000
6	5.508430000	22.117940000	20.318420000
6	6.164027000	21.122727000	19.582713000
6	6.909354000	19.961720000	17.738136000

6	4.940895000	23.210276000	18.197144000
6	4.890285000	23.140146000	19.618480000
7	6.825587000	19.991597000	20.007743000
1	6.750170000	19.552942000	20.933785000
6	7.079359000	19.474324000	16.322571000
6	6.904713000	17.947460000	16.164596000
7	5.636261000	17.443422000	16.668761000
6	4.477915000	17.665500000	15.996003000
6	3.210577000	17.179624000	16.682325000
8	4.441492000	18.217794000	14.892014000
1	5.616327000	17.040094000	17.605294000
8	4.276260000	24.272222000	17.646636000
6	4.197285000	24.341060000	16.234622000
1	7.698723000	18.320913000	19.027123000
1	5.619165000	22.252350000	16.392883000
1	5.469109000	22.091504000	21.404339000
1	6.330497000	19.955268000	15.683717000
1	8.061968000	19.744030000	15.903522000
1	7.701192000	17.413400000	16.693786000
1	6.981264000	17.694964000	15.101647000
1	2.531622000	18.029430000	16.807486000
1	3.392717000	16.724456000	17.659012000
1	2.707329000	16.454401000	16.034308000
1	3.615234000	25.237602000	16.014013000
1	3.688051000	23.463526000	15.814087000
1	5.193771000	24.434103000	15.779781000
16	5.192867000	27.983505000	22.509205000
6	9.898854000	23.280056000	18.587116000
8	10.657962000	24.057448000	19.173303000
7	9.699117000	21.998357000	18.995585000
6	10.297170000	21.479932000	20.222689000
6	11.754647000	21.003694000	20.077150000
6	11.970511000	19.604488000	19.466668000
6	11.461033000	19.475352000	18.022312000
6	13.457763000	19.222971000	19.542220000
1	8.967077000	21.453985000	18.554148000
1	9.657617000	20.665230000	20.576015000
1	12.196192000	21.001139000	21.084351000
1	12.308167000	21.758428000	19.503012000
1	11.413408000	18.883432000	20.085266000
1	11.694105000	18.481110000	17.619900000
1	11.934430000	20.220719000	17.369917000
1	10.377995000	19.613821000	17.953761000
1	13.626232000	18.207545000	19.163363000
1	13.829134000	19.261346000	20.573546000
1	14.068671000	19.910360000	18.941202000
6	9.570449000	15.703741000	19.362026000
6	9.387825000	15.860519000	20.874044000
8	10.311593000	15.625125000	21.656246000
6	10.112983000	14.311204000	18.998072000
6	9.152311000	13.137029000	19.274624000
6	7.863116000	13.215212000	18.440594000
6	9.872734000	11.801077000	19.034873000
1	10.304521000	16.457440000	19.047748000
1	11.036563000	14.158719000	19.569752000
1	10.387366000	14.302776000	17.932634000
1	8.874403000	13.178206000	20.338181000
1	7.228373000	12.339604000	18.628754000
1	7.267928000	14.106550000	18.666112000
1	8.095116000	13.226371000	17.366292000
1	9.219956000	10.949905000	19.265575000
1	10.772625000	11.715164000	19.656355000
1	10.181545000	11.705625000	17.984646000
7	8.150221000	16.269747000	21.261511000
6	7.758439000	16.363507000	22.658052000
6	7.439855000	17.795876000	23.125918000
8	6.439105000	18.415897000	22.324124000
6	8.666286000	18.702973000	23.130097000
1	7.407278000	16.355304000	20.561108000
1	8.578103000	15.955538000	23.256328000
1	7.067798000	17.705262000	24.162823000
1	5.794327000	17.737098000	21.978797000
1	8.382463000	19.719867000	23.422739000
1	9.113402000	18.742336000	22.131346000
1	9.423889000	18.333226000	23.829955000

6	3.093622000	14.229940000	21.058841000	7	-4.660765000	17.117968000	19.248009000
6	1.757781000	14.975042000	21.034628000	6	-4.332473000	15.959266000	18.443609000
8	0.702662000	14.405023000	20.766044000	6	-4.418648000	16.161484000	16.922154000
6	4.087790000	14.672078000	19.966685000	8	-4.434951000	15.190814000	16.174281000
6	4.949972000	15.910822000	20.286788000	1	-5.567069000	17.575981000	19.141396000
8	4.718819000	16.527136000	21.373227000	1	-3.305367000	15.670679000	18.687212000
8	5.860561000	16.195450000	19.457038000	7	-4.479055000	17.457442000	16.484108000
1	3.569714000	14.357173000	22.038034000	6	-4.371416000	17.787377000	15.073424000
1	4.795401000	13.857853000	19.766983000	6	-5.625931000	18.413984000	14.451207000
1	3.569151000	14.840642000	19.014241000	8	-5.543520000	19.188180000	13.497767000
7	1.836773000	16.311669000	21.323064000	1	-4.344255000	18.217767000	17.146506000
6	0.707075000	17.208941000	21.091331000	1	-3.550411000	18.488890000	14.905904000
6	-0.046456000	17.585490000	22.374082000	1	-4.150302000	16.858209000	14.537441000
8	0.844430000	18.255237000	23.297153000	7	-6.809818000	18.004797000	14.979322000
6	-0.723753000	16.399972000	23.055950000	6	-8.089222000	18.535044000	14.523083000
1	2.783075000	16.685803000	21.409827000	6	-8.490551000	19.799134000	15.305880000
1	0.011819000	16.728832000	20.398482000	8	-9.374540000	19.771996000	16.159758000
1	-0.792099000	18.339866000	22.113821000	1	-6.779211000	17.494285000	15.851532000
1	1.631853000	17.690384000	23.378565000	1	-7.992063000	18.747809000	13.454827000
1	-1.233616000	16.734595000	23.966433000	7	-7.747448000	20.888624000	14.993771000
1	0.007125000	15.630018000	23.327255000	6	-7.745077000	22.090764000	15.804995000
1	-1.453836000	15.944064000	22.380202000	6	-7.698903000	23.328599000	14.889540000
6	-2.258343000	23.504347000	22.402633000	8	-7.829779000	23.238160000	13.672686000
6	-2.441869000	24.916889000	21.847990000	6	-6.591088000	22.045412000	16.813404000
8	-1.765582000	25.863460000	22.277264000	8	-5.349504000	21.974647000	16.097359000
6	-0.807784000	23.026342000	22.233812000	1	-7.015751000	20.799077000	14.297043000
6	-0.646237000	21.536623000	22.505724000	1	-8.686583000	22.100218000	16.365441000
8	-1.485241000	20.712386000	22.105499000	1	-6.584964000	22.899457000	17.497730000
7	0.464376000	21.156366000	23.166956000	1	-6.728285000	21.144363000	17.421178000
1	-2.514254000	23.522974000	23.469653000	1	-4.768468000	21.391165000	16.626923000
1	-0.134318000	23.621718000	22.854961000	7	-7.521570000	24.513743000	15.541908000
1	-0.488530000	23.189529000	21.195024000	6	-7.337690000	25.739611000	14.797782000
1	1.148149000	21.840259000	23.460660000	6	-5.880664000	26.167035000	14.561459000
1	0.677161000	20.157671000	23.268585000	8	-5.647487000	27.263608000	14.045552000
7	-3.367572000	25.024698000	20.878131000	1	-7.272230000	24.564711000	16.537343000
6	-3.740635000	26.274999000	20.223985000	1	-7.796919000	25.619468000	13.813120000
6	-5.240439000	26.270288000	19.891512000	7	-4.934004000	25.287074000	14.962020000
6	-5.665933000	25.121023000	18.945115000	6	-3.503994000	25.458669000	14.730906000
8	-6.403791000	25.440597000	17.957241000	6	-2.963575000	24.383411000	13.765033000
8	-5.277472000	23.954919000	19.225406000	8	-2.232176000	24.707770000	12.829021000
1	-3.914226000	24.228739000	20.541328000	6	-2.715916000	25.534784000	16.074213000
1	-3.161139000	26.415422000	19.301395000	8	-2.899608000	26.778768000	16.718072000
1	-5.508531000	27.233669000	19.451925000	6	-1.209177000	25.385154000	15.868557000
1	-5.812712000	26.156141000	20.823497000	1	-5.247237000	24.486746000	15.499354000
6	-2.365669000	21.685394000	18.333519000	1	-3.379281000	26.411014000	14.213591000
6	-3.384120000	20.644861000	18.749794000	1	-3.077069000	24.707151000	16.712311000
8	-4.209667000	20.170119000	17.922199000	1	-3.850541000	27.033049000	16.802530000
6	-0.895753000	21.248557000	18.547099000	1	-0.705323000	25.550993000	16.825840000
6	0.044343000	22.406277000	18.162203000	1	-0.847740000	26.131786000	15.153533000
6	-0.582505000	19.951741000	17.785957000	1	-0.942124000	24.391424000	15.497857000
6	1.507417000	22.184601000	18.556760000	7	-3.304063000	23.090334000	14.036487000
1	-2.540495000	21.907717000	17.276276000	6	-3.046279000	21.990501000	13.105537000
1	-0.753850000	21.048741000	19.617893000	6	-2.096317000	20.897652000	13.629588000
1	-0.022986000	22.581971000	17.079877000	6	-0.592650000	21.141997000	13.599582000
1	-0.317770000	23.327421000	18.641222000	6	0.262867000	20.076892000	13.936430000
1	0.451458000	19.636460000	17.960435000	6	-0.014274000	22.365099000	13.236824000
1	-0.704833000	20.092431000	16.704995000	6	1.650647000	20.218876000	13.922547000
1	-1.227552000	19.127683000	18.111180000	6	1.378223000	22.512235000	13.220790000
1	2.098591000	23.086792000	18.377243000	6	2.212202000	21.448349000	13.562043000
1	1.596607000	21.938058000	19.623543000	1	-4.012533000	22.902945000	14.741746000
1	1.967539000	21.368431000	17.989986000	1	-2.669879000	22.429934000	12.179010000
7	-3.367826000	20.258987000	20.032467000	1	-2.400867000	20.649847000	14.657850000
6	-4.162095000	19.153883000	20.585424000	1	-2.308732000	19.991857000	13.043934000
6	-3.705357000	17.828561000	19.909398000	1	-0.170673000	19.115098000	14.207946000
8	-2.530470000	17.482872000	19.989059000	1	-0.639888000	23.208602000	12.959269000
6	-5.687657000	19.430335000	20.657231000	1	2.301089000	19.386996000	14.184060000
8	-6.354434000	19.250480000	19.401333000	1	1.804635000	23.471082000	12.933741000
6	-6.011867000	20.802350000	21.251076000	1	3.293428000	21.562240000	13.542744000
1	-2.631425000	20.607194000	20.654571000	1	3.683831000	23.466073000	25.726457000
1	-3.811711000	19.069109000	21.618675000	1	1.329502000	24.588098000	24.966240000
1	-6.105711000	18.651280000	21.308163000	1	9.774431000	24.662974000	21.629033000
1	-5.798358000	19.709988000	18.734567000	1	8.590801000	23.528604000	23.806791000
1	-7.094136000	20.882966000	21.392917000	1	5.775660000	28.511360000	16.752705000
1	-5.698762000	21.616603000	20.588850000	1	8.167252000	27.546949000	17.605964000
1	-5.522406000	20.937968000	22.223684000	1	-0.311825000	27.284068000	20.840965000

1	0.842139000	28.331762000	18.601909000
1	8.637404000	15.916484000	18.829758000
1	6.868419000	15.741884000	22.812413000
1	2.844536000	13.173168000	20.929695000
1	1.086716000	18.126143000	20.626589000
1	-2.936270000	22.796191000	21.918051000
1	-3.491967000	27.097436000	20.900997000
1	-4.006048000	21.507357000	12.891279000
1	-2.575441000	22.609710000	18.887735000
1	10.260117000	22.276531000	20.971668000
1	-7.836529000	26.568418000	15.309906000
1	-8.865792000	17.783739000	14.679702000
8	-5.451939000	27.853793000	17.033866000
1	-5.642557000	27.975985000	16.087251000
1	-5.945270000	27.041663000	17.316437000
1	-4.982921000	15.112353000	18.685904000
6	7.241264000	19.290075000	18.896921000
6	9.111113000	23.705676000	17.357304000
1	9.731010000	24.381455000	16.762087000
1	8.790474000	22.860881000	16.740878000
1	8.216454000	24.252745000	17.674378000
1	4.714993000	28.885893000	21.621721000
8	4.149906000	24.118196000	20.265418000
1	3.911942000	23.803000000	21.155938000

P^4_{C6}

6	3.655860000	27.955857000	18.513851000
6	7.957437000	26.221555000	20.003029000
6	5.848942000	24.379048000	23.955970000
6	1.531245000	26.063960000	22.426720000
6	5.031286000	27.718778000	18.611300000
6	6.010769000	28.099519000	17.616692000
6	7.214724000	27.609403000	18.030054000
6	6.987247000	26.926805000	19.285321000
6	7.767623000	25.508925000	21.191250000
6	8.806150000	24.820040000	21.928939000
6	8.216536000	24.311147000	23.049649000
6	6.817034000	24.680374000	22.993769000
6	4.493012000	24.720894000	23.920053000
6	3.518265000	24.380240000	24.928104000
6	2.310542000	24.855720000	24.497504000
6	2.531329000	25.490606000	23.219298000
6	1.705700000	26.683044000	21.186874000
6	0.642151000	27.236063000	20.370863000
6	1.238149000	27.771420000	19.267612000
6	2.664775000	27.545675000	19.408242000
7	5.657054000	27.025549000	19.616602000
7	6.576036000	25.393623000	21.851297000
7	3.866416000	25.388447000	22.888669000
7	2.911548000	26.876709000	20.573875000
26	4.789322000	26.381870000	21.330473000
1	3.322523000	28.497832000	17.632843000
1	8.959799000	26.195464000	19.584747000
1	6.182286000	23.819902000	24.826218000
1	0.508728000	26.008044000	22.791744000
6	6.192880000	21.102104000	18.184457000
6	5.578893000	22.170699000	17.495715000
6	5.501189000	22.078056000	20.338675000
6	6.149525000	21.077063000	19.602500000
6	6.884713000	19.908007000	17.758465000
6	4.929795000	23.164157000	18.216668000
6	4.883771000	23.103622000	19.640935000
7	6.812085000	19.946129000	20.028160000
1	6.733033000	19.506028000	20.952754000
6	7.053592000	19.421952000	16.342417000
6	6.881212000	17.895413000	16.182023000
7	5.611366000	17.390375000	16.681795000
6	4.455029000	17.614047000	16.006618000
6	3.185042000	17.130324000	16.689719000
8	4.421756000	18.167186000	14.902825000
1	5.589787000	16.986238000	17.617886000
8	4.273736000	24.233421000	17.666956000
6	4.185872000	24.296797000	16.255743000
1	7.677837000	18.269741000	19.048387000
1	5.592141000	22.197554000	16.412017000
1	5.464938000	22.051874000	21.425021000
1	6.303481000	19.902516000	15.704683000
1	8.035435000	19.693987000	15.922874000
1	7.676290000	17.361355000	16.713296000
1	6.961081000	17.643932000	15.119048000
1	2.507184000	17.981348000	16.812568000
1	3.363480000	16.675402000	17.667243000
1	2.682368000	16.405535000	16.040738000
1	3.610989000	25.197837000	16.034019000
1	3.665796000	23.422379000	15.841902000
1	5.180152000	24.378002000	15.793186000
16	5.187837000	28.331622000	22.559502000
6	9.838792000	23.298544000	18.574690000
8	10.605260000	24.080548000	19.145805000
7	9.641296000	22.021130000	18.995708000
6	10.249840000	21.510099000	20.220921000
6	11.706369000	21.034001000	20.065687000
6	11.917792000	19.631470000	19.461265000
6	11.392371000	19.492522000	18.023576000
6	13.406442000	19.252930000	19.523108000
1	8.904971000	21.474101000	18.564953000
1	9.613465000	20.696811000	20.583185000
1	12.156755000	21.037596000	21.069003000
1	12.254399000	21.785696000	19.482357000
1	11.368308000	18.913393000	20.090089000
1	11.627001000	18.497754000	17.623308000
1	11.853638000	20.237502000	17.362112000
1	10.307663000	19.623551000	17.967498000
1	13.572460000	18.235211000	19.149391000
1	13.789329000	19.299090000	20.549935000
1	14.009470000	19.937122000	18.910577000
6	9.557690000	15.658543000	19.356736000
6	9.375504000	15.801907000	20.870161000
8	10.298431000	15.556796000	21.650410000
6	10.106438000	14.272173000	18.979586000
6	9.151236000	13.091141000	19.245794000
6	7.859683000	13.172520000	18.415684000
6	9.876656000	11.760853000	18.990146000
1	10.287197000	16.419218000	19.048375000
1	11.031009000	14.118274000	19.549270000
1	10.380123000	14.274943000	17.913925000
1	8.875883000	13.119961000	20.310419000
1	7.229349000	12.292066000	18.595971000
1	7.260931000	14.058678000	18.651926000
1	8.089040000	13.196115000	17.341001000
1	9.228243000	10.904503000	19.213954000
1	10.778821000	11.672394000	19.607974000
1	10.182594000	11.677650000	17.938036000
7	8.139541000	16.213087000	21.260704000
6	7.748839000	16.299615000	22.657900000
6	7.429093000	17.729501000	23.132271000
8	6.424300000	18.350774000	22.336668000
6	8.653650000	18.639284000	23.135428000
1	7.395502000	16.301698000	20.561672000
1	8.569439000	15.889722000	23.253577000
1	7.061290000	17.634104000	24.170349000
1	5.780553000	17.672412000	21.989597000
1	8.369280000	19.653387000	23.437096000
1	9.094492000	18.686869000	22.134193000
1	9.416227000	18.266439000	23.828245000
6	3.073070000	14.168878000	21.050651000
6	1.739648000	14.917693000	21.016087000
8	0.685047000	14.350988000	20.738490000
6	4.077213000	14.608370000	19.966777000
6	4.936709000	15.847946000	20.291285000
8	4.703031000	16.461417000	21.378709000
8	5.848004000	16.135218000	19.463332000
1	3.541826000	14.294774000	22.033556000
1	4.786935000	13.793620000	19.776412000
1	3.568079000	14.774049000	19.008784000
7	1.819960000	16.253900000	21.306485000
6	0.693993000	17.154062000	21.068297000
6	-0.068987000	17.526992000	22.346571000
8	0.816740000	18.189623000	23.279627000

6	-0.754941000	16.340468000	23.017927000
1	2.766553000	16.625570000	21.401070000
1	0.003468000	16.677799000	20.368148000
1	-0.810816000	18.284522000	22.084489000
1	1.602735000	17.623030000	23.362901000
1	-1.270607000	16.672685000	23.926023000
1	-0.028349000	15.567225000	23.291247000
1	-1.481232000	15.889749000	22.334702000
6	-2.268315000	23.456985000	22.418123000
6	-2.447344000	24.869216000	21.862236000
8	-1.754713000	25.811217000	22.275829000
6	-0.817210000	22.975597000	22.265967000
6	-0.667554000	21.481720000	22.525461000
8	-1.517010000	20.667715000	22.127352000
7	0.444679000	21.087069000	23.175940000
1	-2.536502000	23.474854000	23.482279000
1	-0.150146000	23.561261000	22.904248000
1	-0.480613000	23.148100000	21.234260000
1	1.142012000	21.762634000	23.454454000
1	0.653842000	20.086063000	23.264268000
7	-3.391141000	24.985912000	20.911386000
6	-3.764372000	26.242043000	20.268001000
6	-5.264279000	26.239324000	19.936376000
6	-5.686775000	25.096778000	18.980889000
8	-6.409189000	25.424834000	17.984575000
8	-5.309627000	23.927346000	19.262585000
1	-3.945838000	24.194127000	20.577429000
1	-3.185151000	26.391202000	19.346519000
1	-5.532592000	27.206005000	19.504351000
1	-5.835845000	26.117100000	20.867687000
6	-2.372539000	21.668455000	18.350888000
6	-3.388287000	20.618673000	18.750026000
8	-4.206022000	20.147768000	17.912577000
6	-0.901802000	21.238339000	18.572513000
6	0.035397000	22.404969000	18.207821000
6	-0.574384000	19.950703000	17.801804000
6	1.494529000	22.189838000	18.620277000
1	-2.540450000	21.899185000	17.294339000
1	-0.767931000	21.028667000	19.642450000
1	-0.019340000	22.587757000	17.125948000
1	-0.339070000	23.320461000	18.688270000
1	0.460332000	19.640736000	17.981104000
1	-0.689316000	20.100659000	16.721315000
1	-1.216640000	19.119563000	18.114465000
1	2.082723000	23.096895000	18.456468000
1	1.571575000	21.935729000	19.686112000
1	1.967452000	21.381062000	18.053483000
7	-3.379391000	20.220844000	20.029175000
6	-4.173725000	19.107881000	20.566067000
6	-3.711027000	17.790746000	19.878094000
8	-2.535726000	17.446756000	19.959470000
6	-5.700206000	19.380464000	20.633840000
8	-6.361020000	19.212344000	19.373126000
6	-6.030237000	20.745373000	21.240703000
1	-2.650282000	20.567663000	20.660110000
1	-3.828077000	19.012531000	21.599994000
1	-6.119609000	18.593858000	21.274761000
1	-5.802022000	19.677841000	18.713060000
1	-7.113221000	20.821638000	21.379426000
1	-5.717126000	21.567456000	20.588257000
1	-5.544667000	20.871977000	22.216482000
7	-4.662113000	17.084059000	19.206624000
6	-4.328461000	15.932279000	18.394561000
6	-4.406774000	16.147017000	16.874406000
8	-4.421562000	15.182554000	16.118541000
1	-5.568667000	17.541371000	19.099407000
1	-3.302391000	15.642549000	18.641087000
7	-4.464199000	17.446630000	16.446905000
6	-4.349403000	17.788231000	15.039560000
6	-5.599520000	18.423288000	14.417163000
8	-5.510818000	19.205247000	13.470613000
1	-4.331520000	18.201210000	17.116229000
1	-3.525903000	18.489054000	14.881644000
1	-4.128104000	16.863066000	14.496769000
7	-6.786838000	18.012289000	14.935989000
6	-8.062914000	18.548338000	14.477169000
6	-8.467661000	19.806218000	15.268218000
8	-9.359131000	19.773640000	16.114075000
1	-6.761702000	17.493851000	15.803685000
1	-7.959253000	18.770408000	13.411407000
7	-7.719603000	20.896754000	14.971956000
6	-7.722930000	22.092556000	15.792419000
6	-7.668187000	23.337407000	14.886926000
8	-7.786998000	23.256274000	13.668183000
6	-6.576888000	22.038924000	16.809508000
8	-5.329643000	21.974626000	16.102484000
1	-6.982855000	20.812135000	14.279917000
1	-8.668876000	22.098417000	16.345355000
1	-6.576579000	22.887141000	17.501052000
1	-6.718322000	21.132897000	17.408854000
1	-4.754710000	21.380724000	16.627095000
7	-7.496754000	24.517433000	15.549996000
6	-7.308008000	25.749506000	14.817479000
6	-5.849786000	26.181827000	14.597886000
8	-5.614336000	27.282509000	14.091972000
1	-7.259120000	24.561092000	16.548641000
1	-7.757472000	25.636120000	13.827501000
7	-4.904494000	25.301031000	15.000298000
6	-3.473438000	25.474582000	14.776602000
6	-2.927910000	24.404451000	13.807549000
8	-2.190940000	24.734020000	12.877782000
6	-2.690648000	25.545435000	16.123192000
8	-2.883083000	26.784149000	16.774995000
6	-1.182385000	25.405085000	15.922020000
1	-5.220847000	24.495645000	15.528040000
1	-3.347539000	26.429625000	14.264531000
1	-3.049800000	24.711681000	16.754417000
1	-3.835780000	27.032188000	16.858626000
1	-0.682281000	25.570013000	16.881517000
1	-0.823018000	26.156401000	15.210843000
1	-0.908519000	24.414427000	15.548128000
7	-3.271729000	23.110147000	14.068710000
6	-3.014045000	22.016302000	13.130562000
6	-2.071235000	20.915637000	13.651055000
6	-0.566391000	21.152762000	13.626853000
6	0.282886000	20.080927000	13.958128000
6	0.018940000	22.375646000	13.274721000
6	1.671411000	20.215777000	13.948840000
6	1.412216000	22.515594000	13.262844000
6	2.239922000	21.444930000	13.598298000
1	-3.983837000	22.919026000	14.769273000
1	-2.631608000	22.460996000	12.209061000
1	-2.380252000	20.663486000	14.676935000
1	-2.286314000	20.014331000	13.059500000
1	-0.156225000	19.119427000	14.221616000
1	-0.601719000	23.224400000	13.002031000
1	2.316650000	19.378486000	14.206117000
1	1.844339000	23.474199000	12.983599000
1	3.321731000	21.553601000	13.582167000
1	3.738067000	23.845271000	25.844042000
1	1.350958000	24.797890000	24.996804000
1	9.832518000	24.732782000	21.597568000
1	8.675196000	23.732405000	23.842218000
1	5.787327000	28.649944000	16.710991000
1	8.174238000	27.678847000	17.532842000
1	-0.407312000	27.190406000	20.632180000
1	0.762445000	28.264544000	18.428954000
1	8.623132000	15.871710000	18.827347000
1	6.859589000	15.676346000	22.810509000
1	2.822077000	13.112830000	20.919219000
1	1.079228000	18.072135000	20.609979000
1	-2.941634000	22.749284000	21.926773000
1	-3.515820000	27.058962000	20.951829000
1	-3.974929000	21.538984000	12.908304000
1	-2.592099000	22.586583000	18.911687000
1	10.218334000	22.310688000	20.965984000
1	-7.813570000	26.573352000	15.331033000
1	-8.841357000	17.796735000	14.622641000
8	-5.441376000	27.843796000	17.090141000
1	-5.625344000	27.976793000	16.143678000

1	-5.937901000	27.029391000	17.360239000
1	-4.979464000	15.082916000	18.626569000
6	7.221476000	19.239165000	18.916963000
6	9.039324000	23.716028000	17.349704000
1	9.663348000	24.365613000	16.729899000
1	8.687938000	22.867039000	16.756619000
1	8.163487000	24.290199000	17.671352000
1	5.816143000	29.028906000	21.585160000
8	4.167775000	24.095692000	20.271694000
1	4.067325000	23.880144000	21.215500000

TS1$^{2}_{HA}$

6	2.802203000	25.522751000	17.455373000
6	7.354717000	26.992382000	16.720586000
6	8.307123000	26.225877000	21.405121000
6	3.734220000	24.846366000	22.143728000
6	3.975485000	25.960314000	16.842667000
6	4.073784000	26.303013000	15.443256000
6	5.352759000	26.718692000	15.231400000
6	6.031483000	26.627458000	16.501920000
6	8.020838000	26.923082000	17.941427000
6	9.394716000	27.302492000	18.155551000
6	9.661671000	27.071757000	19.472999000
6	8.449640000	26.557743000	20.060328000
6	7.141180000	25.800932000	22.026091000
6	7.027357000	25.477985000	23.429912000
6	5.738823000	25.094046000	23.638975000
6	5.067284000	25.180286000	22.362351000
6	3.075247000	24.906534000	20.921768000
6	1.684194000	24.553222000	20.719196000
6	1.428336000	24.750189000	19.398279000
6	2.662597000	25.218015000	18.803608000
7	5.179095000	26.153270000	17.466521000
7	7.462864000	26.467537000	19.110081000
7	5.933471000	25.624034000	21.400065000
7	3.650941000	25.312185000	19.745407000
26	5.578788000	25.823494000	19.407909000
1	1.910445000	25.418021000	16.842020000
1	7.915250000	27.367655000	15.869028000
1	9.189185000	26.329269000	22.031059000
1	3.158826000	24.502424000	22.998269000
6	6.743194000	19.652103000	19.527795000
6	7.007912000	20.933691000	19.016611000
6	9.154865000	19.158037000	19.643450000
6	7.823858000	18.777849000	19.848016000
6	5.520907000	18.962067000	19.855361000
6	8.335371000	21.290511000	18.811519000
6	9.404027000	20.421249000	19.116748000
7	7.275141000	17.630816000	20.384852000
1	7.704784000	16.719072000	20.531328000
6	4.126254000	19.453306000	19.597687000
6	3.685587000	19.330597000	18.124627000
7	3.374250000	17.966056000	17.723010000
6	2.378948000	17.709073000	16.830810000
6	2.234672000	16.248829000	16.422686000
8	1.639603000	18.582151000	16.364519000
1	3.931923000	17.181054000	18.070566000
8	8.706016000	22.538035000	18.307392000
6	7.754561000	23.317586000	17.677347000
1	5.299640000	16.916257000	20.710324000
1	6.203941000	21.639456000	18.839045000
1	9.977488000	18.488640000	19.881129000
1	10.421420000	20.752584000	18.935162000
1	3.410653000	18.911455000	20.227974000
1	4.047701000	20.512753000	19.877052000
1	4.474260000	19.747113000	17.480017000
1	2.785043000	19.921675000	17.947457000
1	1.206346000	15.925615000	16.618309000
1	2.936439000	15.594298000	16.945689000
1	2.393275000	16.176806000	15.340505000
1	8.238671000	24.190798000	17.242815000
1	6.856526000	23.832926000	18.549820000
1	7.109526000	22.758879000	16.992625000
16	5.083848000	28.137327000	20.002058000
6	12.562770000	23.351906000	17.933519000
8	13.742240000	23.346964000	17.584849000
7	11.573397000	22.777850000	17.190682000
6	11.868745000	22.047406000	15.963043000
6	12.134131000	22.905917000	14.713228000
6	10.934851000	23.692170000	14.139175000
6	10.635007000	24.982808000	14.918345000
6	11.173008000	24.019444000	12.655670000
1	10.627210000	22.754373000	17.556947000
1	11.025735000	21.371182000	15.780269000
1	12.496830000	22.211623000	13.940762000
1	12.965923000	23.587949000	14.929837000
1	10.046100000	23.044103000	14.197660000
1	9.762856000	25.498493000	14.497018000
1	11.489746000	25.670812000	14.864777000
1	10.435779000	24.788345000	15.975101000
1	10.328578000	24.575592000	12.230328000
1	11.310197000	23.108371000	12.060440000
1	12.073426000	24.636598000	12.532573000
6	8.891848000	18.359814000	24.523979000
6	9.248233000	16.874157000	24.391480000
8	9.943621000	16.291047000	25.224025000
6	9.969959000	19.157774000	25.268340000
6	11.304890000	19.327282000	24.515295000
6	11.167401000	20.193047000	23.253130000
6	12.369733000	19.915224000	25.454165000
1	7.952154000	18.412647000	25.092228000
1	10.164903000	18.640651000	26.214896000
1	9.570427000	20.152094000	25.516035000
1	11.643049000	18.325295000	24.216260000
1	12.131939000	20.290986000	22.739268000
1	10.455056000	19.778555000	22.531939000
1	10.825209000	21.205085000	23.508818000
1	13.336848000	20.017313000	24.945979000
1	12.516312000	19.279224000	26.335481000
1	12.074466000	20.913359000	25.806404000
7	8.712569000	16.256363000	23.305088000
6	8.833881000	14.829610000	23.042894000
6	7.522757000	14.251050000	22.495602000
8	7.238651000	14.949132000	21.293312000
6	6.370344000	14.331651000	23.503031000
1	8.134949000	16.786754000	22.664576000
1	9.123026000	14.344826000	23.978985000
1	7.715770000	13.185787000	22.273044000
1	6.285832000	14.767238000	21.011968000
1	5.443344000	13.987395000	23.037473000
1	6.217917000	15.364540000	23.835218000
1	6.585171000	13.714258000	24.384766000
6	1.912136000	14.227918000	21.216099000
6	0.397245000	14.106061000	21.184308000
8	-0.193233000	13.089490000	20.841235000
6	2.462275000	14.692885000	19.855016000
6	3.999122000	14.891444000	19.806838000
8	4.646336000	14.559957000	20.849721000
8	4.469106000	15.379639000	18.753010000
1	2.236986000	14.924662000	21.997595000
1	2.184659000	13.970787000	19.077449000
1	2.003682000	15.644502000	19.555160000
7	-0.289391000	15.253090000	21.567399000
6	-1.708774000	15.456449000	21.240837000
6	-2.634058000	15.075782000	22.415622000
8	-2.077355000	15.589237000	23.643704000
6	-2.906537000	13.578928000	22.547641000
1	0.271901000	16.095170000	21.596712000
1	-1.971976000	14.883001000	20.348425000
1	-3.579933000	15.609370000	22.276802000
1	-1.124716000	15.382414000	23.574186000
1	-3.536498000	13.391312000	23.424497000
1	-1.975534000	13.013136000	22.643177000
1	-3.423570000	13.207700000	21.655476000
6	-2.907391000	21.763297000	22.556979000
6	-2.106083000	23.048224000	22.296224000
8	-0.883730000	23.096743000	22.485035000
6	-2.126400000	20.765045000	23.409095000
6	-2.461459000	19.303578000	23.134333000

8	-2.901409000	18.903370000	22.047576000
7	-2.213113000	18.443722000	24.148163000
1	-3.877926000	22.008238000	23.004130000
1	-2.243600000	20.980581000	24.478381000
1	-1.060236000	20.910205000	23.186978000
1	-1.776790000	18.774538000	24.996099000
1	-2.241779000	17.431941000	23.972240000
7	-2.832931000	24.081243000	21.828796000
6	-2.214098000	25.297428000	21.308127000
6	-3.289309000	26.332786000	20.941608000
6	-4.338463000	25.771170000	19.956533000
8	-4.409418000	26.313818000	18.805935000
8	-5.042225000	24.809401000	20.355854000
1	-3.806877000	23.963785000	21.529986000
1	-1.607001000	25.065103000	20.422491000
1	-2.796821000	27.208240000	20.510259000
1	-3.815830000	26.642460000	21.854045000
6	-2.612753000	20.340046000	18.022662000
6	-3.954859000	19.692135000	18.306832000
8	-4.856335000	19.715763000	17.424032000
6	-1.412196000	19.964038000	18.909219000
6	-0.233041000	20.928820000	18.650875000
6	-0.998257000	18.496680000	18.696058000
6	0.769063000	20.968316000	19.809674000
1	-2.393436000	20.121431000	16.969887000
1	-1.701745000	20.099235000	19.961779000
1	0.274106000	20.629398000	17.724386000
1	-0.617996000	21.945128000	18.489462000
1	-0.214589000	18.204963000	19.404889000
1	-0.586117000	18.360328000	17.689772000
1	-1.839252000	17.806309000	18.828829000
1	1.617450000	21.617689000	19.571206000
1	0.302759000	21.367523000	20.718660000
1	1.167162000	19.972341000	20.034295000
7	-4.168185000	19.161927000	19.521120000
6	-5.320702000	18.335601000	19.910970000
6	-5.301023000	17.034463000	19.059138000
8	-4.342802000	16.271070000	19.120501000
6	-6.669606000	19.094455000	20.001601000
8	-7.226656000	19.412231000	18.717854000
6	-6.580713000	20.339603000	20.883431000
1	-3.423893000	19.180767000	20.215697000
1	-5.074394000	18.013249000	20.926621000
1	-7.383368000	18.393784000	20.455026000
1	-6.474531000	19.725407000	18.161204000
1	-7.565918000	20.810672000	20.943661000
1	-5.879739000	21.071124000	20.470118000
1	-6.245973000	20.081241000	21.895258000
7	-6.387467000	16.832331000	18.263814000
6	-6.417038000	15.829010000	17.218784000
6	-6.654891000	16.411293000	15.816445000
8	-7.156958000	15.723636000	14.935441000
1	-7.050710000	17.605428000	18.219315000
1	-5.456275000	15.303388000	17.238756000
7	-6.287516000	17.720290000	15.647198000
6	-6.370789000	18.373531000	14.353794000
6	-7.309290000	19.584686000	14.285189000
8	-7.246194000	20.370234000	13.338749000
1	-5.727663000	18.183434000	16.360562000
1	-5.384767000	18.714542000	14.024754000
1	-6.727928000	17.629913000	13.634387000
7	-8.221926000	19.696925000	15.285707000
6	-9.158960000	20.807044000	15.361338000
6	-8.701227000	21.905518000	16.337071000
8	-9.312084000	22.131801000	17.377564000
1	-8.123158000	19.091369000	16.090119000
1	-9.261647000	21.204214000	14.347333000
7	-7.562240000	22.548644000	15.965519000
6	-6.959718000	23.576692000	16.796468000
6	-6.360364000	24.653881000	15.880808000
8	-6.377982000	24.534442000	14.647570000
6	-5.873886000	22.996149000	17.723182000
8	-4.709723000	22.623792000	16.981083000
1	-7.220204000	22.475958000	15.012615000
1	-7.747451000	24.011142000	17.422279000
1	-5.574444000	23.723141000	18.484309000
1	-6.303119000	22.131592000	18.242267000
1	-4.808615000	21.692654000	16.710742000
7	-5.809915000	25.710116000	16.518951000
6	-5.191697000	26.795547000	15.788475000
6	-3.746674000	26.582812000	15.322102000
8	-3.112440000	27.536199000	14.866608000
1	-5.579213000	25.708861000	17.526951000
1	-5.774811000	27.004305000	14.885769000
7	-3.258840000	25.319771000	15.402370000
6	-2.010517000	24.923850000	14.763808000
6	-2.243259000	24.348743000	13.347403000
8	-1.302677000	24.268238000	12.555856000
6	-1.193283000	24.025194000	15.738319000
8	-0.571455000	24.832599000	16.729201000
6	-0.090758000	23.203699000	15.079759000
1	-3.785277000	24.614752000	15.910648000
1	-1.427295000	25.832593000	14.596522000
1	-1.909525000	23.330766000	16.210960000
1	-1.186449000	25.521653000	17.091204000
1	0.483012000	22.701035000	15.864468000
1	0.581260000	23.842521000	14.499813000
1	-0.500568000	22.444473000	14.408801000
7	-3.514450000	23.970501000	13.042664000
6	-3.919604000	23.606280000	11.690927000
6	-4.502699000	22.192965000	11.555740000
6	-3.552719000	21.013279000	11.697659000
6	-4.105050000	19.721107000	11.703105000
6	-2.161089000	21.141290000	11.792626000
6	-3.293866000	18.590355000	11.797382000
6	-1.348146000	20.006400000	11.896214000
6	-1.906375000	18.729040000	11.896927000
1	-4.258778000	24.176736000	13.701569000
1	-3.045307000	23.741615000	11.050152000
1	-5.329380000	22.071349000	12.268179000
1	-4.973544000	22.139576000	10.561741000
1	-5.186231000	19.613539000	11.651371000
1	-1.696988000	22.123108000	11.807057000
1	-3.744743000	17.600156000	11.800258000
1	-0.271787000	20.129215000	11.986454000
1	-1.270545000	17.851820000	11.984928000
1	7.838765000	25.537933000	24.144533000
1	5.270290000	24.771935000	24.560410000
1	10.054274000	27.694251000	17.391455000
1	10.584693000	27.241314000	20.013204000
1	3.257108000	26.231828000	14.736412000
1	5.807809000	27.062830000	14.311039000
1	1.006499000	24.191426000	21.484456000
1	0.517122000	24.594800000	18.834262000
1	8.673901000	18.792350000	23.540371000
1	9.631518000	14.648004000	22.308077000
1	2.328563000	13.248494000	21.461730000
1	-1.847947000	16.517433000	21.023879000
1	-3.125605000	21.306275000	21.584702000
1	-1.529163000	25.701917000	22.061075000
1	-4.685791000	24.319727000	11.359196000
1	-2.803316000	21.421472000	18.062794000
1	12.757013000	21.432318000	16.146733000
1	-5.201195000	27.687846000	16.418141000
1	-10.128347000	20.447669000	15.714427000
8	-1.940899000	26.984039000	17.796845000
1	-2.102646000	27.528430000	17.008034000
1	-2.843954000	26.786105000	18.160064000
1	-7.205522000	15.086860000	17.384282000
6	5.897474000	17.745062000	20.362748000
8	5.946036000	24.115974000	19.236451000
6	12.102699000	24.021589000	19.221788000
1	12.769169000	23.714633000	20.033166000
1	12.195385000	25.107474000	19.110250000
1	11.067990000	23.789729000	19.489633000
1	3.929865000	27.920168000	20.671726000

$TS1^{4}_{HA}$

6	2.610405000	25.668942000	17.298244000

6	7.166307000	27.057609000	16.399126000
6	8.423152000	25.885762000	20.921478000
6	3.842164000	24.602674000	21.849443000
6	3.744468000	26.171916000	16.667604000
6	3.781229000	26.625762000	15.298899000
6	5.061633000	27.015438000	15.049086000
6	5.809869000	26.801828000	16.264237000
6	7.919983000	26.848444000	17.549868000
6	9.325913000	27.145093000	17.678900000
6	9.683455000	26.806052000	18.951020000
6	8.494123000	26.305304000	19.596581000
6	7.273694000	25.458759000	21.571681000
6	7.232479000	25.032041000	22.948597000
6	5.943525000	24.681225000	23.214757000
6	5.195906000	24.889366000	21.999387000
6	3.123622000	24.745010000	20.675355000
6	1.713741000	24.444940000	20.538586000
6	1.377783000	24.738186000	19.254844000
6	2.576740000	25.221868000	18.607783000
7	4.981736000	26.294464000	17.239229000
7	7.439433000	26.333526000	18.723981000
7	6.016168000	25.375873000	21.018819000
7	3.636422000	25.204100000	19.483689000
26	5.505170000	25.730504000	19.104635000
1	1.678144000	25.626959000	16.741747000
1	7.682105000	27.463428000	15.533693000
1	9.341887000	25.905487000	21.500267000
1	3.309805000	24.223819000	22.716279000
6	6.786688000	19.716877000	19.425224000
6	7.109969000	20.972808000	18.885577000
6	9.178292000	19.172253000	19.684810000
6	7.829113000	18.828651000	19.827100000
6	5.533204000	19.066985000	19.716823000
6	8.455809000	21.294908000	18.745739000
6	9.485828000	20.413346000	19.138435000
7	7.228194000	17.712292000	20.368116000
1	7.629570000	16.796961000	20.571871000
6	4.162410000	19.584301000	19.389383000
6	3.749274000	19.377095000	17.917545000
7	3.397767000	18.000856000	17.597471000
6	2.371685000	17.717930000	16.747762000
6	2.194997000	16.242261000	16.414876000
8	1.633087000	18.578614000	16.259029000
1	3.946948000	17.223597000	17.972779000
8	8.880568000	22.516736000	18.226743000
6	8.005575000	23.258578000	17.452156000
1	5.224433000	17.055229000	20.626067000
1	6.334671000	21.687763000	18.631589000
1	9.969835000	18.491172000	19.986235000
1	10.519008000	20.716897000	19.005052000
1	3.414323000	19.110755000	20.035998000
1	4.114647000	20.662089000	19.596064000
1	4.565917000	19.722660000	17.265852000
1	2.872379000	19.982939000	17.680600000
1	1.170000000	15.942291000	16.659062000
1	2.905444000	15.602801000	16.944681000
1	2.316110000	16.118030000	15.332594000
1	8.541571000	24.104903000	17.025699000
1	6.972372000	23.823427000	18.162870000
1	7.446922000	22.655917000	16.728905000
16	5.173946000	28.033486000	19.618607000
6	12.776292000	23.238097000	18.626675000
8	14.000501000	23.200651000	18.518005000
7	11.938111000	22.761623000	17.660719000
6	12.454516000	22.069905000	16.484098000
6	12.999947000	22.967702000	15.358410000
6	11.968165000	23.766663000	14.534523000
6	11.420053000	24.994611000	15.278286000
6	12.581628000	24.198438000	13.192129000
1	10.937598000	22.759438000	17.830658000
1	11.647145000	21.435118000	16.100777000
1	13.543422000	22.299534000	14.673788000
1	13.749712000	23.644090000	15.787647000
1	11.122305000	23.096735000	14.312306000
1	10.682028000	25.524840000	14.663280000
1	12.231222000	25.698641000	15.508798000
1	10.941882000	24.728959000	16.224012000
1	11.859511000	24.761095000	12.587642000
1	12.911910000	23.333297000	12.604066000
1	13.455084000	24.844773000	13.353102000
6	8.926507000	18.559054000	24.473258000
6	9.251963000	17.063146000	24.379063000
8	9.947923000	16.490558000	25.218185000
6	10.022134000	19.353226000	25.195925000
6	11.355181000	19.486079000	24.432063000
6	11.227050000	20.345295000	23.164198000
6	12.440777000	20.056691000	25.357615000
1	7.988703000	18.646058000	25.040441000
1	10.213988000	18.850790000	26.150890000
1	9.640902000	20.358604000	25.427245000
1	11.667979000	18.474439000	24.137996000
1	12.188537000	20.412908000	22.639485000
1	10.495577000	19.944834000	22.454406000
1	10.915003000	21.368013000	23.416713000
1	13.405160000	20.134807000	24.840219000
1	12.581333000	19.422930000	26.241451000
1	12.170290000	21.063100000	25.706247000
7	8.687656000	16.423732000	23.319752000
6	8.781800000	14.987335000	23.099447000
6	7.462928000	14.414978000	22.565370000
8	7.202180000	15.066582000	21.331993000
6	6.303520000	14.560140000	23.557472000
1	8.114069000	16.947549000	22.670539000
1	9.059131000	14.525308000	24.050541000
1	7.635783000	13.337889000	22.388614000
1	6.250775000	14.881744000	21.043900000
1	5.375031000	14.212422000	23.097361000
1	6.167219000	15.608922000	23.843615000
1	6.498802000	13.977529000	24.466941000
6	1.901553000	14.227752000	21.188341000
6	0.388489000	14.087536000	21.149435000
8	-0.188771000	13.066998000	20.795668000
6	2.450319000	14.721813000	19.837203000
6	3.982528000	14.955602000	19.802143000
8	4.622328000	14.668158000	20.862990000
8	4.454486000	15.427256000	18.742187000
1	2.215678000	14.916273000	21.981173000
1	2.193151000	14.005561000	19.047644000
1	1.973128000	15.667620000	19.547310000
7	-0.312165000	15.223203000	21.539514000
6	-1.732667000	15.415118000	21.211431000
6	-2.655733000	15.015004000	22.381868000
8	-2.101734000	15.516736000	23.616210000
6	-2.918343000	13.514835000	22.495191000
1	0.239833000	16.070737000	21.582917000
1	-1.987989000	14.845608000	20.314231000
1	-3.605067000	15.544023000	22.250046000
1	-1.148780000	15.311503000	23.545460000
1	-3.544592000	13.311404000	23.371167000
1	-1.983281000	12.954003000	22.580241000
1	-3.435646000	13.152412000	21.599554000
6	-2.874762000	21.687578000	22.512082000
6	-2.073406000	22.963700000	22.210620000
8	-0.844119000	23.001564000	22.348877000
6	-2.071802000	20.690533000	23.345183000
6	-2.423357000	19.230601000	23.086412000
8	-2.868710000	18.826097000	22.003156000
7	-2.183506000	18.376190000	24.106545000
1	-3.827224000	21.943747000	22.990734000
1	-2.153545000	20.912717000	24.416331000
1	-1.012952000	20.829205000	23.086594000
1	-1.747992000	18.708562000	24.954171000
1	-2.232551000	17.363433000	23.940790000
7	-2.811761000	23.998478000	21.766719000
6	-2.213660000	25.211148000	21.215321000
6	-3.297392000	26.266833000	20.943768000
6	-4.416020000	25.738003000	20.018544000
8	-4.564871000	26.312680000	18.891126000
8	-5.095717000	24.766997000	20.437535000
1	-3.801369000	23.889892000	21.521262000

1	-1.679641000	24.980890000	20.282949000
1	-2.823695000	27.146993000	20.501186000
1	-3.757950000	26.560540000	21.896321000
6	-2.724148000	20.292115000	17.976653000
6	-4.055269000	19.642349000	18.306034000
8	-4.988956000	19.672856000	17.458012000
6	-1.487631000	19.897000000	18.804166000
6	-0.316493000	20.862183000	18.512022000
6	-1.088549000	18.431946000	18.550671000
6	0.732903000	20.879085000	19.628197000
1	-2.550989000	20.092780000	16.911578000
1	-1.730853000	20.015262000	19.870419000
1	0.153057000	20.577125000	17.561711000
1	-0.704745000	21.882184000	18.385114000
1	-0.274387000	18.127986000	19.218518000
1	-0.721958000	18.308896000	17.525101000
1	-1.924657000	17.741455000	18.711271000
1	1.570836000	21.532716000	19.365963000
1	0.305114000	21.260430000	20.563433000
1	1.139161000	19.878627000	19.814706000
7	-4.223066000	19.104193000	19.524246000
6	-5.359182000	18.273882000	19.953163000
6	-5.365658000	16.974584000	19.098793000
8	-4.407060000	16.209701000	19.130931000
6	-6.706757000	19.027292000	20.093471000
8	-7.308873000	19.349758000	18.831529000
6	-6.592348000	20.267355000	20.979604000
1	-3.452089000	19.117419000	20.189170000
1	-5.075868000	17.950529000	20.958712000
1	-7.401995000	18.321360000	20.567094000
1	-6.578078000	19.670789000	18.251180000
1	-7.575119000	20.738758000	21.069740000
1	-5.901768000	21.000508000	20.551891000
1	-6.230624000	20.002728000	21.980503000
7	-6.474021000	16.776545000	18.333391000
6	-6.534513000	15.777333000	17.285905000
6	-6.814333000	16.365241000	15.893629000
8	-7.336580000	15.678945000	15.023308000
1	-7.139547000	17.548868000	18.313015000
1	-5.573432000	15.252067000	17.275012000
7	-6.459635000	17.677218000	15.721403000
6	-6.581461000	18.337296000	14.434648000
6	-7.524620000	19.546229000	14.399838000
8	-7.487451000	20.339336000	13.458481000
1	-5.882609000	18.140205000	16.421084000
1	-5.606026000	18.684017000	14.081059000
1	-6.956279000	17.596640000	13.721204000
7	-8.411643000	19.647503000	15.424407000
6	-9.350437000	20.753089000	15.533631000
6	-8.877793000	21.840138000	16.515045000
8	-9.468847000	22.049787000	17.570240000
1	-8.291733000	19.034105000	16.219901000
1	-9.473788000	21.163443000	14.527196000
7	-7.748903000	22.493193000	16.129183000
6	-7.142492000	23.523893000	16.954380000
6	-6.594408000	24.623355000	16.033379000
8	-6.643089000	24.513690000	14.800025000
6	-6.020485000	22.954112000	17.844038000
8	-4.872714000	22.602287000	17.067657000
1	-7.432497000	22.440120000	15.166118000
1	-7.920566000	23.935313000	17.607334000
1	-5.708111000	23.681169000	18.600085000
1	-6.423052000	22.080824000	18.370115000
1	-4.976933000	21.678176000	16.776333000
7	-6.055029000	25.689777000	16.664345000
6	-5.491126000	26.797514000	15.923089000
6	-4.059102000	26.626184000	15.403735000
8	-3.469097000	27.596850000	14.925311000
1	-5.789558000	25.686438000	17.663548000
1	-6.111858000	27.000520000	15.044592000
7	-3.531739000	25.379056000	15.471194000
6	-2.287641000	25.017227000	14.804965000
6	-2.536142000	24.432096000	13.395614000
8	-1.610544000	24.370537000	12.585017000
6	-1.424846000	24.144482000	15.764403000
8	-0.796353000	24.972071000	16.733403000
6	-0.321264000	23.342299000	15.083922000
1	-4.022450000	24.660808000	15.995891000
1	-1.733328000	25.941070000	14.622447000
1	-2.113830000	23.437549000	16.259097000
1	-1.411608000	25.659704000	17.098130000
1	0.281784000	22.857512000	15.857884000
1	0.323704000	23.991072000	14.484857000
1	-0.731119000	22.570174000	14.427762000
7	-3.804344000	24.023881000	13.117601000
6	-4.227214000	23.646087000	11.775066000
6	-4.787443000	22.221990000	11.656608000
6	-3.813969000	21.060279000	11.787288000
6	-4.341101000	19.757680000	11.795485000
6	-2.424423000	21.215348000	11.871325000
6	-3.507395000	18.642903000	11.883474000
6	-1.588754000	20.096507000	11.968755000
6	-2.122157000	18.808592000	11.973665000
1	-4.539466000	24.212571000	13.791935000
1	-3.366689000	23.793568000	11.118520000
1	-5.599197000	22.089289000	12.384233000
1	-5.275005000	22.155368000	10.671588000
1	-5.420302000	19.629186000	11.750317000
1	-1.979563000	22.206049000	11.880010000
1	-3.938743000	17.644035000	11.888312000
1	-0.514301000	20.240035000	12.050548000
1	-1.468679000	17.943977000	12.056869000
1	8.090863000	25.001512000	23.608138000
1	5.522503000	24.301947000	24.137317000
1	9.943993000	27.561010000	16.893222000
1	10.654032000	26.889986000	19.423486000
1	2.927598000	26.629442000	14.633370000
1	5.480554000	27.408748000	14.131158000
1	1.074024000	24.057156000	21.323420000
1	0.426035000	24.649407000	18.745754000
1	8.716099000	18.970157000	23.478757000
1	9.577958000	14.768428000	22.373397000
1	2.328846000	13.249578000	21.420381000
1	-1.882038000	16.476406000	21.002965000
1	-3.129392000	21.223516000	21.552188000
1	-1.467496000	25.594084000	21.919213000
1	-5.011525000	24.344334000	11.453273000
1	-2.909695000	21.373047000	18.045585000
1	13.266769000	21.413409000	16.815804000
1	-5.499887000	27.682762000	16.562650000
1	-10.311875000	20.385885000	15.900330000
8	-2.179456000	27.114860000	17.793687000
1	-2.393488000	27.637211000	17.002317000
1	-3.058971000	26.877741000	18.190756000
1	-7.317320000	15.033977000	17.471901000
6	5.856637000	17.858433000	20.279326000
8	5.918449000	24.120107000	18.622046000
6	12.089330000	23.795403000	19.866570000
1	12.602553000	24.712517000	20.169492000
1	11.025054000	23.999198000	19.719086000
1	12.193643000	23.073516000	20.685129000
1	5.872136000	28.063704000	20.776258000

$IM1^{2}_{HA}$

6	2.349327000	25.943052000	17.498171000
6	6.947754000	27.166931000	16.623989000
6	8.092143000	26.240497000	21.229466000
6	3.527740000	24.853060000	22.046810000
6	3.500391000	26.414362000	16.874147000
6	3.565150000	26.821973000	15.489430000
6	4.859190000	27.157637000	15.242191000
6	5.585105000	26.960360000	16.476383000
6	7.679297000	27.001296000	17.799120000
6	9.084370000	27.294588000	17.941536000
6	9.405030000	27.048266000	19.245021000
6	8.196324000	26.597912000	19.887895000
6	6.942034000	25.784412000	21.865317000
6	6.873407000	25.384075000	23.249209000
6	5.589781000	24.985457000	23.474302000

6	4.881391000	25.150069000	22.227777000
6	2.804395000	25.014715000	20.874903000
6	1.399055000	24.689050000	20.722104000
6	1.073077000	24.998639000	19.439181000
6	2.277764000	25.510301000	18.819754000
7	4.734483000	26.522792000	17.464897000
7	7.158564000	26.568171000	18.988753000
7	5.717804000	25.635938000	21.265224000
7	3.318093000	25.512045000	19.703944000
26	5.223303000	26.010844000	19.351007000
1	1.429459000	25.891074000	16.920393000
1	7.491863000	27.510233000	15.748301000
1	8.994668000	26.309182000	21.830539000
1	2.991056000	24.457329000	22.904335000
6	6.984501000	19.743124000	19.638538000
6	7.389654000	20.989409000	19.128218000
6	9.333925000	19.013120000	19.819411000
6	7.967154000	18.770189000	19.990900000
6	5.693216000	19.176223000	19.936390000
6	8.751053000	21.206617000	18.957378000
6	9.720976000	20.241078000	19.292603000
7	7.298974000	17.684632000	20.517612000
1	7.638351000	16.733016000	20.672095000
6	4.355175000	19.790161000	19.636516000
6	3.923621000	19.647797000	18.162582000
7	3.539827000	18.291466000	17.794929000
6	2.527333000	18.062926000	16.912894000
6	2.327237000	16.605897000	16.517482000
8	1.816932000	18.957606000	16.443995000
1	4.075437000	17.490740000	18.137270000
8	9.266087000	22.412531000	18.464277000
6	8.443843000	23.288845000	17.828608000
1	5.259392000	17.162369000	20.789138000
1	6.659732000	21.762375000	18.909680000
1	10.078365000	18.266766000	20.083909000
1	10.770743000	20.474969000	19.144785000
1	3.582489000	19.346835000	20.276356000
1	4.375743000	20.863905000	19.867820000
1	4.741632000	19.999919000	17.516307000
1	3.059539000	20.283006000	17.957884000
1	1.292018000	16.317752000	16.731133000
1	3.015062000	15.930581000	17.032404000
1	2.465134000	16.523541000	15.433200000
1	8.937780000	24.213094000	17.558949000
1	6.528205000	24.180379000	18.961319000
1	7.602368000	22.899453000	17.266247000
16	4.838975000	28.367283000	19.808763000
6	13.046850000	23.289171000	18.054378000
8	14.252968000	23.330192000	17.817327000
7	12.195655000	22.449171000	17.399000000
6	12.683002000	21.493387000	16.410035000
6	13.015805000	22.074518000	15.023218000
6	11.830817000	22.429282000	14.101413000
6	11.026869000	23.650442000	14.575121000
6	12.334941000	22.656906000	12.666361000
1	11.221951000	22.417628000	17.680100000
1	11.928156000	20.703560000	16.319825000
1	13.628303000	21.317953000	14.510577000
1	13.663805000	22.948874000	15.163148000
1	11.150621000	21.563034000	14.081023000
1	10.229834000	23.889853000	13.860322000
1	11.675364000	24.532323000	14.662540000
1	10.559024000	23.485872000	15.549424000
1	11.505066000	22.879707000	11.984708000
1	12.860756000	21.774876000	12.280286000
1	13.033414000	23.503722000	12.628133000
6	9.184380000	18.328117000	24.585602000
6	9.432688000	16.822592000	24.428880000
8	10.107165000	16.184508000	25.237857000
6	10.335409000	19.039522000	25.308563000
6	11.660726000	19.118155000	24.524298000
6	11.555718000	20.002533000	23.272061000
6	12.788187000	19.618484000	25.440409000
1	8.266380000	18.440023000	25.180126000
1	10.514949000	18.501818000	26.246601000
1	10.015175000	20.058038000	25.572735000
1	11.917061000	18.096772000	24.210045000
1	12.511519000	20.033948000	22.734167000
1	10.796473000	19.647656000	22.567161000
1	11.295886000	21.034822000	23.543743000
1	13.747435000	19.654592000	24.908927000
1	12.909551000	18.965320000	26.312956000
1	12.575452000	20.632175000	25.807505000
7	8.828190000	16.253137000	23.351604000
6	8.860183000	14.823841000	23.072335000
6	7.512206000	14.316619000	22.545440000
8	7.260152000	14.999184000	21.327260000
6	6.372265000	14.486210000	23.555563000
1	8.279766000	16.831267000	22.727052000
1	9.139543000	14.315867000	23.999040000
1	7.640835000	13.237290000	22.345853000
1	6.296704000	14.857773000	21.049428000
1	5.426162000	14.178240000	23.103022000
1	6.276874000	15.534359000	23.859964000
1	6.558939000	13.882762000	24.453172000
6	1.945859000	14.274911000	21.146411000
6	0.433443000	14.139641000	21.082762000
8	-0.142233000	13.131447000	20.693221000
6	2.511466000	14.848379000	19.834143000
6	4.045842000	15.070733000	19.830186000
8	4.671379000	14.699877000	20.873728000
8	4.532856000	15.612376000	18.811749000
1	2.249867000	14.915361000	21.982481000
1	2.258707000	14.184165000	18.998793000
1	2.044077000	15.813612000	19.597789000
7	-0.269366000	15.266825000	21.494692000
6	-1.682536000	15.473936000	21.144593000
6	-2.628652000	15.047242000	22.286790000
8	-2.094363000	15.512405000	23.543870000
6	-2.900629000	13.546050000	22.354179000
1	0.285436000	16.110775000	21.565658000
1	-1.923666000	14.930493000	20.227681000
1	-3.572848000	15.585093000	22.153054000
1	-1.139854000	15.311378000	23.480859000
1	-3.542630000	13.321648000	23.213487000
1	-1.969797000	12.978262000	22.439160000
1	-3.404300000	13.211556000	21.440074000
6	-3.037456000	21.676571000	22.579284000
6	-2.284288000	22.991644000	22.325683000
8	-1.060484000	23.076702000	22.489086000
6	-2.208250000	20.690009000	23.399693000
6	-2.500316000	19.224833000	23.099038000
8	-2.908646000	18.832516000	21.996764000
7	-2.250099000	18.354175000	24.102585000
1	-4.006733000	21.881779000	23.048646000
1	-2.313791000	20.882099000	24.474534000
1	-1.151885000	20.874751000	23.160780000
1	-1.847284000	18.680463000	24.968501000
1	-2.263146000	17.344346000	23.912848000
7	-3.055470000	24.008595000	21.894548000
6	-2.490266000	25.255186000	21.385630000
6	-3.605778000	26.266685000	21.077307000
6	-4.664445000	25.701866000	20.104277000
8	-4.796627000	26.284994000	18.979106000
8	-5.316957000	24.698266000	20.487354000
1	-4.031289000	23.864666000	21.614030000
1	-1.904619000	25.061789000	20.476451000
1	-3.151718000	27.169676000	20.660607000
1	-4.116305000	26.533341000	22.012192000
6	-2.661786000	20.417136000	18.016618000
6	-3.977386000	19.705820000	18.276846000
8	-4.878160000	19.717714000	17.393366000
6	-1.437430000	20.033248000	18.868580000
6	-0.288655000	21.042390000	18.641961000
6	-0.986230000	18.590326000	18.578172000
6	0.741458000	21.044054000	19.776802000
1	-2.447665000	20.265640000	16.951254000
1	-1.715844000	20.107933000	19.930321000
1	0.208962000	20.811464000	17.691449000
1	-0.704895000	22.055204000	18.549982000

1	-0.183761000	18.287210000	19.260246000
1	-0.584727000	18.516240000	17.561122000
1	-1.805246000	17.870014000	18.687411000
1	1.561296000	21.735245000	19.555957000
1	0.285806000	21.370273000	20.720004000
1	1.178898000	20.050317000	19.926314000
7	-4.171497000	19.132178000	19.474840000
6	-5.290325000	18.247397000	19.834364000
6	-5.218657000	16.976526000	18.940808000
8	-4.226793000	16.255529000	18.971867000
6	-6.669361000	18.946380000	19.947567000
8	-7.238808000	19.282202000	18.673791000
6	-6.632638000	20.163475000	20.871091000
1	-3.428874000	19.157073000	20.171131000
1	-5.032142000	17.902274000	20.839578000
1	-7.353256000	18.201886000	20.376578000
1	-6.499662000	19.639336000	18.126410000
1	-7.635984000	20.592711000	20.943460000
1	-5.959753000	20.935539000	20.485927000
1	-6.291072000	19.883874000	21.874982000
7	-6.301245000	16.748134000	18.146998000
6	-6.290190000	15.775641000	17.072576000
6	-6.552833000	16.389009000	15.688163000
8	-7.027955000	15.708822000	14.786686000
1	-6.997800000	17.492283000	18.126150000
1	-5.308343000	15.290284000	17.077673000
7	-6.239756000	17.716462000	15.558914000
6	-6.357281000	18.407536000	14.288315000
6	-7.357081000	19.570293000	14.262348000
8	-7.325186000	20.401591000	13.354123000
1	-5.698494000	18.180980000	16.285452000
1	-5.390562000	18.810324000	13.972621000
1	-6.676716000	17.671500000	13.543730000
7	-8.286834000	19.586831000	15.253256000
6	-9.277567000	20.645442000	15.370126000
6	-8.876470000	21.729342000	16.386472000
8	-9.514953000	21.904837000	17.419862000
1	-8.159421000	18.956471000	16.034240000
1	-9.400095000	21.076388000	14.372175000
7	-7.753368000	22.421082000	16.053837000
6	-7.211808000	23.458469000	16.914689000
6	-6.703650000	24.608703000	16.033524000
8	-6.731359000	24.534335000	14.797154000
6	-6.080009000	22.922052000	17.812599000
8	-4.899768000	22.648500000	17.053013000
1	-7.403290000	22.392198000	15.101403000
1	-8.021570000	23.813397000	17.562223000
1	-5.821658000	23.643267000	18.594055000
1	-6.448840000	22.016358000	18.307148000
1	-4.939978000	21.721534000	16.754867000
7	-6.221886000	25.679162000	16.703657000
6	-5.699321000	26.833099000	16.003995000
6	-4.255593000	26.740318000	15.497319000
8	-3.699572000	27.751263000	15.063597000
1	-5.968548000	25.655369000	17.705602000
1	-6.318765000	27.036492000	15.124575000
7	-3.679760000	25.513143000	15.525476000
6	-2.412885000	25.223681000	14.867338000
6	-2.614628000	24.659147000	13.442196000
8	-1.675371000	24.660550000	12.644929000
6	-1.520605000	24.373796000	15.820908000
8	-0.932036000	25.216969000	16.802268000
6	-0.382045000	23.623785000	15.138985000
1	-4.146359000	24.758904000	16.020972000
1	-1.900504000	26.176243000	14.711571000
1	-2.182561000	23.634839000	16.304913000
1	-1.586777000	25.852628000	17.192238000
1	0.234493000	23.152750000	15.910920000
1	0.240427000	24.303646000	14.550690000
1	-0.758084000	22.844487000	14.471033000
7	-3.858088000	24.199434000	13.135221000
6	-4.244314000	23.838080000	11.776849000
6	-4.728558000	22.390844000	11.610848000
6	-3.697228000	21.277394000	11.715687000
6	-4.156457000	19.949524000	11.686949000
6	-2.317709000	21.501384000	11.809224000
6	-3.266815000	18.877139000	11.749392000
6	-1.425750000	20.424793000	11.879435000
6	-1.892087000	19.111485000	11.848422000
1	-4.610994000	24.339060000	13.801978000
1	-3.385091000	24.048839000	11.136031000
1	-5.537884000	22.194291000	12.326363000
1	-5.203856000	22.329061000	10.619486000
1	-5.227385000	19.767394000	11.632444000
1	-1.924773000	22.513156000	11.847305000
1	-3.646202000	17.857665000	11.727056000
1	-0.360223000	20.621316000	11.968073000
1	-1.195261000	18.279539000	11.910661000
1	7.706492000	25.402354000	23.940691000
1	5.149271000	24.610790000	24.389589000
1	9.725059000	27.654084000	17.145855000
1	10.363187000	27.161062000	19.736745000
1	2.720272000	26.828393000	14.812884000
1	5.302377000	27.501625000	14.315983000
1	0.760881000	24.266879000	21.490367000
1	0.132669000	24.893581000	18.913411000
1	8.971287000	18.786518000	23.612647000
1	9.632251000	14.603986000	22.321087000
1	2.369860000	13.284510000	21.325657000
1	-1.823462000	16.541036000	20.961857000
1	-3.258877000	21.228223000	21.603632000
1	-1.794228000	25.662010000	22.126856000
1	-5.060237000	24.503674000	11.464829000
1	-2.888451000	21.487691000	18.119804000
1	13.594775000	21.039054000	16.813731000
1	-5.754021000	27.696839000	16.670192000
1	-10.227924000	20.225100000	15.706967000
8	-2.437238000	27.228722000	17.942538000
1	-2.671582000	27.768334000	17.168610000
1	-3.307047000	26.933322000	18.322402000
1	-7.047212000	14.997110000	17.215974000
6	5.941298000	17.931042000	20.458464000
8	5.573990000	24.316928000	18.812224000
6	12.392884000	24.199575000	19.086541000
1	13.044894000	24.259124000	19.962261000
1	12.304876000	25.209592000	18.669144000
1	11.397030000	23.864360000	19.389881000
1	3.554489000	28.438348000	19.392034000

IM1$^{4}_{HA}$

6	2.363843000	25.869909000	17.480019000
6	6.944814000	27.218289000	16.632088000
6	8.082027000	26.275660000	21.244486000
6	3.528092000	24.825227000	22.059096000
6	3.505512000	26.372944000	16.871203000
6	3.574331000	26.777882000	15.489929000
6	4.862674000	27.148937000	15.250653000
6	5.586791000	26.976549000	16.485672000
6	7.668157000	27.036074000	17.804310000
6	9.065144000	27.351510000	17.963526000
6	9.383240000	27.090774000	19.264757000
6	8.182703000	26.613190000	19.901249000
6	6.923735000	25.844004000	21.874058000
6	6.852759000	25.445286000	23.257098000
6	5.574626000	25.032801000	23.485968000
6	4.858229000	25.180185000	22.244694000
6	2.837312000	24.938474000	20.864755000
6	1.441382000	24.600538000	20.697131000
6	1.120139000	24.901313000	19.409605000
6	2.315862000	25.420304000	18.788861000
7	4.734504000	26.522757000	17.464875000
7	7.158571000	26.568159000	18.988754000
7	5.692159000	25.689879000	21.284162000
7	3.355648000	25.417720000	19.685290000
26	5.223272000	26.010889000	19.351035000
1	1.444109000	25.809550000	16.905241000
1	7.482499000	27.586264000	15.763570000
1	8.981120000	26.349296000	21.848286000
1	2.987444000	24.430181000	22.912985000

6	7.008045000	19.767518000	19.607787000
6	7.406960000	21.017128000	19.100469000
6	9.363086000	19.059612000	19.805590000
6	7.997454000	18.803257000	19.965783000
6	5.720282000	19.189330000	19.898350000
6	8.766961000	21.247155000	18.938389000
6	9.743351000	20.291443000	19.281940000
7	7.335115000	17.711132000	20.486480000
1	7.682860000	16.765274000	20.650858000
6	4.378010000	19.793636000	19.597705000
6	3.943230000	19.644480000	18.125313000
7	3.552555000	18.288037000	17.766087000
6	2.534389000	18.059383000	16.890608000
6	2.322850000	16.600618000	16.507883000
8	1.827812000	18.955080000	16.417926000
1	4.081243000	17.485963000	18.116183000
8	9.268734000	22.459840000	18.445930000
6	8.450005000	23.281146000	17.731232000
1	5.298417000	17.171702000	20.748097000
1	6.675160000	21.787683000	18.879720000
1	10.112397000	18.320267000	20.076002000
1	10.792193000	20.534475000	19.142697000
1	3.608974000	19.348407000	20.240409000
1	4.392980000	20.867902000	19.827078000
1	4.761821000	19.989050000	17.475408000
1	3.081224000	20.281870000	17.918145000
1	1.286438000	16.321362000	16.727462000
1	3.007777000	15.924923000	17.026125000
1	2.456355000	16.508325000	15.423855000
1	8.937239000	24.203760000	17.440089000
1	6.625739000	24.284674000	18.733494000
1	7.693250000	22.818128000	17.103855000
16	4.775061000	28.184211000	20.059612000
6	13.051046000	23.368791000	18.053781000
8	14.256863000	23.417907000	17.816629000
7	12.209087000	22.508464000	17.412936000
6	12.706289000	21.541111000	16.440323000
6	13.032337000	22.101942000	15.043525000
6	11.842794000	22.430969000	14.118009000
6	11.026870000	23.650441000	14.575118000
6	12.342533000	22.642556000	12.679006000
1	11.235198000	22.473052000	17.692937000
1	11.959698000	20.741968000	16.364020000
1	13.650865000	21.342566000	14.542441000
1	13.672639000	22.984265000	15.168163000
1	11.171558000	21.557483000	14.111336000
1	10.225382000	23.870244000	13.858988000
1	11.665874000	24.540566000	14.647221000
1	10.564029000	23.495421000	15.553107000
1	11.509301000	22.846404000	11.995502000
1	12.877068000	21.760506000	12.305159000
1	13.031857000	23.496104000	12.627012000
6	9.184379000	18.328116000	24.585602000
6	9.438375000	16.823923000	24.427070000
8	10.119191000	16.187221000	25.231816000
6	10.330248000	19.042867000	25.313369000
6	11.657329000	19.129392000	24.532902000
6	11.552324000	20.018162000	23.283771000
6	12.780461000	19.630537000	25.453871000
1	8.263810000	18.436057000	25.176923000
1	10.509733000	18.503621000	26.250545000
1	10.004766000	20.059261000	25.579214000
1	11.918317000	18.110152000	24.215595000
1	12.509159000	20.054442000	22.748003000
1	10.795606000	19.663715000	22.575933000
1	11.288778000	21.048650000	23.558564000
1	13.740925000	19.672511000	24.925005000
1	12.902157000	18.974391000	26.324137000
1	12.562800000	20.641930000	25.824377000
7	8.830107000	16.253714000	23.352721000
6	8.859771000	14.824881000	23.072732000
6	7.510062000	14.328077000	22.540708000
8	7.258705000	15.032071000	21.334750000
6	6.372268000	14.486217000	23.555558000
1	8.273778000	16.828971000	22.732666000
1	9.134148000	14.313634000	23.999181000
1	7.634598000	13.251591000	22.324158000
1	6.296800000	14.891035000	21.052650000
1	5.424290000	14.189397000	23.099497000
1	6.281797000	15.529677000	23.877137000
1	6.557699000	13.867333000	24.442889000
6	1.949639000	14.282101000	21.151098000
6	0.437593000	14.141324000	21.089751000
8	-0.134911000	13.130262000	20.702915000
6	2.511466000	14.848377000	19.834143000
6	4.043963000	15.082731000	19.828726000
8	4.672129000	14.726117000	20.875442000
8	4.527396000	15.618841000	18.805494000
1	2.252617000	14.929432000	21.982174000
1	2.263999000	14.175290000	19.004341000
1	2.036791000	15.807987000	19.589561000
7	-0.268508000	15.266746000	21.500397000
6	-1.682996000	15.468566000	21.152603000
6	-2.625558000	15.041038000	22.297433000
8	-2.090617000	15.510412000	23.552675000
6	-2.892294000	13.539044000	22.368214000
1	0.283452000	16.112594000	21.570634000
1	-1.923978000	14.922373000	20.237278000
1	-3.571775000	15.575479000	22.164380000
1	-1.135714000	15.311638000	23.488907000
1	-3.531650000	13.314058000	23.229335000
1	-1.959333000	12.974595000	22.452204000
1	-3.396812000	13.201120000	21.455835000
6	-3.031215000	21.669202000	22.557926000
6	-2.267273000	22.975151000	22.289380000
8	-1.041919000	23.049398000	22.446271000
6	-2.208974000	20.686087000	23.389166000
6	-2.499973000	19.219755000	23.094735000
8	-2.906939000	18.821573000	21.993966000
7	-2.250098000	18.354172000	24.102584000
1	-3.998776000	21.887207000	23.024961000
1	-2.321356000	20.883659000	24.462330000
1	-1.150874000	20.868783000	23.155966000
1	-1.847602000	18.684818000	24.967004000
1	-2.261983000	17.343550000	23.917237000
7	-3.031318000	23.994856000	21.853482000
6	-2.461081000	25.235163000	21.334887000
6	-3.571917000	26.257669000	21.046498000
6	-4.648476000	25.703680000	20.086960000
8	-4.787474000	26.286932000	18.962607000
8	-5.306469000	24.706608000	20.477937000
1	-4.011787000	23.859111000	21.585660000
1	-1.893840000	25.036222000	20.415211000
1	-3.116013000	27.157830000	20.625920000
1	-4.066265000	26.525344000	21.989723000
6	-2.661374000	20.403619000	18.015695000
6	-3.979157000	19.696654000	18.276681000
8	-4.881175000	19.711835000	17.394537000
6	-1.435329000	20.003205000	18.857711000
6	-0.281319000	21.007235000	18.636983000
6	-0.995330000	18.560362000	18.550379000
6	0.757527000	20.983451000	19.763359000
1	-2.453685000	20.261166000	16.947884000
1	-1.707961000	20.068494000	19.921501000
1	0.207263000	20.787584000	17.679107000
1	-0.691049000	22.024146000	18.564134000
1	-0.192649000	18.244213000	19.226233000
1	-0.598235000	18.494175000	17.530971000
1	-1.818855000	17.844577000	18.655618000
1	1.578593000	21.674919000	19.548600000
1	0.310540000	21.295516000	20.715421000
1	1.191378000	19.985280000	19.891529000
7	-4.172925000	19.122968000	19.474740000
6	-5.292934000	18.240623000	19.836638000
6	-5.223622000	16.968450000	18.944918000
8	-4.234940000	16.243176000	18.979567000
6	-6.670809000	18.941988000	19.949742000
8	-7.240173000	19.278240000	18.676042000
6	-6.631652000	20.159565000	20.872513000
1	-3.428951000	19.145499000	20.169735000

1	-5.034509000	17.896679000	20.842162000
1	-7.355878000	18.198872000	20.379237000
1	-6.501043000	19.635951000	18.128921000
1	-7.634138000	20.590846000	20.944621000
1	-5.957216000	20.929954000	20.486706000
1	-6.290561000	19.880073000	21.876586000
7	-6.304236000	16.745283000	18.147283000
6	-6.295122000	15.772336000	17.073378000
6	-6.555799000	16.385686000	15.688615000
8	-7.029950000	15.705497000	14.786619000
1	-6.997801000	17.492283000	18.126149000
1	-5.314486000	15.284510000	17.079102000
7	-6.241456000	17.712814000	15.559342000
6	-6.357400000	18.403784000	14.288549000
6	-7.357302000	19.566463000	14.261059000
8	-7.325315000	20.396569000	13.351774000
1	-5.701178000	18.177352000	16.286611000
1	-5.390285000	18.806774000	13.974306000
1	-6.675662000	17.667703000	13.543518000
7	-8.286705000	19.584328000	15.252256000
6	-9.276892000	20.643424000	15.368935000
6	-8.875203000	21.727537000	16.384889000
8	-9.514182000	21.904441000	17.417734000
1	-8.159277000	18.954478000	16.033637000
1	-9.399322000	21.074152000	14.370870000
7	-7.751019000	22.417579000	16.052467000
6	-7.210029000	23.456998000	16.911369000
6	-6.699551000	24.603659000	16.026793000
8	-6.725305000	24.524180000	14.790629000
6	-6.080009000	22.922052000	17.812602000
8	-4.899705000	22.642163000	17.055699000
1	-7.401632000	22.389042000	15.099696000
1	-8.020312000	23.814826000	17.556673000
1	-5.820548000	23.646143000	18.591073000
1	-6.451423000	22.019399000	18.310918000
1	-4.943276000	21.715034000	16.758595000
7	-6.218128000	25.676441000	16.693252000
6	-5.687584000	26.824078000	15.988975000
6	-4.243657000	26.719231000	15.485041000
8	-3.681573000	27.723163000	15.042937000
1	-5.965534000	25.656216000	17.695559000
1	-6.304498000	27.027556000	15.107841000
7	-3.674080000	25.489657000	15.525284000
6	-2.407248000	25.188958000	14.872231000
6	-2.609136000	24.641925000	13.440453000
8	-1.669265000	24.647913000	12.643971000
6	-1.537261000	24.312277000	15.821290000
8	-0.948287000	25.130026000	16.823231000
6	-0.402076000	23.558543000	15.137681000
1	-4.144855000	24.741370000	16.025747000
1	-1.877853000	26.134225000	14.729715000
1	-2.214444000	23.574213000	16.285245000
1	-1.590137000	25.787933000	17.197064000
1	0.203599000	23.070649000	15.907680000
1	0.232707000	24.239209000	14.563573000
1	-0.781868000	22.792364000	14.456653000
7	-3.853825000	24.188531000	13.129031000
6	-4.240188000	23.837003000	11.768504000
6	-4.725121000	22.391058000	11.593901000
6	-3.695331000	21.276776000	11.703874000
6	-4.156456000	19.949528000	11.686950000
6	-2.315086000	21.499561000	11.790159000
6	-3.267939000	18.876331000	11.752719000
6	-1.424285000	20.422321000	11.864114000
6	-1.892502000	19.109431000	11.843971000
1	-4.606270000	24.324147000	13.797131000
1	-3.380557000	24.051084000	11.129318000
1	-5.539775000	22.193109000	12.302951000
1	-5.192805000	22.332986000	10.598610000
1	-5.227868000	19.768513000	11.638750000
1	-1.920818000	22.511025000	11.821685000
1	-3.648808000	17.857248000	11.739406000
1	-0.358176000	20.618029000	11.947400000
1	-1.196540000	18.276976000	11.909085000
1	7.688572000	25.468778000	23.945185000
1	5.141347000	24.648937000	24.400859000
1	9.702534000	27.735235000	17.176497000
1	10.335809000	27.215194000	19.764510000
1	2.735473000	26.755092000	14.806275000
1	5.304108000	27.497672000	14.325303000
1	0.799219000	24.184795000	21.465511000
1	0.181519000	24.792247000	18.880815000
1	8.972953000	18.787007000	23.612513000
1	9.633299000	14.603581000	22.323376000
1	2.377250000	13.294392000	21.336731000
1	-1.827826000	16.534823000	20.967990000
1	-3.255845000	21.211693000	21.587331000
1	-1.748132000	25.633893000	22.064154000
1	-5.055449000	24.505140000	11.460185000
1	-2.880807000	21.474504000	18.130643000
1	13.622898000	21.103309000	16.851294000
1	-5.737582000	27.690948000	16.651437000
1	-10.227454000	20.223693000	15.705986000
8	-2.415847000	27.195348000	17.922436000
1	-2.638680000	27.733090000	17.143906000
1	-3.291466000	26.916751000	18.301288000
1	-7.054016000	14.995581000	17.216698000
6	5.975929000	17.945703000	20.420837000
8	5.658090000	24.343310000	18.880765000
6	12.387082000	24.289715000	19.069978000
1	13.030310000	24.358236000	19.951617000
1	12.304408000	25.295442000	18.641462000
1	11.388051000	23.958750000	19.367443000
1	4.409056000	28.693556000	18.862548000

IM2[2]

6	2.408173000	26.149921000	17.550451000
6	6.968638000	27.645687000	16.919172000
6	8.127608000	26.117701000	21.365571000
6	3.504769000	24.882597000	22.083764000
6	3.566171000	26.667415000	16.987821000
6	3.654410000	27.179661000	15.644255000
6	4.932960000	27.613496000	15.473778000
6	5.628629000	27.360902000	16.711117000
6	7.682311000	27.356410000	18.073603000
6	9.076173000	27.650150000	18.263984000
6	9.408521000	27.211425000	19.513167000
6	8.216613000	26.652701000	20.088298000
6	6.962778000	25.633160000	21.937832000
6	6.877127000	25.101740000	23.273805000
6	5.573040000	24.772447000	23.480774000
6	4.863463000	25.096580000	22.268883000
6	2.812375000	25.127596000	20.910460000
6	1.413392000	24.821086000	20.718683000
6	1.115216000	25.157753000	19.434624000
6	2.326162000	25.677099000	18.849262000
7	4.780850000	26.778541000	17.628960000
7	7.165628000	26.731554000	19.192255000
7	5.724553000	25.613123000	21.329304000
7	3.364566000	25.646018000	19.757569000
26	5.203326000	26.371958000	19.542716000
1	1.500470000	26.114010000	16.954160000
1	7.509441000	28.120132000	16.105491000
1	9.032629000	26.091000000	21.964810000
1	2.944244000	24.460562000	22.912124000
6	6.880242000	19.577416000	19.617057000
6	7.159559000	20.840896000	19.065621000
6	9.283480000	19.096140000	19.817995000
6	7.949726000	18.717139000	19.995549000
6	5.648939000	18.896508000	19.933559000
6	8.489727000	21.198561000	18.875686000
6	9.545242000	20.339875000	19.248463000
7	7.388053000	17.583028000	20.555591000
1	7.809856000	16.665943000	20.696181000
6	4.259483000	19.383317000	19.637477000
6	3.850700000	19.254044000	18.156302000
7	3.559312000	17.886268000	17.751614000
6	2.590307000	17.624185000	16.832048000
6	2.460563000	16.162177000	16.425185000

8	1.862925000	18.494973000	16.343641000
1	4.117287000	17.106429000	18.110898000
8	8.876944000	22.417827000	18.307210000
6	7.913208000	23.113513000	17.517123000
1	5.404302000	16.872780000	20.833416000
1	6.358181000	21.528734000	18.824697000
1	10.101080000	18.439443000	20.104566000
1	10.568628000	20.666796000	19.092407000
1	3.529546000	18.843901000	20.254103000
1	4.171311000	20.444596000	19.909431000
1	4.648113000	19.676632000	17.526634000
1	2.947620000	19.835863000	17.958792000
1	1.431160000	15.833633000	16.605979000
1	3.157989000	15.513085000	16.960695000
1	2.636220000	16.088245000	15.345828000
1	8.509001000	23.815498000	16.921870000
1	7.395594000	22.400809000	16.867788000
16	4.767946000	28.422130000	20.321654000
6	12.559438000	23.420090000	17.672113000
8	13.752948000	23.477041000	17.379254000
7	11.715911000	22.482324000	17.154652000
6	12.205478000	21.418767000	16.284149000
6	12.460764000	21.807948000	14.816482000
6	11.222088000	22.108734000	13.945740000
6	10.616931000	23.495514000	14.217288000
6	11.574090000	21.974618000	12.454809000
1	10.758329000	22.439090000	17.493554000
1	11.477680000	20.600696000	16.334547000
1	13.000144000	20.960652000	14.367574000
1	13.153674000	22.658660000	14.797503000
1	10.456290000	21.351035000	14.175092000
1	9.730456000	23.664517000	13.593489000
1	11.344585000	24.285129000	13.986160000
1	10.325409000	23.615183000	15.263933000
1	10.702201000	22.177155000	11.820733000
1	11.936674000	20.967059000	12.216951000
1	12.361672000	22.687184000	12.174361000
6	8.955922000	18.329816000	24.671166000
6	9.312322000	16.842932000	24.552067000
8	9.986402000	16.262397000	25.403907000
6	10.028131000	19.130081000	25.421775000
6	11.368904000	19.296610000	24.678423000
6	11.242420000	20.163762000	23.416102000
6	12.428960000	19.880613000	25.625124000
1	8.011086000	18.386555000	25.230407000
1	10.214972000	18.615427000	26.371287000
1	9.627472000	20.125483000	25.663583000
1	11.706519000	18.293977000	24.380672000
1	12.210268000	20.258585000	22.907989000
1	10.532706000	19.753081000	22.690218000
1	10.903219000	21.177062000	23.671046000
1	13.399692000	19.981030000	25.123537000
1	12.568081000	19.243123000	26.506562000
1	12.133861000	20.879048000	25.976724000
7	8.801621000	16.221975000	23.455803000
6	8.932719000	14.794489000	23.201081000
6	7.638115000	14.206313000	22.625707000
8	7.387332000	14.883534000	21.404539000
6	6.457591000	14.299830000	23.598956000
1	8.245981000	16.753786000	22.796964000
1	9.201592000	14.314910000	24.145858000
1	7.839183000	13.137926000	22.425915000
1	6.443901000	14.694014000	21.097714000
1	5.545609000	13.942911000	23.113683000
1	6.291970000	15.337858000	23.907937000
1	6.650073000	13.699379000	24.497412000
6	2.078017000	14.107940000	21.192896000
6	0.561403000	14.023374000	21.145201000
8	-0.053134000	13.018804000	20.809612000
6	2.649521000	14.612748000	19.854880000
6	4.187162000	14.812147000	19.836587000
8	4.813540000	14.470822000	20.888774000
8	4.674949000	15.312069000	18.796700000
1	2.410947000	14.769501000	22.001001000
1	2.382781000	13.916471000	19.050522000

1	2.197417000	15.574277000	19.576937000
7	-0.096637000	15.195516000	21.503848000
6	-1.500687000	15.444098000	21.143025000
6	-2.465636000	15.096986000	22.297124000
8	-1.907699000	15.570749000	23.540156000
6	-2.812214000	13.613796000	22.406526000
1	0.491700000	16.019090000	21.531334000
1	-1.762906000	14.878980000	20.245196000
1	-3.381825000	15.677143000	22.146681000
1	-0.959997000	15.339763000	23.475167000
1	-3.462489000	13.446081000	23.272536000
1	-1.910846000	13.002550000	22.505390000
1	-3.335462000	13.280475000	21.502904000
6	-2.922425000	21.713423000	22.549653000
6	-2.205661000	23.047487000	22.292076000
8	-0.982850000	23.163065000	22.446321000
6	-2.057587000	20.745774000	23.355875000
6	-2.325663000	19.274485000	23.063360000
8	-2.729948000	18.870373000	21.963834000
7	-2.058342000	18.413772000	24.070330000
1	-3.889716000	21.893200000	23.033405000
1	-2.146967000	20.939145000	24.431892000
1	-1.009914000	20.950978000	23.097067000
1	-1.662537000	18.750955000	24.935233000
1	-2.060824000	17.402272000	23.888134000
7	-3.004564000	24.046394000	21.870220000
6	-2.474143000	25.310945000	21.368169000
6	-3.617387000	26.295639000	21.075360000
6	-4.661119000	25.712388000	20.097346000
8	-4.808800000	26.302624000	18.977736000
8	-5.285577000	24.687412000	20.470166000
1	-3.977973000	23.879725000	21.593959000
1	-1.889633000	25.140003000	20.453647000
1	-3.189335000	27.215899000	20.669098000
1	-4.132471000	26.536104000	22.014793000
6	-2.494449000	20.435716000	17.969020000
6	-3.804871000	19.720630000	18.243064000
8	-4.707615000	19.721161000	17.361499000
6	-1.276504000	20.112965000	18.854503000
6	-0.141511000	21.126680000	18.585113000
6	-0.797513000	18.663978000	18.649449000
6	0.888081000	21.195516000	19.718605000
1	-2.263420000	20.236244000	16.914860000
1	-1.569604000	20.242019000	19.907116000
1	0.354661000	20.860917000	17.642138000
1	-0.570968000	22.128516000	18.446233000
1	-0.014627000	18.407384000	19.372114000
1	-0.362371000	18.539112000	17.651276000
1	-1.609932000	17.938250000	18.770226000
1	1.700598000	21.884860000	19.465249000
1	0.426193000	21.562302000	20.643464000
1	1.336206000	20.216373000	19.920822000
7	-3.991974000	19.154467000	19.445329000
6	-5.100212000	18.258588000	19.811649000
6	-5.013745000	16.983776000	18.925042000
8	-4.016125000	16.271278000	18.964991000
6	-6.487446000	18.940899000	19.922546000
8	-7.061072000	19.266035000	18.648004000
6	-6.466355000	20.160568000	20.843188000
1	-3.247882000	19.188478000	20.139875000
1	-4.836105000	17.922468000	20.818285000
1	-7.161852000	18.188981000	20.353669000
1	-6.326232000	19.628343000	18.098222000
1	-7.475556000	20.575731000	20.916508000
1	-5.805449000	20.941573000	20.455294000
1	-6.119401000	19.887922000	21.847136000
7	-6.089753000	16.743426000	18.125833000
6	-6.065923000	15.764762000	17.057281000
6	-6.316721000	16.367392000	15.665832000
8	-6.771237000	15.676697000	14.761781000
1	-6.793993000	17.480257000	18.101073000
1	-5.082653000	15.282652000	17.074320000
7	-6.016973000	17.697506000	15.533366000
6	-6.122049000	18.380966000	14.257776000
6	-7.141016000	19.526506000	14.208781000

8	-7.104651000	20.357714000	13.300647000
1	-5.493391000	18.173212000	16.265629000
1	-5.156413000	18.800784000	13.961459000
1	-6.413608000	17.637127000	13.509484000
7	-8.089949000	19.527626000	15.181379000
6	-9.098311000	20.570961000	15.282097000
6	-8.734430000	21.656647000	16.310538000
8	-9.400718000	21.822814000	17.327559000
1	-7.965073000	18.899172000	15.964280000
1	-9.206614000	21.004460000	14.283566000
7	-7.609999000	22.360569000	16.007300000
6	-7.105157000	23.408036000	16.878162000
6	-6.636023000	24.584048000	16.009332000
8	-6.651224000	24.518114000	14.772302000
6	-5.958047000	22.901238000	17.773811000
8	-4.771743000	22.659091000	17.013139000
1	-7.241981000	22.340242000	15.061524000
1	-7.928510000	23.727655000	17.526737000
1	-5.718752000	23.627821000	18.556289000
1	-6.302702000	21.985684000	18.267398000
1	-4.792180000	21.732844000	16.710840000
7	-6.201810000	25.667626000	16.691046000
6	-5.721106000	26.847209000	16.004245000
6	-4.270482000	26.816720000	15.510157000
8	-3.748934000	27.853206000	15.094262000
1	-5.954669000	25.645808000	17.694511000
1	-6.340813000	27.031117000	15.120700000
7	-3.649097000	25.611806000	15.528806000
6	-2.369400000	25.375602000	14.874796000
6	-2.542782000	24.823534000	13.441008000
8	-1.600364000	24.872301000	12.648894000
6	-1.454766000	24.542122000	15.821411000
8	-0.901171000	25.388480000	16.819820000
6	-0.286980000	23.843182000	15.134237000
1	-4.093277000	24.834744000	16.008781000
1	-1.889093000	26.347260000	14.735706000
1	-2.093703000	23.772584000	16.288731000
1	-1.577775000	26.000903000	17.209862000
1	0.343939000	23.386971000	15.903673000
1	0.311551000	24.553119000	14.556836000
1	-0.631914000	23.058536000	14.455796000
7	-3.765204000	24.318319000	13.122659000
6	-4.126832000	23.946234000	11.760771000
6	-4.546974000	22.480006000	11.589311000
6	-3.473407000	21.411095000	11.727468000
6	-3.881547000	20.066695000	11.743449000
6	-2.103080000	21.690211000	11.807590000
6	-2.951400000	19.031503000	11.836578000
6	-1.170585000	20.651046000	11.908400000
6	-1.586259000	19.320541000	11.922331000
1	-4.526730000	24.416636000	13.786912000
1	-3.272772000	24.195169000	11.126856000
1	-5.364926000	22.252995000	12.285713000
1	-4.994156000	22.395192000	10.586484000
1	-4.944933000	19.842741000	11.699223000
1	-1.748214000	22.716695000	11.815257000
1	-3.291671000	17.998144000	11.848834000
1	-0.112909000	20.890422000	11.985705000
1	-0.858161000	18.518367000	12.010226000
1	7.717230000	24.996107000	23.948626000
1	5.116577000	24.337525000	24.360939000
1	9.709860000	28.129980000	17.528402000
1	10.368914000	27.257306000	20.010917000
1	2.833204000	27.189522000	14.939169000
1	5.384445000	28.055923000	14.594472000
1	0.760123000	24.380386000	21.463730000
1	0.186529000	25.063758000	18.885531000
1	8.748077000	18.756732000	23.682984000
1	9.747970000	14.612657000	22.485978000
1	2.469699000	13.111204000	21.407695000
1	-1.598387000	16.508680000	20.921209000
1	-3.146992000	21.262779000	21.575747000
1	-1.784320000	25.729139000	22.108881000
1	-4.968046000	24.577326000	11.444243000
1	-2.738007000	21.506463000	18.017994000
1	13.149234000	21.052742000	16.704757000
1	-5.816872000	27.703277000	16.675685000
1	-10.049380000	20.135763000	15.597414000
8	-2.477986000	27.339605000	17.966987000
1	-2.727278000	27.878149000	17.196939000
1	-3.339089000	27.008234000	18.337249000
1	-6.821625000	14.984579000	17.198793000
6	6.012050000	17.694600000	20.485659000
6	11.910377000	24.415225000	18.625906000
1	12.583467000	24.575940000	19.472883000
1	11.786142000	25.377386000	18.115727000
1	10.931965000	24.088364000	18.989255000
1	3.607517000	28.621432000	19.655318000
8	6.928650000	23.765750000	18.253172000
1	7.291988000	24.567977000	18.668574000

IM2^4

6	2.378796000	26.156540000	17.550665000
6	6.966524000	27.510428000	16.798270000
6	8.105556000	26.220520000	21.331251000
6	3.520899000	24.852713000	22.069937000
6	3.542304000	26.619771000	16.954964000
6	3.626855000	27.061585000	15.587079000
6	4.917357000	27.440347000	15.375693000
6	5.622289000	27.230477000	16.614231000
6	7.670021000	27.292610000	17.973585000
6	9.059728000	27.597084000	18.158193000
6	9.380957000	27.239265000	19.437209000
6	8.188729000	26.712483000	20.036557000
6	6.954465000	25.707016000	21.906478000
6	6.889565000	25.172666000	23.241811000
6	5.600269000	24.787793000	23.449708000
6	4.876346000	25.087890000	22.242193000
6	2.815997000	25.119978000	20.908594000
6	1.418175000	24.819759000	20.726875000
6	1.105281000	25.182229000	19.451171000
6	2.307826000	25.706433000	18.858571000
7	4.769647000	26.724254000	17.569923000
7	7.145029000	26.741753000	19.128858000
7	5.717406000	25.640044000	21.303380000
7	3.351925000	25.667987000	19.759978000
26	5.179530000	26.433982000	19.523580000
1	1.462964000	26.120830000	16.967344000
1	7.511751000	27.930051000	15.958442000
1	9.009096000	26.230267000	21.932623000
1	2.973327000	24.411072000	22.896171000
6	6.885631000	19.590585000	19.605071000
6	7.167772000	20.852898000	19.052472000
6	9.288019000	19.112921000	19.826107000
6	7.953368000	18.732458000	19.993599000
6	5.652890000	18.909191000	19.914774000
6	8.498992000	21.211972000	18.872646000
6	9.552720000	20.355816000	19.256034000
7	7.388945000	17.598719000	20.551131000
1	7.811567000	16.683116000	20.698488000
6	4.264894000	19.395351000	19.610770000
6	3.861523000	19.261079000	18.128523000
7	3.567454000	17.892641000	17.728332000
6	2.595172000	17.628800000	16.812716000
6	2.462264000	16.165590000	16.411383000
8	1.867604000	18.498882000	16.323343000
1	4.123899000	17.113045000	18.090407000
8	8.887936000	22.431847000	18.305755000
6	7.938723000	23.107862000	17.481021000
1	5.404147000	16.886881000	20.817040000
1	6.368373000	21.539515000	18.801715000
1	10.104090000	18.457947000	20.120876000
1	10.576916000	20.683710000	19.107625000
1	3.532096000	18.858655000	20.226370000
1	4.176415000	20.457643000	19.878657000
1	4.662641000	19.678696000	17.500283000
1	2.960738000	19.844489000	17.925118000
1	1.432983000	15.838972000	16.596341000
1	3.160291000	15.517443000	16.947239000

1	2.634646000	16.087619000	15.331800000	6	-2.189846000	23.035135000	22.288043000
1	8.543122000	23.812512000	16.897438000	8	-0.966030000	23.152965000	22.433647000
1	7.450139000	22.382247000	16.823449000	6	-2.030934000	20.730315000	23.344467000
16	4.755131000	28.698950000	20.293024000	6	-2.302184000	19.259725000	23.051496000
6	12.579837000	23.429527000	17.710041000	8	-2.708129000	18.857600000	21.951775000
8	13.776085000	23.484132000	17.428384000	7	-2.035884000	18.397621000	24.057346000
7	11.738801000	22.494611000	17.183577000	1	-3.866753000	21.875145000	23.035459000
6	12.234264000	21.429505000	16.318165000	1	-2.112662000	20.922365000	24.421294000
6	12.504671000	21.817342000	14.852883000	1	-0.985145000	20.935431000	23.078251000
6	11.274971000	22.125113000	13.971733000	1	-1.641324000	18.733046000	24.923470000
6	10.681900000	23.519259000	14.232103000	1	-2.044443000	17.385993000	23.875561000
6	11.635695000	21.979788000	12.483963000	7	-2.994428000	24.033619000	21.876516000
1	10.778241000	22.452650000	17.514294000	6	-2.472099000	25.302251000	21.376414000
1	11.503736000	20.613481000	16.361653000	6	-3.621248000	26.283421000	21.095716000
1	13.043221000	20.966978000	14.408900000	6	-4.667612000	25.699834000	20.120635000
1	13.202281000	22.664276000	14.839744000	8	-4.822906000	26.293885000	19.004065000
1	10.500247000	21.376158000	14.199786000	8	-5.285694000	24.670655000	20.492473000
1	9.800420000	23.693418000	13.602630000	1	-3.969295000	23.865147000	21.606163000
1	11.418605000	24.300233000	14.000242000	1	-1.893858000	25.137190000	20.456782000
1	10.386336000	23.647672000	15.276601000	1	-3.199774000	27.207736000	20.691905000
1	10.770287000	22.187987000	11.842867000	1	-4.131850000	26.516466000	22.039421000
1	11.989470000	20.967352000	12.253733000	6	-2.498231000	20.433568000	17.960709000
1	12.432476000	22.682766000	12.205326000	6	-3.806275000	19.716266000	18.240370000
6	8.934903000	18.329701000	24.689360000	8	-4.713543000	19.717854000	17.363542000
6	9.297215000	16.844752000	24.564182000	6	-1.275012000	20.112113000	18.839336000
8	9.971369000	16.262766000	25.414903000	6	-0.144057000	21.129133000	18.565103000
6	10.002549000	19.130607000	25.445751000	6	-0.793479000	18.664546000	18.629854000
6	11.344664000	19.304658000	24.706564000	6	0.891500000	21.199349000	19.693043000
6	11.218162000	20.174275000	23.445940000	1	-2.272347000	20.236070000	16.905052000
6	12.399436000	19.890871000	25.657784000	1	-1.562815000	20.238964000	19.893650000
1	7.988779000	18.380285000	25.247037000	1	0.347919000	20.865850000	17.619259000
1	10.188769000	18.612844000	26.393691000	1	-0.577060000	22.129923000	18.429639000
1	9.597913000	20.123689000	25.690525000	1	-0.006495000	18.408725000	19.348267000
1	11.687367000	18.304203000	24.407343000	1	-0.362992000	18.541841000	17.629396000
1	12.187278000	20.275205000	22.941463000	1	-1.603578000	17.936761000	18.753714000
1	10.513147000	19.761698000	22.716532000	1	1.701239000	21.890611000	19.435785000
1	10.872970000	21.185257000	23.702125000	1	0.433679000	21.563551000	20.620941000
1	13.371261000	19.996372000	25.159391000	1	1.343374000	20.221207000	19.891427000
1	12.538515000	19.251981000	26.538206000	7	-3.986610000	19.147433000	19.442369000
1	12.099184000	20.887286000	26.010729000	6	-5.092881000	18.250593000	19.812760000
7	8.791533000	16.227120000	23.463741000	6	-5.010776000	16.977446000	18.923407000
6	8.928311000	14.801498000	23.202003000	8	-4.013118000	16.264663000	18.957634000
6	7.636891000	14.213173000	22.619587000	6	-6.479666000	18.932324000	19.932238000
8	7.384848000	14.901297000	21.404812000	8	-7.060227000	19.259537000	18.661434000
6	6.454829000	14.293024000	23.592180000	6	-6.453976000	20.150242000	20.855120000
1	8.235008000	16.759466000	22.806157000	1	-3.238982000	19.180217000	20.133212000
1	9.195914000	14.317526000	24.144888000	1	-4.823438000	17.912641000	20.817349000
1	7.842428000	13.147578000	22.409902000	1	-7.151518000	18.179406000	20.365574000
1	6.442436000	14.711452000	21.094860000	1	-6.328620000	19.623348000	18.108440000
1	5.544763000	13.937341000	23.102427000	1	-7.462764000	20.565337000	20.934240000
1	6.284985000	15.327487000	23.910733000	1	-5.794877000	20.931985000	20.465630000
1	6.648398000	13.684781000	24.485136000	1	-6.102321000	19.875574000	21.856893000
6	2.081395000	14.100083000	21.174657000	7	-6.090001000	16.739388000	18.127925000
6	0.564801000	14.015351000	21.126998000	6	-6.071163000	15.762417000	17.057749000
8	-0.049779000	13.011644000	20.788944000	6	-6.324210000	16.367226000	15.667637000
6	2.652499000	14.614667000	19.840229000	8	-6.778937000	15.677537000	14.762946000
6	4.189173000	14.821581000	19.825291000	1	-6.794522000	17.476141000	18.108196000
8	4.814051000	14.487106000	20.880574000	1	-5.088857000	15.278262000	17.071360000
8	4.677270000	15.321002000	18.785359000	7	-6.025354000	17.697710000	15.536855000
1	2.414637000	14.756118000	21.987055000	6	-6.130306000	18.382674000	14.262028000
1	2.389989000	13.922135000	19.031347000	6	-7.148798000	19.528675000	14.214624000
1	2.196509000	15.575829000	19.567117000	8	-7.111203000	20.362067000	13.308572000
7	-0.093203000	15.186349000	21.489267000	1	-5.501409000	18.172565000	16.269387000
6	-1.497327000	15.436071000	21.129696000	1	-5.164512000	18.802599000	13.966387000
6	-2.461630000	15.085940000	22.283530000	1	-6.422132000	17.639836000	13.512844000
8	-1.901898000	15.554323000	23.527895000	7	-8.099062000	19.527256000	15.185923000
6	-2.809756000	13.602762000	22.387552000	6	-9.107413000	20.570457000	15.288195000
1	0.495083000	16.009826000	21.520025000	6	-8.744729000	21.652451000	16.320894000
1	-1.759896000	14.872983000	20.230714000	8	-9.410203000	21.812766000	17.339367000
1	-3.377303000	15.667710000	22.136401000	1	-7.975279000	18.896463000	15.967110000
1	-0.954757000	15.321484000	23.461298000	1	-9.213799000	21.007123000	14.290851000
1	-3.458807000	13.432226000	23.253916000	7	-7.621705000	22.359612000	16.019973000
1	-1.908900000	12.990107000	22.482364000	6	-7.118493000	23.404464000	16.894883000
1	-3.334919000	13.273855000	21.483421000	6	-6.648840000	24.583229000	16.030015000
6	-2.901831000	21.698760000	22.545777000	8	-6.662727000	24.520850000	14.792812000

6	-5.972102000	22.895251000	17.790142000
8	-4.784734000	22.656846000	17.029873000
1	-7.254011000	22.343950000	15.073986000
1	-7.942762000	23.721673000	17.543491000
1	-5.733805000	23.618942000	18.575638000
1	-6.316607000	21.977631000	18.279994000
1	-4.805225000	21.732408000	16.722116000
7	-6.215777000	25.665025000	16.715325000
6	-5.734328000	26.846567000	16.032340000
6	-4.283293000	26.817245000	15.539337000
8	-3.761366000	27.854524000	15.126155000
1	-5.969368000	25.639930000	17.718819000
1	-6.353316000	27.033230000	15.148888000
7	-3.661525000	25.612373000	15.556098000
6	-2.380542000	25.378871000	14.903833000
6	-2.550400000	24.831372000	13.467840000
8	-1.605719000	24.882018000	12.678525000
6	-1.467757000	24.542383000	15.849270000
8	-0.919026000	25.385125000	16.853809000
6	-0.296850000	23.848342000	15.162422000
1	-4.106034000	24.833826000	16.033364000
1	-1.900419000	26.351223000	14.768817000
1	-2.106834000	23.769777000	16.311180000
1	-1.596592000	25.999934000	17.239083000
1	0.333240000	23.390124000	15.931340000
1	0.301598000	24.561535000	14.588940000
1	-0.638676000	23.065760000	14.480066000
7	-3.772170000	24.327787000	13.144920000
6	-4.130523000	23.959657000	11.781079000
6	-4.550516000	22.494015000	11.604315000
6	-3.477713000	21.424373000	11.742989000
6	-3.886659000	20.080198000	11.757947000
6	-2.107238000	21.702630000	11.824090000
6	-2.957144000	19.044438000	11.851096000
6	-1.175421000	20.662853000	11.924970000
6	-1.591902000	19.332602000	11.937933000
1	-4.535224000	24.424481000	13.807657000
1	-3.274891000	24.210291000	11.149962000
1	-5.370782000	22.265326000	12.297435000
1	-4.994420000	22.412168000	10.599781000
1	-4.950145000	19.856928000	11.712911000
1	-1.751842000	22.728919000	11.832583000
1	-3.298023000	18.011273000	11.862459000
1	-0.117645000	20.901526000	12.002992000
1	-0.864331000	18.529969000	12.025817000
1	7.733303000	25.102064000	23.916606000
1	5.162806000	24.335260000	24.330459000
1	9.700207000	28.029522000	17.399679000
1	10.337742000	27.316960000	19.937823000
1	2.794994000	27.066104000	14.894637000
1	5.368835000	27.824226000	14.469463000
1	0.771541000	24.365878000	21.469810000
1	0.170732000	25.094661000	18.911062000
1	8.727488000	18.760291000	23.702667000
1	9.746262000	14.626339000	22.488332000
1	2.473090000	13.101857000	21.381966000
1	-1.595085000	16.501190000	20.910504000
1	-3.130967000	21.250565000	21.571799000
1	-1.778712000	25.719908000	22.114079000
1	-4.970828000	24.591817000	11.464338000
1	-2.743477000	21.503785000	18.012605000
1	13.172871000	21.061004000	16.748053000
1	-5.830526000	27.700644000	16.706243000
1	-10.059126000	20.134471000	15.600470000
8	-2.493484000	27.335735000	17.997485000
1	-2.740146000	27.879743000	17.230416000
1	-3.355842000	27.004021000	18.364698000
1	-6.828017000	14.983543000	17.200282000
6	6.013421000	17.708595000	20.471631000
6	11.925338000	24.424295000	18.660700000
1	12.580322000	24.562033000	19.525893000
1	11.832923000	25.394712000	18.159608000
1	10.932804000	24.111266000	18.996821000
1	3.427195000	28.655966000	20.041471000
8	6.922765000	23.753167000	18.178735000
1	7.262723000	24.559695000	18.604575000
